# Identification and functional annotation of long intergenic non-coding RNAs in Brassicaceae

**DOI:** 10.1093/plcell/koac166

**Published:** 2022-06-06

**Authors:** Kyle Palos, Anna C Nelson Dittrich, Li’ang Yu, Jordan R Brock, Caylyn E Railey, Hsin-Yen Larry Wu, Ewelina Sokolowska, Aleksandra Skirycz, Polly Yingshan Hsu, Brian D Gregory, Eric Lyons, Mark A Beilstein, Andrew D L Nelson

**Affiliations:** The Boyce Thompson Institute, Cornell University, Ithaca, New York, USA; The Boyce Thompson Institute, Cornell University, Ithaca, New York, USA; The Boyce Thompson Institute, Cornell University, Ithaca, New York, USA; Department of Horticulture, Michigan State University, East Lansing, Michigan, USA; The Boyce Thompson Institute, Cornell University, Ithaca, New York, USA; Department of Biochemistry and Molecular Biology, Michigan State University, East Lansing, Michigan, USA; Max Planck Institute for Molecular Plant Physiology, Potsdam, Germany; The Boyce Thompson Institute, Cornell University, Ithaca, New York, USA; Department of Biochemistry and Molecular Biology, Michigan State University, East Lansing, Michigan, USA; Department of Biology, University of Pennsylvania, Philadelphia, Pennsylvania, USA; The School of Plant Sciences, University of Arizona, Tucson, Arizona, USA; The School of Plant Sciences, University of Arizona, Tucson, Arizona, USA; The Boyce Thompson Institute, Cornell University, Ithaca, New York, USA

## Abstract

Long intergenic noncoding RNAs (lincRNAs) are a large yet enigmatic class of eukaryotic transcripts that can have critical biological functions. The wealth of RNA-sequencing (RNA-seq) data available for plants provides the opportunity to implement a harmonized identification and annotation effort for lincRNAs that enables cross-species functional and genomic comparisons as well as prioritization of functional candidates. In this study, we processed >24 Tera base pairs of RNA-seq data from >16,000 experiments to identify ∼130,000 lincRNAs in four Brassicaceae: *Arabidopsis thaliana*, *Camelina sativa, Brassica rapa*, and *Eutrema salsugineum*. We used nanopore RNA-seq, transcriptome-wide structural information, peptide data, and epigenomic data to characterize these lincRNAs and identify conserved motifs. We then used comparative genomic and transcriptomic approaches to highlight lincRNAs in our data set with sequence or transcriptional conservation. Finally, we used guilt-by-association analyses to assign putative functions to lincRNAs within our data set. We tested this approach on a subset of lincRNAs associated with germination and seed development, observing germination defects for Arabidopsis lines harboring T-DNA insertions at these loci. LincRNAs with Brassicaceae-conserved putative miRNA binding motifs, small open reading frames, or abiotic-stress modulated expression are a few of the annotations that will guide functional analyses into this cryptic portion of the transcriptome.

## Teaser

An extensive effort to identify and functionally annotate long intergenic noncoding RNAs in four plant species within the mustards, including *Arabidopsis thaliana* and *Brassica rapa.*

IN A NUTSHELL **Background:** All plants have thousands of genes in their genomes that contribute to plant form and function. While the functional “end-state” of many of these genes is proteins, some genes produce RNAs that never produce a protein, but instead function as an RNA molecule. These nonprotein coding RNAs (ncRNAs) are typically separated into two classes based on length: small ncRNAs and long ncRNAs (lncRNAs). Due to their low abundance, poor sequence conservation, and lack of obvious functional domains, lncRNAs are less studied compared to protein-coding genes. Despite these difficulties, some lncRNAs have been shown to be critical in regulating how plants grow and respond to changes in their environment.

**Question:** How many lncRNAs are present in plants, and what are their characteristics and functional roles? To start answering these questions, we examined thousands of RNA sequencing data sets from four mustards, including the model organism *Arabidopsis thaliana* (thale cress), a relative with high seed-oil content, *Camelina sativa*, the species that gives us bok choy and turnips, *Brassica rapa*, and the salt-tolerant mustard *Eutrema salsugineum*.

**Findings:** We found evidence for thousands of lncRNAs in each of the four species. These lncRNAs are often very tissue or context (stress) specific. Of the identified lncRNAs, we highlighted those that were unusually conserved or contained elements that might contribute to function. We also proposed functions for some lncRNAs based on patterns of abundance across tissues/conditions. Using this approach, we uncovered a set of lncRNAs that appear to be important for seed germination in Arabidopsis.

**Next steps:** Using the lncRNA resources generated in this project, we are examining the functions of those that we believe are critical for germination or responses to environmental stresses. In addition, we are expanding our identification efforts to other systems, including agriculturally significant species within the grasses.

## Introduction

As genomic and transcriptomic analyses have become more prevalent, it has become clear that genomes are not solely composed of protein-coding genes, housekeeping RNAs, and transposable elements. One particularly important set of findings came from the Human ENCODE (ENCODE Project Consortium, 2012) project where it was discovered that over 60% of the human genome is transcribed at some point in development into long noncoding RNAs (lncRNAs). The term lncRNA refers broadly to a class of transcripts united by two key features: a length > 200 nt and poor protein-coding potential (i.e. low likelihood of being translated). The term lncRNA is further subdivided into transcripts that are natural antisense (NAT-lncRNAs), intergenic (lincRNAs), sense overlapping (SOT-lncRNAs), and intronic (int-lncRNAs). Each of these classes of lncRNAs appears in analyses of RNA-sequencing (RNA-seq) data because they share features with mRNAs (e.g. they are capped, polyadenylated, and often multi-exonic; Guttman et al., 2009). Most lncRNAs were missed or ignored in earlier expressed sequence tag (EST)-based screens because of their low or tissue-specific expression and lack of open reading frames (ORFs). However, RNA-seq data from more than 37,000 experiments reflecting ∼60 tissues under different experimental and developmental conditions led to the identification of > 100,000 high confidence (HC) lncRNAs in humans (Volders et al., 2013; Zhao et al., 2016, 2021).

In contrast to proteins, which were the focus of study long before the genomes from which they are encoded were sequenced, an appreciation for the abundance and varied roles of lncRNAs has primarily emerged along with the accumulation of sequencing data. As a result, the catalog of functionally characterized lncRNAs is limited, both in number and in diversity of organisms where they have been annotated (Seifuddin et al., 2020; Chekanova, 2021; Statello et al., 2021). Moreover, the extent to which functionally characterized lncRNAs are archetypal across plants, animals, and fungi is unknown. Not surprisingly, lncRNA identification and functional characterization lags far behind similar efforts for proteins, representing a fundamental gap in our understanding of how genomes operate.

Findings from across eukaryotes serve to illustrate the importance of lncRNAs to genome stability and regulation. Prominent mammalian examples include the telomerase RNA component (TERC), a scaffolding RNA that is crucial for chromosome maintenance (Feng et al., 1995); XIST, a guide RNA responsible for X chromosome inactivation (Brown et al., 1992); and HOTAIR, a developmentally linked signaling RNA (Gupta et al., 2010). In Arabidopsis, TERC has been characterized, with sequence and structural homologs present across the plant lineage, highlighting the potential for lncRNA conservation over long evolutionary timescales (Fajkus et al., 2019; Song et al., 2019; Dew-Budd et al., 2020). Most other functionally characterized lncRNAs in plants, such as COOLAIR, ELENA1, SVALKA, MAS, APOLO, and HID1, change expression or function in response to environmental cues and can thus be classified as environmental sensors (Csorba et al., 2014; Wang et al., 2014; Seo et al., 2017; Zhao et al., 2018; Kindgren et al., 2019; Ariel et al., 2020). These examples reflect the myriad of different mechanisms by which lncRNAs play important biological roles in plants, and also likely represent only a small subset of the mechanisms and modes of action of lncRNAs.

One critical factor behind the paucity of functionally described lncRNAs in plants relative to mammalian systems is the lack of annotated candidate lncRNAs available for interrogation. Moreover, across studies where lncRNAs have been annotated, there are disparities in the types of transcriptional data analyzed as well as the criteria used for their classification. In Arabidopsis, most annotation efforts have focused on long intergenic noncoding RNAs (lincRNAs), since they do not overlap with other transcriptional products and thus are easier to distinguish from other classes of lncRNAs. For example, in Arabidopsis, the bulk of annotated lincRNAs are derived from two studies (Amor et al., 2009; Liu et al., 2012), although other genome-wide examinations have been performed (Moghe et al., 2013; Wang et al., 2014). Amor et al. examined full-length cDNA libraries for the lack of coding potential, whereas Liu et al. utilized TILING arrays to infer gene structure and transcriptional status. In both cases, the maximum allowable ORF was 100 amino acids (AA) or less. Other lincRNA identification efforts in select angiosperms with genomic data (e.g. GREENC; Paytuví Gallart et al., 2016) used official genome annotations generated by MAKER (Cantarel et al., 2008) without direct transcriptional evidence, and maximum allowable ORFs of 120 AA (Jin et al., 2021). Yet in other plant systems, lincRNA identification efforts are limited to a few tissues or developmental stages (Qi et al., 2013; Moghe et al., 2013; Li et al., 2014; Shuai et al., 2014). In summary, the disparity in identification schemes and discordant developmental stages and environmental conditions makes it difficult to make sequence or structural-based comparisons within and across species, as is typically done for protein-coding genes.

Here, we present a comprehensive and unified annotation of lincRNAs, using criteria established for mammals, across four models or agriculturally important Brassicaceae: *Arabidopsis thaliana*, *Camelina sativa* (false flax), *Brassica rapa* (field mustard), and *Eutrema salsugineum* (saltwater cress). We reprocessed the data from more than 16,000 different publicly available RNA-seq experiments (> 24 Tera base pairs of raw data) and generated our own Oxford Nanopore (ONT) and Illumina RNA-seq data, to identify lincRNAs in each of these species. We focus primarily on the intergenic class of lncRNAs for evolutionary and technical reasons: the evolution of NAT- and SOT-lncRNAs is expected to be heavily influenced by the protein-coding genes they overlap, and the unstranded nature of much of the publicly available RNA-seq data makes confident strand assignment of single-exon transcripts difficult. Using transcriptomic, proteomic, epigenetic, and genome-wide RNA–protein interaction data, we examined our lincRNA catalog for features that distinguish lincRNAs from other transcriptional units. We used evolutionary and comparative genomic approaches, leveraging the unique strength of plant polyploidy, to identify conserved lincRNAs among the four species and the rest of the Brassicaceae as well as to identify conserved motifs for functional testing. Finally, we used all of these contextual clues, as well as guilt-by-association techniques, to assign putative function to lincRNAs within our catalog.

## Results

### Identification of lincRNAs in four species of Brassicaceae

**Figure 1:**
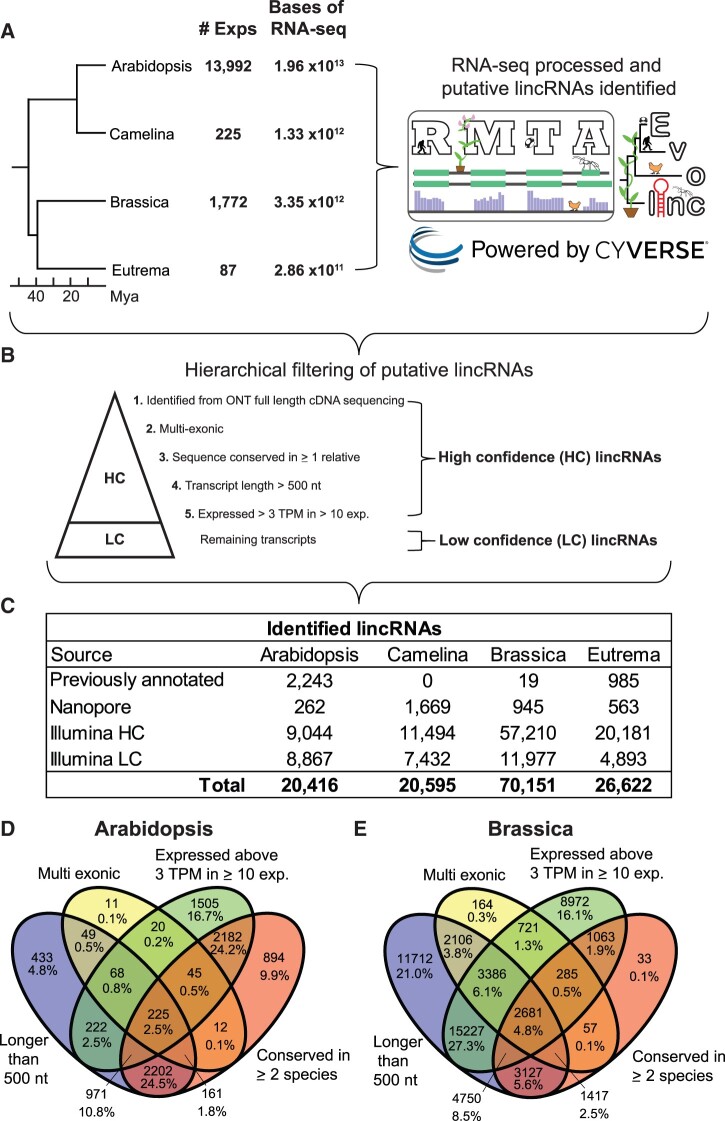
Basic identification and characterization of lincRNAs in each of the four focal Brassicaceae. A, Number of experiments and RNA-seq data processed for each species using the RMTA and Evolinc pipelines in CyVerse’s cloud computing infrastructure. Mya, millions of years ago. B, The metrics used for lincRNA hierarchical filtering. Note, lincRNAs only had to pass one additional filter to be considered a HC-lincRNA. C, The number of lincRNAs identified in each species. Illumina represents publicly available experimental data identified in the NCBI SRA. D, E, Venn diagrams of overlap in the Illumina HC group of Arabidopsis (D) and Brassica (E) lincRNAs using different hierarchical filters.

We processed all RNA-seq data deposited to the Sequence Read Archive (SRA) at the NCBI (accessed December 2018) for *A.thaliana*, *B.rapa*, *C.sativa*, and *E.salsugineum* (hereafter Arabidopsis, Brassica, Camelina, Eutrema) with the goal of detecting the full suite of lincRNAs, including those with low-expression and/or tissue/environmental specificity. We excluded SRAs with epigenetic mutants, degradome experiments (GMUCT and PARE), small RNA-seq, and experiments with low sequencing depth (fewer than 1 million quantified/mapped reads; Figure 1). In addition to publicly available short-read RNA-seq, we also performed Oxford Nanopore Technology (ONT) PCR-free cDNA sequencing on three tissues (10-day seedlings, 4-week mature rosettes, and open flowers) for all four species. We used previously developed workflows (RMTA, Peri et al., 2019) utilizing the CyVerse computational infrastructure (Merchant et al., 2016) to map, in high throughput, ∼24 terabases of RNA-seq data associated with 16,076 experiments (listed in Supplemental Data Set S1). We then identified initial candidate lincRNAs using the Evolinc computational pipeline (Nelson et al., 2017).

We filtered these candidates based on a set of heuristic filtering steps similar to those used by Cabili et al. (2011) to identify the gold standard set of human lincRNAs (Figure 1B). Transcripts were considered high confidence lincRNAs (HC-lincRNAs) if they met the criteria for one of the filters. The first filter selected for lincRNAs identified by ONT cDNA sequencing because of the potential for capturing full-length lincRNA transcripts (Seki et al., 2019). ONT cDNA sequencing across the three tissues yielded 262 unannotated (i.e. not present in the Araport 11 annotation) lincRNAs in Arabidopsis, 945 in Brassica, 1,669 in Camelina, and 563 in Eutrema. These lincRNAs were also present in the Illumina sequencing data, and thus had multiexperiment support. Our next filters focused on lincRNAs only present in the Illumina data. First, we retained lincRNAs as HC if they were multiexonic. This filter selects for transcripts that are less likely to be artifacts of transcript assembly algorithms (Cabili et al., 2011). By this criterion, 678 Arabidopsis, 12,422 Brassica, 6,200 Camelina, and 1,812 Eutrema multi-exonic lincRNAs were identified and annotated as HC (Figure 1C). Some previously identified and characterized lincRNAs are mono-exonic (West et al., 2014; Sun and Ma, 2019; Lorenzi et al., 2021). Thus, we designated as HC mono-exonic lincRNAs that met one of the following filtering criteria: (1) the transcript was conserved in sequence and syntenic in at least one additional Brassicaceae genome outside of the query (see Materials and methods); (2) the transcript length was > 500 nucleotides (nts); or (3) the transcript was expressed at > 3 transcripts per million (TPM) in at least 10 RNA-seq experiments from different bio-projects. All Evolinc candidate lincRNAs that did not pass these filters were retained within our data set as low confidence lincRNAs (LC-lincRNAs), since there is greater potential for these transcripts to be artifacts. In total, we identified 9,306 Arabidopsis, 58,155 Brassica, 13,163 Camelina, and 20,744 Eutrema HC-lincRNAs (Figure 1, C–E; Supplemental Figure S1, A and B), while 8,867, 11,977, 7,432, 4,893 lincRNAs were categorized as LC-lincRNAs, respectively (Supplemental Data Set S2).

While we did not observe any significant difference between HC- and LC-lincRNAs with respect to proximity to the nearest protein-coding gene or transposable element (Supplemental Figure S1, C and D), the potential exists for misassembled or fragmented mRNAs or mRNAs with poorly annotated extensions at the 5′ or 3′ end to be misclassified as lincRNAs. To determine the frequency at which we misclassified these transcripts as lincRNAs, we compared independently assembled transcriptomes from Illumina short-read and ONT long read datasets, searching for short read-derived lincRNAs that mapped to the 3′ or 5′ extension of a coding transcript from our ONT sequencing data. Using this approach, we identified 39 lincRNAs in Arabidopsis that shared at least 1 ONT sequencing read on the same strand as a neighboring mRNA (out of 2,370 lincRNAs for which we obtained ONT coverage ≥ 1 mapped read). Of the 39 lincRNAs with overlapping sequence reads, only two appeared to be mRNA extensions (Supplemental Figure S1E). The other 37 lincRNAs shared sequencing reads due to mis-assembly or genomic DNA contamination in the sequencing (Supplemental Figure S1, F and 1G, asterisks), or are larger variants of Araport lncRNAs. In general, we identified strong agreement between ONT and Illumina-derived lincRNA transcript models (Supplemental Figure S1H), suggesting that the depth of Illumina sequencing used here was more than sufficient to overcome mis-assembly common for transcripts expressed at low levels. Given the low rate (1.64%) of false positives, we remain confident that the transcripts we have identified are indeed independently transcribed elements within the Arabidopsis genome. We also assessed whether these transcripts might represent unannotated miRNA precursors. To assess this, we scanned each of the genomes using rFAM and then intersected those data with the lincRNA catalogs. We identified 26 potential miRNA precursor lincRNAs for Arabidopsis, 69 for Camelina, 124 for Brassica, and 44 for Eutrema. These lincRNAs are annotated with the putative miRNA motif (e.g. MIR172) in Supplemental Data Set S2.

### Harmonizing Arabidopsis lincRNA annotations from multiple sources

We next assessed how many previously identified Arabidopsis lncRNAs were expressed in our assembled RNA-seq data. Given the comprehensive nature of our data set, we presumed that a previously annotated lncRNA was misannotated if we did not observe expression above 1 TPM in at least 10 Arabidopsis RNA-seq data sets (out of all Arabidopsis RNA-seq data examined). The Araport11 annotation contains multiple classes of transcripts that might be considered lincRNAs, including long noncoding RNAs (lnc_RNAs), noncoding RNAs (ncRNAs), and novel transcribed regions, which are a miscellaneous class of uncharacterized transcripts in the Araport11 build. The Araport11 annotation includes 2,455 lnc_RNAs, 286 ncRNAs, and 726 novel transcribed regions. To create a uniform data set of lincRNAs, we filtered out transcripts that did not fit the most basic definitions of a lincRNA (over 200 nt, not overlapping a protein-coding gene), and for which we did not observe expression. Of the 2,455 “lnc_RNAs”, 401 were not classified as intergenic because they overlapped a protein-coding gene, and 157 were relabeled as low confidence (LC-Araport) due to the lack of sufficient expression levels based on our expression filtering (> 1 TPM in 10 experiments from different bioprojects). However, we did observe low levels of expression (> 0.1 TPM) for some of these LC-Araport lincRNAs in various tissue expression atlases, stress data sets, or our Nanopore sequencing data (Table 1). In total, we confirmed 1,897 Araport lnc_RNAs to be HC-lincRNAs. For the 286 annotated ncRNAs, 189 (66%) passed the length, intergenic, and expression criteria, and thus were also considered to be HC-lincRNAs. Finally, we analyzed the novel transcribed regions. We treated these transcripts to the same set of filters as our lincRNA data set: ORF < 100 AA, longer than 200 nts, and poor coding potential (as determined by the coding potential calculator, CPC2, Kang et al., 2017), which resulted in 571 NTRs annotated as HC-lincRNAs, which we included in further analyses. In total, we reannotated 2,657 of the 3,467 Araport lncRNAs as HC-lincRNAs (Supplemental Data Set S2), while the remaining 901 loci were reannotated as LC-lincRNAs or were discarded as not fitting the definition of a lincRNA (Supplemental Data Set S3). We also compared the Evolinc/Araport lincRNAs against other recent Arabidopsis lincRNA data sets not present in Araport (Ivanov et al., 2021; Zhang et al., 2021). Out of the 692 Evolinc lincRNAs that were also identified in the PacBio sequencing performed by Ivanov et al. (2021) 635 (92%) were HC-lincRNAs. Of the 47 lincRNAs that are found within the single-cell RNA-seq performed by Zhang et al. (2021), 32 were considered to be HC-lincRNAs. This overlap is indicated in Supplemental Data Set S2.

**Table 1:** Broad categories of experiment/tissue in which highly context-specific lincRNAs were found to be expressed in Arabidopsis and Brassica

Broad category	Arabidopsis	Brassica
Embryo-associated	468	143
Dissected flower tissue	648	77
Biotic infection	215	NA
Epigenetic mutants	47	15
Root tip or meristem	3,448	NA
Shoot meristem	1,788	NA
Mixed accessions/ssp.	363	21,840
RILs	NA	19,097
Genetic mutants	2,089	NA
Circ-seq	452	NA
Other	2,773	2,202
Total	12,291	43,374

### Annotating lincRNAs based on sequence and structural motifs

**Figure 2:**
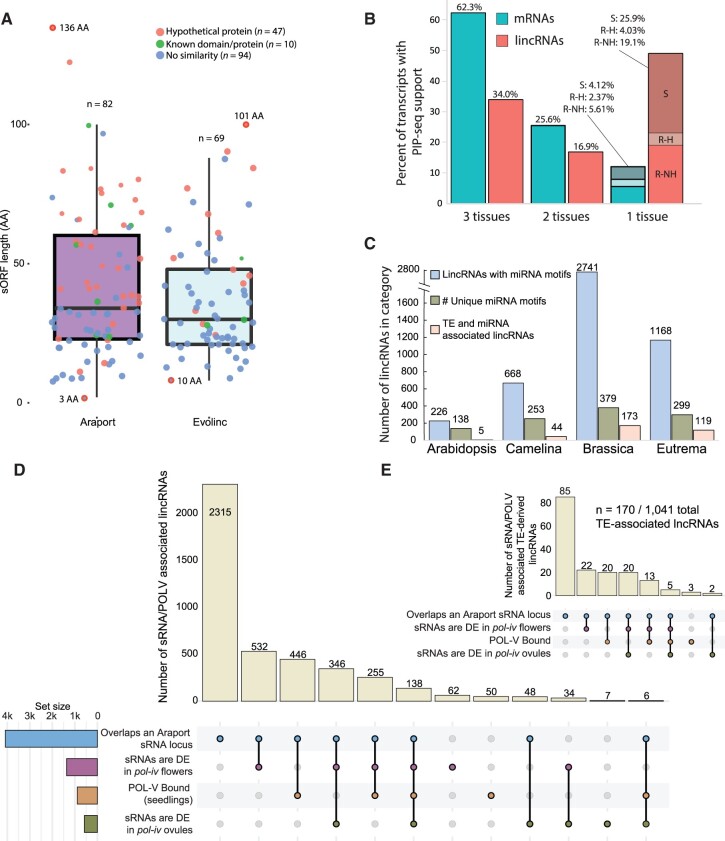
Identification of functional motifs within Arabidopsis lincRNAs. A, Distribution of the length of the newly identified sORFs within the Araport and Evolinc lincRNA populations. The length of the largest and smallest sORFs are denoted (in amino acids, AA). Coloring within the dot represents BLASTp results of each sORF. B, Distribution of identified structured and protein-bound lincRNAs within the total Arabidopsis lincRNA data set based on PIP-seq data from three different tissues (S = Seedling, R-H = Root with hairs, R-NH = Root with no hairs; see Gosai et al. (2015) and Foley et al. (2017) for more details). The percentage of unique mRNAs or lincRNAs that overlap with at least one PIP-seq read are shown. C, The total number of lincRNAs with at least one putative miRNA binding site in each of our focal species (left bar, blue). The middle bar (green) represents the number of distinct miRNAs that interact with lincRNAs. The right bar (pink) represents the number of distinct miRNAs that interact with lincRNAs. The pink bar represents lincRNAs that both contain miRNA binding sites and overlap predicted or annotated TEs. D, UpSet plot representing the lincRNAs that are either associated with sRNA loci under different conditions or are bound by POL-V. sRNAs differentially expressed in a *pol-iv* mutant background are relative to paired wild-type controls. The set size represents the total number of lincRNAs overlapping that category. The colored circles represent overlapping comparisons between the different categories. E, UpSet plot showing the same categories as (D) but only considering lincRNAs that also overlap an annotated or predicted transposable element.

The definition used by the community to distinguish between lincRNAs and proteins is arbitrarily set at a length of 100 AA. Thus, transcripts annotated as lincRNAs may encode small proteins. For example, a few studies have identified previously annotated lincRNAs that are bound to ribosomes and, in some cases, generate detectable protein products (Ji et al., 2015; Hsu et al., 2016; Wu et al., 2019). We used Ribo-seq (Ingolia et al., 2009; Wu and Hsu, 2021) and protein mass spectrometry (MS; Domon and Aebersold, 2006) data from Arabidopsis seedlings (PRIDE: PXD026713) to identify translated small ORFs (sORFs) and protein products within our lincRNAs. Of the 1,172 lincRNAs expressed > 0.1 TPM (length scaled) in seedling tissue, 120 appeared in Ribo-seq data and 38 in MS data, ranging in size from 3 to 136 amino acids (Figure 2A). There was no correlation between transcript and sORF length (Supplemental Figure S2), but we did observe a tendency for Araport11 (*n* = 81) HC-lincRNAs to contain longer sORFs than Evolinc-derived lincRNAs (*n* = 77; *P=* 0.046, Student’s *t* test; Figure 2A). A scan against the NCBI protein databases revealed that 94 of 151 sORFs shared no similarity with known proteins or functional domains, 47 corresponded to hypothetical proteins, and 10 shared similarities with known domains (Figure 2A). LincRNAs containing sORFs are indicated as such in Supplemental Data Sets S2 and S4, but as they reflect previously unidentified genes that would otherwise have been called lincRNAs, they were retained as a separate set of transcripts for downstream analyses.

We next used publicly available transcriptome-wide protein-interaction profile sequencing (PIP-seq; Gosai et al., 2015; Foley et al., 2017) data from roots (with and without root hairs: designated hair and nonhair, respectively) and seedlings (GEO accession numbers GSE58974 and GSE86459) to identify lincRNAs in our data set for which we can infer protein-dependent and independent RNA structural motifs. Across the three data sets, we identified 397 structured and protein-bound Arabidopsis lincRNAs. One hundred thirty-five (34%) of these were present in all three data sets, whereas 195 were restricted to a single cell type/tissue (Figure 2B). Of these cell type or tissue-restricted lincRNAs, 119 were found to be structured in root cells, with the vast majority (103; 26% of structured lincRNAs) only present in nonhair root cells (R-NH; Figure 2B). In contrast, most mRNAs (62%) were found to be structured in all three tissues, whereas only 6% were restricted to nonhair root cells (Figure 2B). Thus, we have evidence for structural motifs within a subset of the Arabidopsis lincRNA data set, a number that will likely only increase as more PIP-seq data are generated. These lincRNAs were annotated in Supplemental Data Set S2, and the multiple sequence alignment (MSA) files are available in the CyVerse Data Store (see Accession numbers).

Some lincRNAs are known to interact with miRNAs, either in a competitive inhibitory fashion (i.e. miRNA sponge; Zhang et al., 2019) or by directly regulating the lincRNA itself (e.g. TAS1A; Chen et al., 2007; Howell et al., 2007). Using the miRNA binding site prediction tool psRNATarget (Dai et al., 2018), we identified 226 Arabidopsis lincRNAs with at least one putative miRNA recognition site (Figure 2C). These miRNA recognition sites corresponded to 138 distinct (unique) miRNA classes, only five of which fell within our set of 20 putative miRNA precursor lincRNAs (Supplemental Data Sets S2 and S5). Importantly, within this set of lincRNAs, we recovered previously characterized miRNA-regulated lincRNAs such as TAS1A and TAS1B. We identified 668 additional lincRNAs in Camelina, 2,741 in Brassica, and 1,168 in Eutrema that contained a putative miRNA recognition site (Figure 2C; Supplemental Data Set S5).

We next determined if any lincRNAs contained portions of transposable element sequences (TE-associated), as this may affect the epigenetic state, expression, and function of these lincRNAs. We first reannotated TEs in each of the four genomes using EDTA (Ou et al., 2019), and then intersected those TEs with the lincRNA catalog for each species. LincRNAs for which a TE comprised 50% or more of the total lincRNA sequence were called TE-associated (Supplemental Figure S2B). This classification is arbitrary, but may potentially guide functional hypotheses. We identified 1,041 TE-associated lincRNAs in Arabidopsis, 1,707 in Camelina, 5,761 in Brassica, and 2,609 in Eutrema (Supplemental Figure S2B and Supplemental Data Sets S2 and S6). The two largest groups in which these TEs could be classified in Arabidopsis, Camelina, and Brassica were helitrons and repeat regions, whereas the top two groups in Eutrema were retrotransposons and repeat regions (Supplemental Figure S2C).

As many lincRNAs can act as cis or trans-regulators via small RNA pathways (e.g. RNA-directed DNA methylation (RdDM)), we tested to see if the Arabidopsis lincRNA catalog overlapped with previously annotated small RNA loci (Araport11), and whether those small RNA populations were dependent on RNA POL-IV in flowers or ovules (Zhou et al., 2022). In addition, we used RIP-seq data from an experiment examining RNA POL-V bound RNAs, targeting the POL-V subunit NRPE1 in seedlings, to determine if we could identify a direct link between any of the Arabidopsis lincRNAs and the RdDM pathway. A total of 4,086 lincRNAs were associated with sRNA loci (Figure 2D, blue; Supplemental Data Set S6), 845 of which were POL-V bound and thus likely active participants in, rather than targets of, RdDM. Many of these sRNA populations are dynamic and synthesized from POL-IV transcripts, as they are differentially abundant in flowers or ovules when comparing wild type to a *pol-iv* mutant background (Figure 2D, purple and green). As TEs are often targeted by RdDM (Matzke and Mosher, 2014), we also assessed to what degree we could detect sRNA loci overlapping with the Arabidopsis TE-associated lincRNAs. Of the 1,041 TE-associated lincRNAs, 167 overlapped an sRNA locus (Figure 2E; Supplemental Data Set S2), suggesting that either most of these TE-associated lincRNAs are not silenced through RdDM or they are regulated by the pathway in other tissues not represented here. In summary, we used a wealth of public information to improve the genome annotations of four agricultural or model Brassicaceae.

### Fundamental features of Brassicaceae lincRNAs

**Figure 3:**
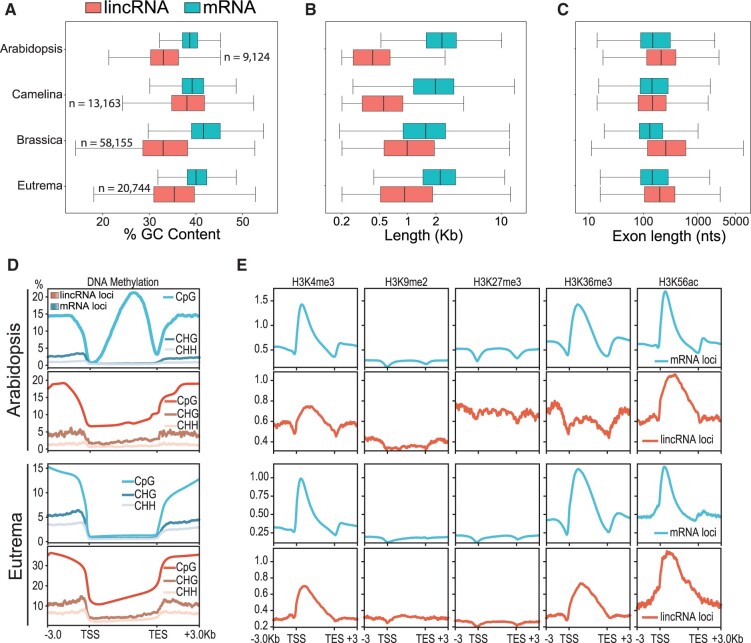
Basic sequence characteristics of Brassicaceae lincRNAs. (A) % GC content, (B) transcript length, and (C) exon length comparisons of mRNAs (blue) and lincRNAs (red) in each of our four focal Brassicaceae. All within-species comparisons of transcript characteristics were significantly different using a pairwise Wilcoxon rank sum test and Bonferroni multiple testing correction. (D) DNA methylation patterns in three sequence contexts (CpG, CHG, and CHH where H is any nucleotide except G) in Arabidopsis and Eutrema lincRNAs and mRNAs expressed > 1 TPM. The *y*-axis represents the weighted average DNA methylation levels over 100-bp bins (i.e. percent methylation) described by Schultz et al. (2016). E, Histone modifications patterns across Arabidopsis and Eutrema mRNAs and lincRNAs expressed > 1 TPM. The *y*-axis represents the averaged counts per million over 100-bp bins.

We examined basic characteristics of our lincRNA data sets with the goal of identifying features that might improve future lincRNA identification efforts. LincRNAs in all four species have significantly lower GC content relative to protein-coding genes (*P-*value for comparison of lincRNA-mRNA for all species < 2.2e−16, Wilcoxon signed-rank test; Figure 3A). Additionally, the transcript lengths of lincRNAs are significantly shorter than mRNAs (*P-*value for comparison of lincRNA-mRNA for all species < 2.2e−16, Wilcoxon signed-rank test; Figure 3B). Interestingly, when we compared multiexonic lincRNAs and mRNAs, we found that the average length of an exon was significantly longer for lincRNAs than mRNAs except in Camelina, where lincRNA exons displayed a similar trend to mRNAs from the other species (*P-*value for all species except Camelina < 2.2e−16, Wilcoxon signed-rank test; Figure 3C). Finally, we analyzed the distribution of exons in lincRNAs in all four species. LincRNAs in Arabidopsis are mostly mono-exonic (∼91.1%), while the lincRNAs identified in the other species have a much more balanced distribution of exon counts. Regardless, lincRNAs in Brassica, Camelina, and Eutrema contain fewer exons on average than the mRNAs in these species (Supplemental Figure S3A).

To better understand the epigenetic mechanisms controlling lincRNA expression, we next examined patterns of epigenetic regulation between lincRNAs and mRNAs. We utilized a comparative genome-wide epigenetic data set generated in Arabidopsis and Eutrema leaf samples (Bewick et al., 2016) to directly compare how lincRNAs and mRNAs are epigenetically regulated in these two species. Using the Bewick data set, we compared CpG, CHG, and CHH (H: any nucleotide except G) DNA methylation for mRNAs and lincRNAs expressed at >1 TPM in leaves (Figure 3D), as well as all genes regardless of expression (i.e. mRNA, lincRNAs, and TEs; Supplemental Figure S3B). In Arabidopsis, lincRNA loci show a consistent decrease in CpG methylation across the lincRNA body, a pattern distinct from that of TEs and protein-coding loci (Figure 3D; Supplemental Figure S3B). Methylation levels at CHG and CHH sites were similarly low for both Arabidopsis mRNA and lincRNAs, regardless of expression. Eutrema is known to lack gene body methylation (Bewick et al., 2016), and thus we observed a lack of gene/lincRNA body methylation in both mRNAs and lincRNAs, although overall methylation rates of all types were higher for lincRNAs than for mRNAs. Increased methylation at lincRNA loci was more pronounced when taking into consideration nonexpressed loci (Supplemental Figure S3B). Both Arabidopsis and Eutrema TE loci showed a pronounced increase in all methylation types, both within the TE body and in the adjacent region (Supplemental Figure S3B). Interestingly, Arabidopsis and Eutrema TE-associated lincRNAs exhibited much higher levels of all methylation types (Supplemental Figure S3B), suggesting these lincRNA loci might be regulated in a similar fashion to TEs. Although the sample size was low, Arabidopsis TE-associated lincRNAs expressed at >1 TPM in leaf tissue exhibited higher levels of CpG methylation around the lincRNA body, whereas Eutrema TE-associated lincRNAs showed consistently higher CpG methylation levels throughout (Supplemental Figure S3, C and 3D). Indeed, Arabidopsis lincRNAs that displayed TE-like methylation patterns were both more tissue specific (high TAU) and expressed at lower levels in any tissue (Supplemental Figure S3, E–G).

Both mRNAs and lincRNAs exhibited similar patterns of H3K4 trimethylation, particularly near the transcription start site, although H3K4 trimethylation was reduced by ∼50% at lincRNA loci in Arabidopsis and Eutrema (Figure 3E). Arabidopsis and Eutrema mRNAs showed a characteristic dip in H3K9 dimethylation and H3K27 trimethylation near the transcriptional start and stop sites. This pattern was not as noticeable in expressed lincRNAs for the two species, although overall methylation levels were similar between mRNAs and lincRNAs (Figure 3E). H3K36 trimethylation was also reduced in lincRNAs relative to mRNAs, whereas H3K56 acetylation showed a similar profile between mRNAs and lincRNAs (Figure 3E). Thus, histone modification profiles were similar between lincRNAs and mRNAs in these two species, although they were somewhat reduced for lincRNAs, whereas in Arabidopsis, lincRNAs lacked the methylation present within mRNAs.

**Figure 4:**
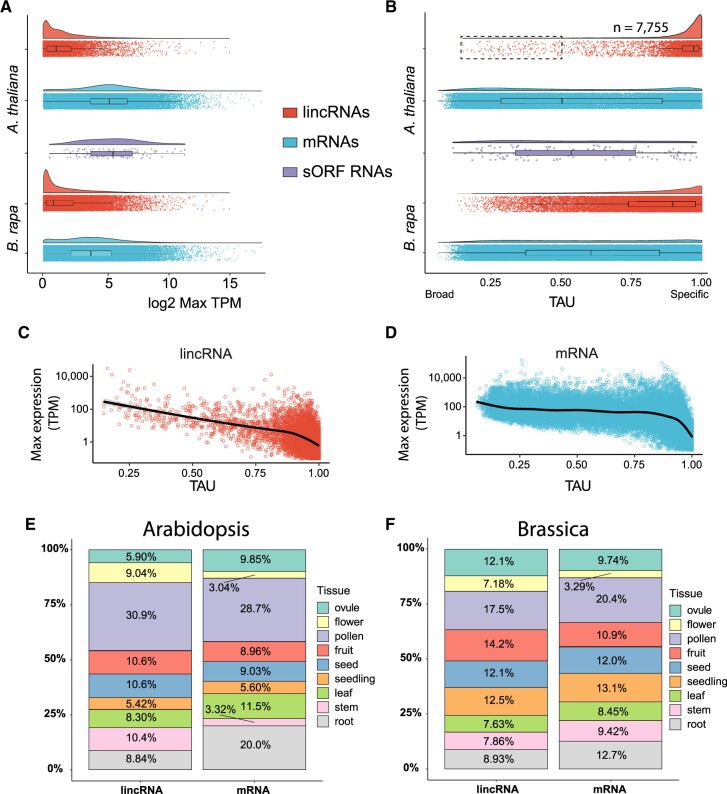
Expression dynamics of Arabidopsis and Brassica lincRNAs. A, Log2 maximum TPM for lincRNAs, mRNAs, and sORF containing lincRNAs (Arabidopsis only) using tissue atlas data for the two species. Note, only genes expressed in the tissue atlas were plotted. sORF-containing lincRNAs and mRNAs are not significantly different (*P* = 0.55, Wilcoxon rank sum test). B, Tissue specificity (TAU) for Arabidopsis and Brassica transcripts across tissue atlas data. The dashed box denotes the 96 Arabidopsis lincRNAs that are below the median TAU value of mRNAs and were inspected further for similarity to protein-coding genes (see text for details). C, D, Correlation between tissue specificity and maximum expression for Arabidopsis lincRNAs (C) and mRNAs (D) within the Klepikova tissue atlas. E, F, Stacked bar charts designating the tissue of highest expression for lincRNA and mRNA repertoires in Arabidopsis and Brassica, respectively.

LncRNAs in mammals are often tissue or cell-type specific and are often expressed at low levels in particular tissues relative to mRNAs. This has also been observed to a certain extent in plant systems, albeit with far fewer tissue comparisons. Maximum lincRNA expression, in any tissue, was ∼10-fold lower compared to mRNAs in three of the four species (Figure 4A; Supplemental Figure 4A), whereas in Camelina, we observed slightly higher expression of lincRNAs relative to mRNAs. Tissue specificity (TAU; Yanai et al., 2005) was determined based on the expression data from tissue atlases in Arabidopsis (Klepikova et al., 2016) and Brassica (Tong et al., 2013; Bilichak et al., 2015), as well as from our ONT RNA-seq data. As expected, lincRNAs from all four species were, on average, significantly more tissue-specific than their respective mRNA cohorts (adj-*P* < 2e−16, Wilcoxon rank sum test with Bonferroni multiple comparison correction; Figure 4B; Supplemental Figure S4B). We also observed a negative correlation between tissue specificity and expression for lincRNAs: more highly expressed lincRNAs were more broadly expressed, a feature that was significantly more pronounced for lincRNAs than for mRNAs (*P* < 2.2e−16, Student’s *t* test performed on Fisher transformation of correlation coefficients; Figure 4, C and D). This negative correlation was observed across multiple tissues (e.g. female reproductive, leaf, and male reproductive; Supplemental Figure S4C), although we did observe tissue-dependent differences, such as high expression associated with high specificity for both lincRNAs and mRNAs in pollen/anther RNA-seq data. The sORF-containing lincRNAs displayed expression and tissue specificity values similar to those of mRNAs (*P* = 0.55, Wilcoxon rank sum test, Figure 4, A and B), further supporting an mRNA assignment. Given this link between lower tissue specificity (broader expression) and coding potential, we more closely examined the Arabidopsis lincRNAs (*n* = 89) with TAU values lower than the median value for mRNAs (TAU < 0.502; Figure 4B, black box). Based on sequence similarity, these broadly expressed lincRNAs do not appear to be recently pseudogenized protein-coding genes, but, for a subset (*n* = 61), expression was significantly correlated with a neighboring gene less than 500 bp away (Supplemental Figure S4D). Thus, high tissue specificity and low expression can be considered a defining feature of Brassicaceae lincRNAs and can potentially help to distinguish unannotated sORF-containing transcripts.

In mammalian systems, a large number of lincRNAs are expressed, or show elevated expression, in male reproductive tissues (Hong et al., 2018). This phenomenon is attributed to relaxed epigenetic control within these tissues (Buljubašić et al., 2018). We sought to determine if this was also a feature of plant lincRNAs by examining lincRNA expression within the Arabidopsis and Brassica tissue atlases. Approximately 45% and 35% of lincRNAs in Arabidopsis and Brassica, respectively, were most highly expressed in reproductive tissues, with pollen being the predominant source of maximum expression levels (Figure 4, E and F). A similar percent of mRNAs showed peak expression in reproductive tissues in the two species, suggesting a general transcriptome-wide, instead of lincRNA-specific, phenomenon. Consistent with this transcriptome-wide phenomenon, lincRNAs restricted to pollen were expressed at significantly higher levels than lincRNAs restricted to other tissues (e.g. female reproductive versus leaf tissue; Supplemental Figure S4C, note scales). To aid in the exploration of lincRNA and mRNA expression between tissues and experiments, these data have been uploaded to the appropriate BAR eFP Browser (Provart and Zhu 2003), and are explorable through an interactive Clustergrammer (Fernandez et al., 2017) Jupyter notebook binder found at https://github.com/Evolinc/Brassicaceae_lincRNAs (Supplemental Figure S5).

Interestingly, 48% and 60.8% of the complete (HC + LC) Arabidopsis and Brassica lincRNA data sets, respectively, were not expressed at > 0.1 TPM in their respective tissue atlas, suggesting these lincRNAs are not expressed under normal conditions during development. Considering that expression was a requirement for identification, we sought to determine where these context-specific lincRNAs (CS-lincRNAs) were expressed. We searched through all of the Arabidopsis and Brassica RNA-seq data looking for experiments examining maximal expression. We extracted metadata from those experiments from the NCBI SRA and grouped the lincRNAs into similar categories based on expression levels (see Materials and methods). In Arabidopsis, the majority of the CS-lincRNAs showed maximal expression in experiments that performed high-resolution sequencing of root or shoot meristems (*n* = 5,236; Table 1), suggesting that these lincRNAs are expressed in very limited cell types. A total of 909 lincRNAs (∼4.5%) were found to be expressed under abiotic or biotic stress conditions (Table 1). In Brassica, the vast majority of the CS-lincRNAs (*n* = 40,937; 57.5%) were maximally expressed in sequencing data from recombinant inbred lines (*n* = 19,097) or hybridization experiments with different Brassica accessions (*n* = 21,840; Cheng et al., 2016), indicating a high degree of transcriptional variation between genetic backgrounds. We also observed a subset of Arabidopsis lincRNAs (∼350) that were only expressed in specific accessions or in crosses between accessions. Finally, 7,407 (10.4%) Brassica CS-lincRNAs were expressed under stress conditions. These data highlight the extreme tissue specificity possible for lincRNAs.

### Evolutionary features of Brassicaceae lincRNAs

Evolutionary conservation is often considered a proxy for the relative importance of the function of a protein-coding gene, and thus we sought to determine the degree to which lincRNAs from each of the four species were evolutionarily conserved in Brassicaceae. We assessed conservation of lincRNAs in two different ways: (1) sequence and synteny-based conservation (sequence homologs), and (2) transcriptional and synteny-based conservation in the absence of identifiable sequence similarity. Conservation at the sequence level may suggest a function dependent on structure or particular motifs being retained, whereas transcriptional conservation may indicate a cis-regulatory role that is independent of sequence conservation.

**Figure 5:**
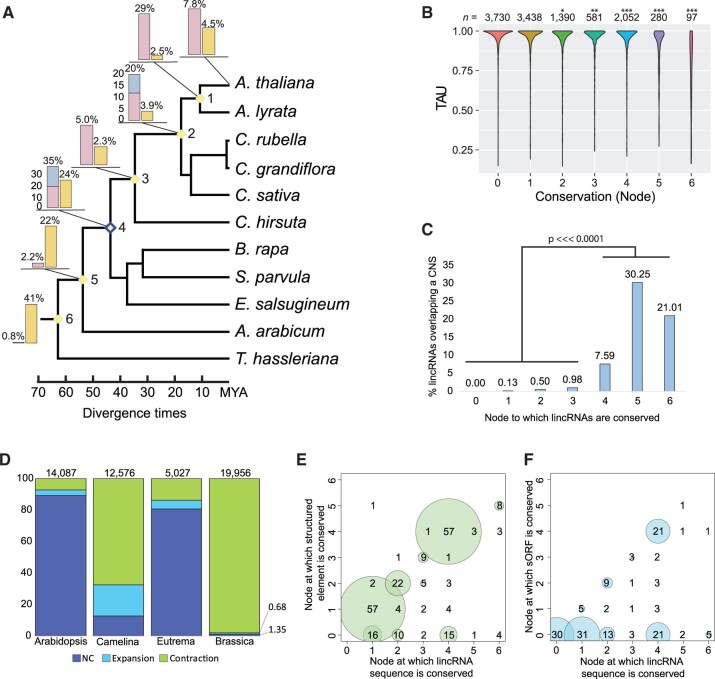
Sequence and transcriptomic conservation of Arabidopsis lincRNAs and their functional motifs across the Brassicales. A, Arabidopsis lincRNA conservation across select Brassicales species. The inset bar graphs depict the percent of Arabidopsis lincRNAs and mRNAs (yellow bar) restricted to that node (out of 20,416 total lincRNAs and 27,173 mRNAs examined). For lincRNAs, pink bars represent lincRNA sequence homologs restricted to that node, whereas blue bars represent transcriptional syntelogs to that node. Identification of transcriptional syntelogs is dependent on transcriptional data from a species descending from that node. B, Tissue specificity metric (TAU, see Figure 4B) for lincRNAs conserved to each node in the phylogenetic tree in (A). Asterisks denote significant differences in TAU at each node relative to node 0 using a Chi-square test for multiple samples. *P <* *0.05, **P <* *0.005, ***P < 0.001. C, Percent of lincRNAs that overlap an annotated CNS. The *x*-axis represents the phylogenetic nodes to which they are conserved in (A). Nodes 4, 5, and 6 are significantly more likely to overlap a CNS relative to nodes 0–3 using both a pairwise Chi-square test with multiple testing-correction (false discovery rate) as well as a post hoc least square means comparisons from a generalized linear model (*P* < 0.0001). D, Percent of lincRNAs in each of the four focal species for which we could infer gene family expansion, contraction, or for which there was no change (NC) relative to at least two of the closest relatives for each species. See Materials and methods for more information. E, Correlation between the nodes at which a lincRNA is conserved (from A) and at which the structured element (from Arabidopsis) is conserved. F, Correlation between the nodes at which a lincRNA is conserved (from A) and the node at which the contained sORF is conserved.

To identify sequence homologs, we used each of the respective sets of lincRNAs as queries in searches of the genomes of nine Brassicaceae as well as *Tarenaya hassleriana*, a member of the sister family Cleomaceae (Cheng et al., 2013b) using Evolinc-II (Nelson et al., 2017). To identify syntenic but sequence divergent (SBSD) lincRNAs, we used SynMap (Haug-Baltzell et al., 2017) to identify collinear blocks between each of our four focal species (Arabidopsis, Camelina, Brassica, and Eutrema) and then searched for lincRNAs transcribed from them (see Materials and methods). Using this combined approach, we determined that 7.8% of Arabidopsis lincRNAs (HC + LC) were species-specific, and a further 29% were restricted to the genus (Figure 5A, Sequence Homologs and SBSD lincRNAs found in Supplemental Data Set S7, with coordinates for sequence homologs in their respective species found in the Supplemental Data on CyVerse). In sharp contrast to lincRNAs, sequence homologs were recovered for ∼41% of Arabidopsis protein-coding genes in *T. hassleriana* (Figure 5A). Species or genera-specific conservation was largely a feature of LC-lincRNAs, which were significantly less likely to be sequence conserved (nodes 0–1; *P* < 0.001, Student’s *t* test with multiple testing correction; Supplemental Figure S6A) than HC-lincRNAs. We determined that 3,725 (18.1%) Arabidopsis sequence homologs and 3,560 (17.3%) SBSD lincRNAs were present at the coalescence point between Brassicaceae lineages I and II (node 4; Figure 5A; Beilstein et al., 2006, 2008). Similar percentages of lincRNAs were recovered at the sequence and transcriptional levels when using Camelina, Brassica, or Eutrema lincRNAs as queries (Supplemental Figure S6, B–D).

Using a reduced stringency homology search, we determined that 959 of the 3,560 node 4 (Arabidopsis → Brassica or Eutrema) SBSD lincRNAs shared short regions of sequence homology, either within the lincRNA gene body (*n *=* *345) or within the promoter region (*n *=* *675; Supplemental Figure S6E, highlighted in Supplemental Data Set S7). The majority of the sequence homologs corresponded to either Evolinc-identified lincRNAs or unannotated intergenic sequences in each of the other species (Supplemental Figure S6F), suggesting that these lincRNAs have been evolving as lincRNAs, and not as pseudogenized loci. As reported in mammalian and plant systems, conservation was strongly associated with broader expression, with homologous lincRNAs displaying similar expression patterns between species (Figure 5B; Supplemental Figure S6G; Hezroni et al., 2015; Nelson et al., 2016). Thus, when considering sequence similarity, only a subset of lincRNAs is conserved throughout the family and thus may depend on sequence for their function. In contrast, a significant proportion of lincRNAs that would have been considered species-specific due to lack of sequence homologs may in fact be transcribed from syntenic loci and therefore harbor cis-regulatory functions.

Sequence conservation associated with intergenic, noncoding regions has been reported in plants and animals (Siepel et al., 2005; Haudry et al., 2013) These conserved noncoding sequences (CNS) are believed to be cis-regulatory elements and display evidence of purifying selection. We tested the Arabidopsis lincRNAs for overlap with the CNS catalog developed by Haudry et al. (2013). At the genome level, we observed a significant correlation between CNS frequency and the set of lincRNAs conserved at the sequence level at node 4 (Supplemental Figure S6H). Indeed, there was a significant association between the node to which a lincRNA was conserved and overlap with a CNS (*P* < 0.0001, Figure 5C; Supplemental Data Set S7). Thus, many of the observed CNS in Brassicaceae appear to be transcribed into lincRNAs and thus may function at both the sequence and transcriptional level to regulate the expression of adjacent genes.

Based on these evolutionary analyses, we asked whether we could identify any previously functionally characterized Arabidopsis lincRNAs, which would suggest that they were conserved outside of Arabidopsis. We examined five functionally annotated Arabidopsis lincRNAs, including the photo-responsive lincRNA *HID1* (Wang et al., 2014; Supplemental Figure S7A), the salt-responsive lincRNA *DRIR* (Qin et al., 2017; Supplemental Figure S7B), the auxin-regulated lincRNA *APOLO* (Ariel et al., 2020; Supplemental Figure S7C), the pathogen resistance-associated lincRNA *ELENA* (Seo et al., 2017; Supplemental Figure S7D), and the cold-responsive lincRNA *SVALKA* (Kindgren et al., 2019; Supplemental Figure S7E). A *HID1* locus was present in all tested Brassicaceae. Surprisingly, we recovered an unreported *HID1* paralog in Arabidopsis. In fact, *HID1* paralogs were present in the genomes of other Brassicaceae (Supplemental Figure S7A). *ELENA* and *SVALKA* sequence homologs were present in Camelina (lineage I) but were not recovered in more distantly related species. Interestingly, we identified SBSD lincRNAs adjacent to multiple *CBF1* loci in Brassica, in a similar orientation and distance to *CBF1* from the Arabidopsis lincRNA *SVALKA* (Supplemental Figure S7E). In total, four putative *SVALKA* lincRNAs were identified next to *CBF1* paralogs in Brassica. We identified two previously unreported *APOLO* paralogs in the genome of Arabidopsis, and multiple paralogs within the *A. lyrata* genome, but were unable to find sequence homologs in other sampled species. None of the *A. lyrata APOLO* homologs was adjacent to the *PID1* locus and thus, if expressed, may not be functionally conserved. Finally, we were unable to identify sequence homologs for *DRIR1* in any Brassicaceae genome sampled. However, we did identify a putative *DRIR1* SBSD locus in Brassica, suggesting that this lincRNA may be functionally conserved outside of Arabidopsis (Supplemental Figure S7B).

Given the apparent expansion of the *HID1* and *APOLO* gene families, we asked how frequently lincRNA gene families expanded or contracted, and whether these dynamics were coincident with known whole-genome duplication (WGD) events. We examined lincRNAs for which we were able to identify a sequence homolog in at least one other organism and asked if the number of lincRNAs in each gene family could have resulted from a WGD event, either from a recent WGD or the α-WGD (Bowers et al., 2003) that coincided with the emergence of the Brassicaceae (see Materials and methods). For Arabidopsis and Eutrema, which have not undergone recent WGDs, lincRNA gene families are predominantly stable (no evidence of paralogs of recent origin for 89% and 81% of lincRNAs respectively; Figure 5D). Camelina and Brassica have both undergone relatively recent whole-genome triplication events (Wang et al., 2011; Mandáková et al., 2019). Despite these WGD events, lincRNAs in both species (67% and 98%, respectively) occurred more frequently in one or two copies rather than three, indicating that paralogs were likely removed via fractionation, which often occurs following WGD (Cheng et al., 2018). Interestingly, this extent of copy loss is greater than the loss observed for protein-coding duplicates (De Smet et al., 2013). Moreover, 71% of Camelina lincRNAs and 85% of Brassica lincRNAs are single copy, suggesting weak selective pressure to retain these genes in multicopy form. In addition, in Brassica, where the least and most dominant subgenomes have been assigned (Tang et al., 2012; Cheng et al., 2013a), most single copy lincRNAs, and most lincRNAs in general, fall within the least fractionated subgenome (LF; *n* = 26,284), vs. the medium fractionated (MF1; *n* = 21,712) and the most fractionated (MF2; *n* = 15,973; Supplemental Figure S8A). For each of these sets of lincRNAs, ∼50% are expressed in data sets generated from intra-specific hybrids or recombinant inbred lines (Supplemental Figure S8A). These data suggest that lincRNAs, like mRNAs, are preferentially retained in dominant subgenomes following WGDs, but lincRNA hybrid-specific expression is not linked to the subgenome of origin.

Camelina has a sufficient number of multicopy lincRNA gene families, allowing us to monitor the impact of WGD events on lincRNA expression. Camelina is an allohexaploid (Mandáková et al., 2019) containing three subgenomes similar to those of its two progenitor species, *C. hispida* and a *C. neglecta*-like autotetraploid (Brock et al., 2018, 2019; referred to here as the *C. hispida*, *C. neglecta*, and *C. neglecta* [like] subgenomes). In Camelina, *C. hispida* mRNA paralogs are typically more highly expressed relative to those from the other two subgenomes, and thus it is considered to be the dominant subgenome (Chaudhary et al., 2020). To explore how WGD has affected lincRNA expression, we performed Illumina short-read RNA-seq in early Camelina embryos (*n* = 5). These data were mapped to the reference genome with an updated gene set (i.e. including lincRNAs). For lincRNA families where all three paralogs are evident, we observed a significant bias against expression of the paralogs from the *C. neglecta* (like) subgenome (*P* < 0.005, pairwise *t* test with multiple testing correction, Supplemental Figure S8B). This is in contrast to lincRNAs that have already been reduced to single copy (Supplemental Figure S8C). For single copy lincRNAs, those transcribed from the *C. neglecta* (like) subgenome showed a slight but significant elevated average expression levels relative to the other two subgenomes (*P* < 0.001; Supplemental Figure S8C). In contrast, paralogous protein-coding genes transcribed from the *C. neglecta* subgenome showed the lowest average expression level relative to the other two subgenomes (Supplemental Figure S8D). Thus, while *C. neglecta* (like) may be considered the least dominant subgenome, single copy lincRNAs retained in this subgenome are expressed at higher levels, suggesting that lincRNAs with different evolutionary paths (multicopy vs. single copy) are expressed at different levels within the same subgenome.

We observed a number of distinct features within the Arabidopsis lincRNA data set, including structured regions, sORFs, and miRNA interaction motifs that may act as functional motifs (Lucero et al., 2020). If these elements are important for lincRNA function, we would expect them to be conserved. Structural elements, inferred from PIP-seq data, strongly and positively correlated with conservation (*P* < 0.01; Figure 5E). Of the 415 lincRNAs for which structured elements were identified, 324 were conserved in another Brassicaceae genome. For 70% of these sequence-conserved lincRNAs, the structured region was conserved to the same node as the lincRNA itself, suggesting the structural element is driving conservation of the lincRNA. An example of this is shown in Supplemental Figure S9A, where the two structural elements of the lincRNA Evolinc_tID.00064432 overlap with deeply conserved (i.e. node 5) portions of the lincRNA.

We next addressed the degree to which the 158 sORF-containing Arabidopsis lincRNAs are conserved, as conservation of the sORF would lend support to the idea that they are actually protein-coding transcripts. Of the 127 sORF lincRNAs conserved outside of Arabidopsis, there was no significant variation in the overall rate of conservation relative to non-sORF lincRNAs, indicating that sORF-lincRNAs are not preferentially retained. Of the 158 sORF lincRNAs tested, 53 sORFs were conserved in at least one other species, and 39 were conserved to the same node as the lincRNAs from which they were derived (see Materials and methods; Figure 5F; Supplemental Data Set S4). There was no clear bias towards the length of the sORF or the encoding transcript, i.e., longer transcripts were no more likely to contain an sORF than shorter ones (Supplemental Figure S2). Some of the conserved sORFs were quite short, such as the sORF within *AT1G06113*, which encodes a nine amino acid peptide and lies within a region of the sORF-lincRNA that shares almost 100% identity across the 11 species present in the MSA (Supplemental Figure S9B). *AT1G06113* is not annotated as a protein-coding gene, nor does its peptide product share similarity with known protein motifs. Although most sORF-lincRNAs (26/36) were previously annotated lincRNAs (i.e. Araport11 lincRNAs), a subset of the sORF-lincRNAs was identified in this study (e.g. Evolinc lincRNAs), suggesting that current filtering schemes are not entirely sufficient for removing short protein-coding transcripts from our dataset.

Finally, we determined the degree to which the predicted miRNA interaction sites within our Arabidopsis lincRNA data set were conserved. Of the 226 lincRNAs with predicted miRNA interaction sites, 68 were species-specific (Supplemental Figure S9C). A further 83 were sequence-conserved in at least one other Brassicaceae, but the conserved region did not overlap with the putative miRNA interaction site. The remaining 75 lincRNAs contained sequence conserved miRNA interaction motifs, with an example for this shown for AT1G50055 (TAS1B) in Supplemental Figure S9D. Multiple sequence alignments supporting our conservation assignments for structure, sORFs, and miRNA interaction sites can be found in the CyVerse Data Store. LincRNAs with conserved domains are annotated in Supplemental Data Sets S2, S4, and S5. In summary, our evolutionary approach has uncovered conserved lincRNA functional elements and sheds additional light on how plant lincRNAs evolve in the face of WGD.

### Assigning putative function to Brassicaceae lncRNAs

Basic characterization of lincRNA expression, along with conservation analysis, can provide clues as to which lincRNAs in our data sets are potentially functional, but these data alone do not permit the formation of robust functional hypotheses. To better clarify when and where the lincRNAs in our catalogs are functioning, we took three approaches. The first was to determine which lincRNAs are stress responsive based on pairwise comparisons of publicly available RNA-seq data (stress vs. control). Secondly, as many lincRNAs regulate the expression of neighboring genes (Khyzha et al., 2019; Gil and Ulitsky, 2020), we examined the correlation of lincRNA-adjacent mRNA gene pair expression across tissue and stress expression atlases to identify candidate gene pairs in which the lincRNA has the potential to regulate its neighbor. Third, we used weighted gene co-expression networks (WGCNA) of larger, more complex experiments to identify modules of similarly expressed protein-coding and lincRNA genes (i.e. guilt-by-association) to infer in which molecular pathway a lincRNA might be acting.

**Figure 6:**
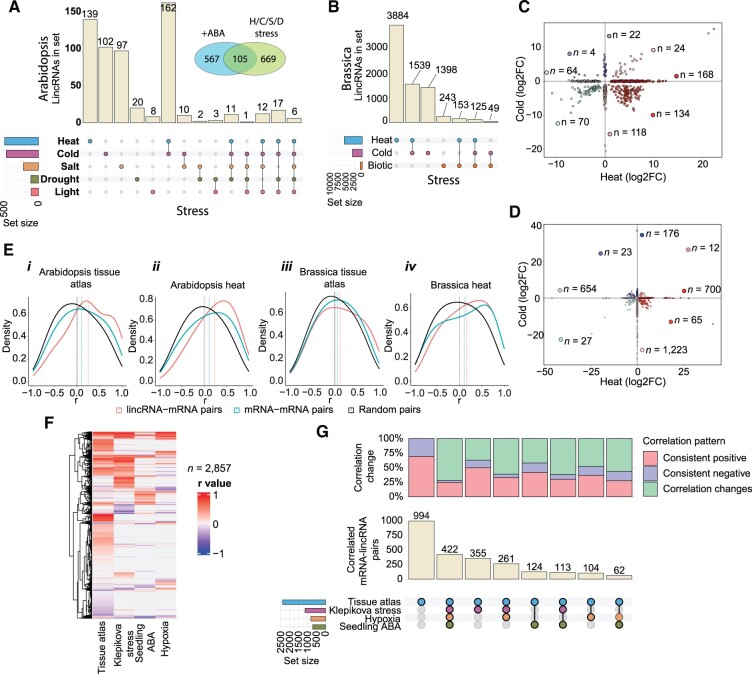
Inferring lincRNA function from transcriptomic data. A, B, UpSet plots depicting the number of stress-responsive lincRNAs in Arabidopsis (A) and Brassica (B). The vertical tan bars depict the number of lincRNAs found in each stress condition, or combination of stress conditions, shown below. The horizontal colored bars depict the total number of lincRNAs associated with that stress across all combinations. For Arabidopsis, an inset Venn diagram depicts the number of lincRNAs found to be both stress (heat, cold, salt, or drought stress, H/C/S/D) and ABA responsive. C, D, Scatterplots of temperature responsive (differentially expressed) lincRNAs in Arabidopsis (C) and Brassica (D). A positive log2FC indicates that the gene was induced in response to the stress. Fold-changes are all relative to paired, nontreated controls. Only lincRNAs differentially expressed under the shortest and longest treatment are shown. All other examined stress experiments are shown in Supplemental Data Set S9. E, Density plots showing the distribution of expression correlation coefficients (Pearson) between different gene pairs in Arabidopsis and Brassica tissue atlases (*i* and *iii*) as well as Arabidopsis and Brassica heat stress experiments (*ii* and *iv*). F, Heatmap of Pearson correlation coefficients for adjacent lincRNA–mRNA gene expression values across four experiments in Arabidopsis. If a lincRNA or mRNA was not expressed in an experiment, no correlation coefficient was computed and therefore is shown with a “0” value. G, UpSet plot (bottom) showing shared and distinct adjacent lincRNA–mRNA pairs in the experiments from (F). Stacked bar plot (top) showing whether the shared correlation coefficients in the UpSet plot below are positive in both (Consistent positive), negative in both (Consistent negative), or change between experiments (Correlation changes).

We first searched for lincRNAs in each species that were differentially expressed in response to stress. For Arabidopsis and Brassica, we chose publicly available data sets with multiple independently generated stress experiments (Supplemental Data Set S8). In both species, most of the stress-responsive lincRNAs were specific to a particular stress (Figure 6, A and B; Supplemental Figure S10A and Supplemental Data Set S9), with the highest proportion associated with temperature stress (cold, heat, or cold + heat). We observed a similar pattern, albeit associated with a larger number of transcripts, for protein-coding genes in both species (Supplemental Figure S10, B and C). As many abiotic stress response genes are regulated by the phytohormone abscisic acid (ABA, Vishwakarma et al., 2017), we next sought to determine which of the Arabidopsis stress-responsive lincRNAs were also ABA responsive. We screened through Arabidopsis RNA-seq data associated with seedlings and roots treated with exogenous ABA (5–100 µM) and identified 672 lincRNAs that were differentially expressed after ABA treatment in these two tissues, 105 of which overlap with our stress-responsive lincRNAs, suggesting these 105 lincRNAs may be stress-responsive in an ABA-dependent manner (Figure 6A, inset).

lincRNAs were predominantly responsive to temperature stress (heat and cold), potentially reflecting global, rather than specific, changes in expression. Thus, we next asked how many lincRNAs, out of the total heat/cold responsive lincRNAs, showed an anti-correlated response to temperature stress (i.e. upregulated in heat and downregulated in cold) and therefore were specific to one stress or another. In both species, heat/cold responsive lincRNAs were predominantly upregulated by heat and repressed by cold (Figure 6, C and D). This pattern was specific for lincRNAs, as a similar number of mRNAs were either upregulated or downregulated under both conditions (Supplemental Figure S10D). Interestingly, several (*n* = 9) of the heat-responsive Arabidopsis lincRNAs were also found to interact with DNA under heat stress (Li et al., 2021; Supplemental Data Sets S2 and S10), suggesting they may function similarly to other DNA-interacting transcriptional regulators, such as ELENA or APOLO. Taken together, we observed that a substantial fraction of lincRNAs were differentially regulated during temperature stress in both Arabidopsis and Brassica. Stress and ABA-responsive lincRNAs for each species are listed in Supplemental Data Set S2. Additionally, all differential expression results from the four focal Brassicaceae can be found in Supplemental Data Set S9.

### Expression of a subset of lincRNAs is correlated with that of adjacent mRNAs

lincRNAs are known to regulate the expression of other genes, either in cis or in trans, through a variety of mechanisms (Kindgren et al., 2019; Gil and Ulitsky, 2020). One signature of cis-regulatory lincRNAs is correlation in expression relative to neighboring genes across a diverse transcriptomic data set. To identify putative cis-regulatory lincRNAs, we searched for correlation between all Arabidopsis and Brassica lincRNAs and their immediate neighboring mRNAs that were expressed at > 0.1 TPM (i.e. both lincRNA and mRNA > 0.1 TPM) in either their respective tissue atlases or heat stress experiments. In the Arabidopsis tissue atlas, we identified 252 lincRNA-mRNA pairs in which both genes in the pair were expressed and for which we could calculate expression correlation. This correlation was significantly more positive than that between mRNA–mRNA pairs or random pairs of genes (Figure 6E, *i*; *P* = 1.62e−14; Wilcoxon rank sum test with Bonferroni multiple testing correction). When examining all genes that fall within 10 kb of an expressed lincRNA, we observe an even stronger positive correlation, in contrast to mRNA–mRNA pairs within the same region, which showed very little correlation across all distances measured (up to 10 kb; Supplemental Figure S11A).

We observed even more lincRNA–mRNA pairs with correlated expression during heat stress in Arabidopsis (*n* = 2,544), again with positive correlation relative to mRNA–mRNA pairs (*P* < 2e−16; Figure 6E, *ii*). We also observed positive expression correlation for Brassica lincRNA–mRNA pairs in the Brassica tissue atlas (3,757 out of 23,756 expressed lincRNAs; Figure 6E, *iii*; Supplemental Figure S11B) and heat experiments (*n* = 6,514; Figure 6E, *iv*), although this correlation was less pronounced than in Arabidopsis. As this correlation may simply reflect increased expression of the mRNA, or general relaxation of open chromatin, we examined the specificity of lincRNA-mRNA correlations by testing to see if expression correlations changed between different experiments. For the 2,435 lincRNA–mRNA pairs whose expression was correlated in the Arabidopsis tissue atlas, 994 pairs were only correlated in the tissue atlas, although one gene from the pair could be expressed in the other experiments (Figure 6, F and G). A further 422 lincRNA–mRNA pairs were correlated in all four data sets, but their correlation coefficient changed across experiments. Indeed, most of the correlated lincRNA–mRNA pairs changed the direction of their correlation between different experiments, suggesting that the mechanisms regulating the adjacent genes are contextually dynamic. In summary, we identify a subset of lincRNAs whose expression appears to be positively correlated with neighboring genes up to at least 10 kb away, suggesting that these lincRNAs might be cis-regulatory RNAs. lincRNA–mRNA pairs with a strong correlation (*r* > 0.5 or *r* < −0.5), as well as all correlated neighboring pairs, are listed in Supplemental Data Sets S2 and S11.

**Figure 7:**
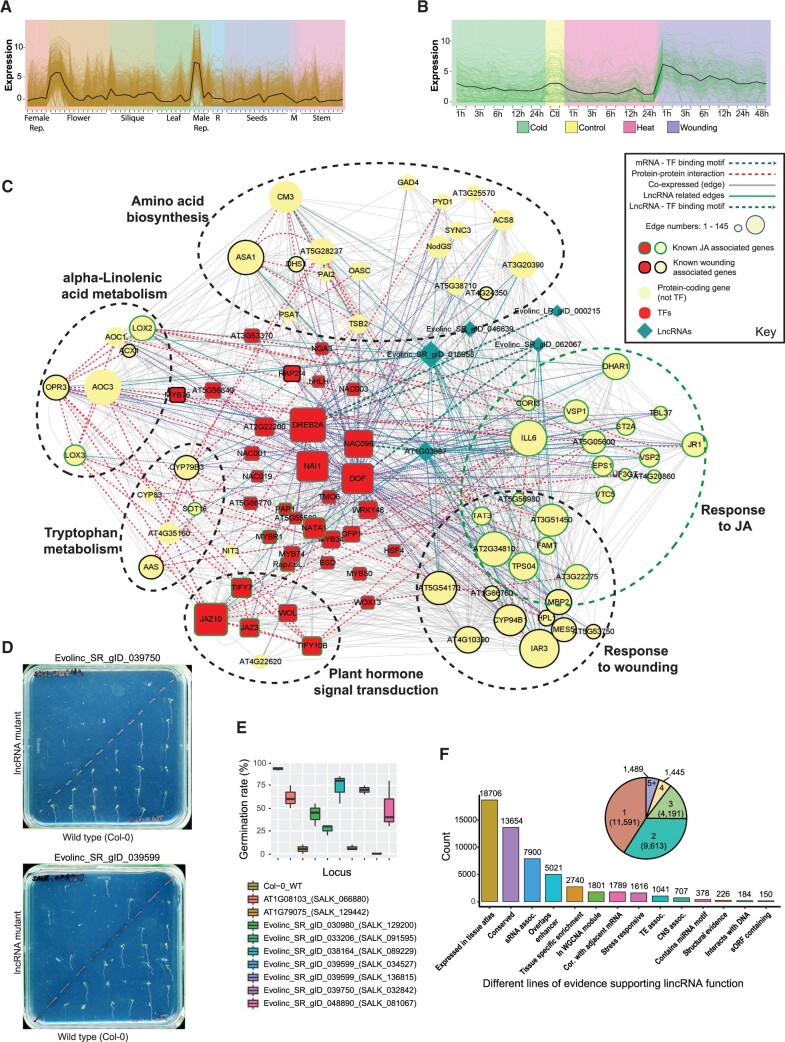
Using guilt-by-association to infer lincRNA function. A, B, WGCNA modules of similarly expressed reproduction-related transcripts from the Arabidopsis Klepikova tissue atlas (A) or wounding-related transcripts from the Klepikova stress experiments (B). Solid black line represents the averaged expression of the module across experiments on the *x*-axis. M, meristem tissue; Male Rep., male reproductive tissues (stamens and anthers). C, Gene network visualization of the early wounding response module in (B). Blue and green arrowed lines represent protein-coding (circles) and lincRNA (teal diamonds) genes containing transcription factor binding motifs for the connected TF (inferred from Arabidopsis DAP-seq data, red boxes). Dashed circles outline clusters of genes enriched for particular KEGG or GO-terms. See key for more details. D, Germination defects/delay in two lincRNA mutants identified in this study (top) relative to wild-type seeds (bottom) 5 days after germination of the wild-type. E, Box plot showing the quantification of germination defects in multiple lincRNA candidates with presumed activities in germination pathways (all mutants display significantly lower germination frequencies relative to the wild-type, *P* < 0.005, Dunnett’s post hoc test). Whiskers represent the upper and lower quartiles, respectively. F, Bar plot depicting the number of lincRNAs for which we have varying degrees of functional evidence. Inset pie-chart depicts the number of lincRNAs with their number of lines of functional evidence.

WGCNAs help to identify clusters of genes that are coordinated in their expression and thus potentially regulated by, or regulate, similar pathways. This allows us to assign putative functions to lincRNAs based on significant co-expression with functionally characterized mRNAs or lincRNAs, a process referred to as guilt-by-association (Tian et al., 2008). To remove noise from normalization across many disparate experiments, we grouped experiments by tissue or, where available, by project (as in the case of the tissue atlases; see Materials and methods). Genes with high median absolute deviation (MAD; top 25%, i.e. genes with a lot of expression variance) were used to generate scale-free networks, from which we only kept genes with strong connectivity (weight score > 0.1). In total, we identified 987 lincRNAs in Arabidopsis and 3,473 lincRNAs in Brassica whose expression profiles were sufficient to classify them into at least one co-expression module. For visualization, we focused on modules with high eigengene variation relative to other modules (Supplemental Figure S12). For example, when we examined the Arabidopsis Klepikova tissue atlas (Klepikova et al., 2016), we identified a module of 710 mRNAs and 43 lincRNAs (22 of which were annotated as part of this study) whose expression peaked in flowers and male reproductive tissues (i.e. anther and pollen; Figure 7A). Within this module, gene ontology (GO) terms associated with fertilization and reproduction were enriched, suggesting that lincRNAs within this module may function during fertilization (Module 3, Klepikova tissue atlas; Supplemental Data Set S12). We also observed lincRNAs that were members of co-expression modules determined from the Klepikova tissue atlas stress experiments (Klepikova et al., 2016). One of these modules contains 209 transcripts (203 mRNAs, six lincRNAs) whose expression peaks rapidly after wounding (Figure 7B). As expected, the GO terms we see with these member mRNAs are highly enriched for response to wounding and jasmonic acid (JA) regulation (Module 6, Klepikova stresses; Supplemental Data Set S12); this phytohormone is released in response to herbivory and biotic stress (Wang et al., 2020).

We visualized the gene network associated with this wounding module using Cytoscape (Shannon et al., 2003), merging publicly available transcription factor binding and protein–protein interaction data (Figure 7C). A large number of transcription factors (TF; red boxes) are associated with either JA signaling or the wounding response in this network. Interestingly, three of the five lincRNAs (teal diamonds) within this module contain TF binding motifs and thus are likely regulated by three highly connected TFs within this network (DREB2A, NAC096, and DOF; dark teal arrowed lines, Figure 6C). We also generated merged networks for ABA-responsive lincRNAs (Supplemental Figure S13), as well as drought and cold responsive lincRNAs (Supplemental Figure S14). We identified a separate module of 140 genes, six of which are lincRNAs, specifically induced under cold stress (Module 7, Klepikova Stresses; Supplemental Data Set S12). Importantly, one of these six lincRNAs was SVALKA, a previously reported cold-induced transcript critical for the freezing response in Arabidopsis. Based on expression patterns and association, we hypothesize that the other five lincRNAs also regulate the Arabidopsis cold-stress response. Thus, through WGCNA and guilt-by-association, we have generated putative annotations for ∼1,000 Arabidopsis and ∼3,000 Brassica lincRNAs that may guide future in vivo functional analyses. These lincRNAs have been annotated with expression modules in Supplemental Data Sets S2 and S12. Detailed WGCNA results can be found in Supplemental Data located at CyVerse.

### Synthesizing our functional assignment approach

To validate our functional assignment approach, we identified a set of candidate seed or germination-associated lincRNAs that were conserved at the sequence level between Arabidopsis and Brassica (node 4, Figure 5A), and for which T-DNA insertion lines were available that would disrupt these lincRNA loci. As these lincRNAs were most highly expressed either late in seed development or early in seed germination, we anticipated phenotypes associated with germination. To test this hypothesis, we screened these lincRNA mutant lines on plates, grown side-by-side with wild-type controls (Figure 7D). We observed varying, but significant (*P* < 0.005, Dunnett’s post hoc test), decreases in germination relative to the controls (Figure 7E). Of the nine insertion lines screened, two were associated with the same lincRNA (Evolinc_SR_gID_03599). While the mechanism associated with the observed germination defect is unclear, we believe that these data both support our strategy for identifying functional lincRNAs and highlight the hundreds to thousands of other potentially functional lincRNAs in these four species.

In summary, we used a wealth of public data, supplemented with short and long-read RNA-seq, to identify and provide putative functional annotations for lincRNAs across four Brassicaceae species. We combined our transcriptomics data with comparative genomic and evolutionary analyses to determine conservation of not just the full-length lincRNAs, but also putative functional elements within them, such as sORFs, structured regions, and miRNA interaction motifs. Using these approaches, we have identified >100,000 Brassicaceae lincRNAs with multiple lines of functional and/or contextual clues that will facilitate downstream functional analyses (Figure 7F).

## Discussion

### A comprehensive and unified lincRNA annotation effort for the mustard lineage

Here, we generated an expansive catalog of HC-lincRNAs for four agricultural and model Brassicaceae species by processing > 20,000 publicly available RNA-seq data sets for those species. We supplemented these publicly available data with our own ONT long-read sequencing data and further annotated the identified lincRNAs with epigenetic, genomic, structural, translational, and evolutionary information. These efforts build on previous efforts to catalog novel transcribed elements within plant genomes (Liu et al., 2012; Moghe et al., 2013) and to provide a more comprehensive lincRNA annotation.

Due to the scale of our efforts and the wealth of data available for these four species, we were able to uncover defining features for Brassicaceae lincRNAs, features that may guide future discovery and annotation efforts in other plant lineages. LincRNAs tend to be mono-exonic, but when multiexonic, harbor longer exons relative to those seen in spliced mRNAs. LincRNAs may be epigenetically regulated in a distinct manner from both protein-coding genes and TEs. Moreover, as expected based on prior observations in plants and mammals, lincRNAs in all four species were, on average, expressed at low levels and displayed significantly higher tissue specificity relative to protein-coding genes in tissue atlases and our ONT data. The exception to this observation is the sORF-containing lincRNAs, which behave more similarly to protein-coding genes in terms of both higher expression levels and tissue specificity. Interestingly, many of the lincRNAs we identified displayed high expression in, or were restricted to, very specific cell types (e.g. meristematic tissue) or experimental conditions (e.g. environmental stress). This observation suggests that (1) lincRNA expression is highly context and cell-type specific, and (2) sampling bulk tissues may not accurately reflect a lincRNA’s contribution to the transcriptome. The lincRNAs restricted to interaccession crosses (as in *B. rapa*) may result from improper transcriptional control given their relatively even distribution across the genome or, albeit less likely, may reflect transcripts that help mediate the compatibility of two subtly different genomes.

### Using comparative genomics to provide functional insights

Given that we identified thousands of lincRNAs in each of our four focal species, functional analyses will need to be prioritized. In order to facilitate that prioritization, we used a comparative genomic approach to assess the degree to which each identified lincRNA is conserved, and whether the detected conservation was driven by specific motifs. As expected based on prior observations in plants and mammals (Nelson et al., 2016), we observed low levels of sequence conservation for lincRNAs identified in each of the four species relative to protein-coding genes. However, when sequence homologs were detected between two species (e.g. Arabidopsis to Brassica), those sequence homologs were predominantly annotated as lincRNAs and not protein-coding genes. Inspired by a smaller comparison between Arabidopsis and *Aethionema*, a representative of the earliest diverging lineage within the mustards (Mohammadin et al., 2015), we also searched for and observed a cohort of lincRNAs that are transcribed from similar genomic positions in multiple species but share little sequence conservation. LincRNAs that regulate gene expression in cis are an interesting class of transcripts from an evolutionary perspective in that positional and transcriptional conservation may be more critical than sequence conservation. Although additional study is needed, we posit that these lincRNAs may play a conserved role in regulating the expression of the orthologous genes to which they are adjacent in each species. An exciting set of candidates for further study are the putative SVALKA loci we identified in Brassica. In Arabidopsis, SVALKA regulates an adjacent, nonoverlapping, protein-coding gene through transcriptional interference. This mode of function in particular might depend more on the conservation of transcription, and from where transcription arises, than it does on sequence similarity.

Identifying lincRNAs in species with recent WGD events (e.g. Camelina and Brassica) allowed us to more closely examine the retention and expression dynamics of lincRNAs following these genomic events. We discovered that lincRNAs are not typically retained as multicopy loci following WGD events, supporting prior results from a smaller set of Arabidopsis-specific lincRNAs (Nelson et al., 2016). In Brassica, lincRNAs are predominantly retained as single copy and are most often located in the least fractionated subgenome. The differential retention of some lincRNAs following fractionation suggests that functional interactions (e.g. genetic or molecular) are preferentially retained following WGD events, which is similar to findings for differentially retained protein-coding genes (Schnable et al., 2012; Emery et al., 2018). When paralogous lincRNAs are retained, their expression appears to be more sensitive to the influence of subgenome dominance than protein-coding genes. However, the retention (and expression) of paralogous lincRNAs such as *HID1* provides the opportunity to explore the evolutionary forces (e.g. neo- or sub-functionalization) that underlie their presence in the genome. Further studies are needed to determine if these paralogous lincRNAs (e.g. *HID1*) have sub or neo-functionalized, as is often the case for retained proteins.

Using multiple sequence alignments for our sets of conserved lincRNAs, we also examined whether the identified structural, putative miRNA binding, or sORFs were within those conserved regions. Although we did identify examples of conserved sORFs, to our surprise, we did not observe strong correlation between sORF and lincRNA conservation. One particularly interesting conserved sORF is found within the lincRNA *AT1G06113*. The Ribo-seq-identified sORF within this lincRNA is only nine amino acids long and is not present in any peptide/protein database, but it is almost perfectly conserved across the Brassicaceae and even in *T. hassleriana* (Cleomaceae). The functional significance of this peptide, as well as the other lincRNA-derived small proteins, remains to be determined. In contrast to the sORF-containing lincRNAs, the regions we identified to be protein-bound and structured were typically conserved to the same degree as the lincRNA itself. This conservation suggests that these structured regions are important for their function and may bind to similar proteins in multiple species. Thus, identifying the protein-binding partner in Arabidopsis might provide functional insights for these lincRNAs across the family and aid in the development of a protein–RNA interaction database for improving functional predictions.

### Using omics approaches to assign putative functions to Brassicaceae lincRNAs

Our ultimate goal, beyond identifying lincRNAs in each of these species, was to annotate these lincRNAs so as to aid in future functional studies. We used expression data to assign lincRNAs into broad regulatory categories, such as stress-responsive, cis-regulatory, or others associated with GO-terms extracted from network analyses. As most functionally described lincRNAs to date are associated with changes in the environment (i.e. biotic/abiotic stress; reviewed in Ariel et al., 2015; Chekanova, 2015), our initial expectations were that most lincRNAs would be stress responsive. Interestingly, this was not the case. Roughly 10% of the lincRNAs identified in Arabidopsis and Brassica are stress-responsive, with most responding to temperature stress. While this could be linked to changes in genome-wide epigenetic control that are not specific to lincRNAs, there does appear to be a degree of response specificity. A majority of the temperature (cold or heat) responsive lincRNAs were either specific to one stress or the other, or showed opposite responses to the two stresses. Furthermore, we also identified a set of lincRNAs whose response appears to be ABA-dependent. The preponderance of lincRNAs associated with temperature stress in our data set may simply reflect sampling bias, as our analyses were dependent on publicly available data. However, given the lincRNAs and NAT-lncRNAs that have already been functionally described as temperature responsive in Arabidopsis (Castaings et al., 2014; Zhao et al., 2018; Kindgren et al., 2019), the potential for widespread adaptation to environmental conditions by lincRNAs remains an exciting avenue for future research.

### Guiding future lincRNA annotation efforts across the plant lineage

Most transcriptomic analyses ignore lincRNAs, not because they are not present in the data, but because existing genome annotations largely lack these transcript classes. Thus, many of the most impactful plant lincRNA functional studies to date have relied on de novo lincRNA classification because the locus of interest was unannotated. Our approach is species-independent, and because we are repurposing available RNA-seq data, naturally focuses on the experimental questions in which the plant community is interested. We aim, and encourage others, to expand these annotation efforts to all plant species with significant RNA-seq data in order to fully understand how lincRNAs contribute to the biology of plants.

## Materials and methods

### Plant materials and growth conditions

*Arabidopsis thaliana* (Col-0; Lamesch et al., 2012); *B.rapa* (R-0-18; Howe et al., 2021), *C.sativa* (cultivar Ames), and *E.salsugineum* (Shandong; Yang et al., 2013) seeds were surface sterilized by washing with 70% ethanol, followed by soaking in 30% bleach and 1% Tween 20 for 10 min before being rinsed and plated on half-strength Murashige & Skoog (MS) medium supplemented with 0.5% sucrose. The plates were placed in the dark at 4°C for 5 days before being moved to a long day (16-h light 22°C/8-h dark 20°C, using T8 fluorescent tube lights providing 150 photons μmol m^−2^ s^−1^) growth chamber. Ten days after germination, seedlings were either collected in liquid nitrogen or transplanted to soil and placed into the same growth chamber. For leaf samples, leaves were either collected 4 weeks after germination, or at the mature most vegetative stage, whichever came first. Finally, for flower samples, open flowers with no sign of developing fruit were collected. All plant samples were immediately frozen in liquid nitrogen and stored in a −80°C freezer until ready for processing.

### Arabidopsis germination assay

All SALK lines were obtained from the Arabidopsis Biological Resource Center (ABRC, abrc.osu.edu). Seeds were sterilized for 10 min in a 50% bleach solution, washed with Milli-Q water, and stored at 4°C overnight in the dark for stratification. Following overnight storage, the seeds were sown on MS agar plates. Finally, the plates were imaged on the first and fifth days of the germination assay using an Epson Scanner.

### RNA extraction and ONT library preparation

Frozen plant samples were pulverized in liquid nitrogen using a chilled mortar and pestle until a fine powder was obtained. RNA was extracted from the samples using an RNeasy Plant Mini kit (Qiagen) following the manufacturer’s instructions. Purified RNA was used as input for the Dynabeads mRNA Purification kit (Invitrogen). Purified poly-A RNA was used as input for the Nanopore direct cDNA sequencing kit (SQK-DCS109) following the manufacturer’s instructions. Nanopore libraries were sequenced on a MINion sequencer (R9.4.1 flowcell). Raw reads were basecalled using a GPU-enabled version of Guppy in the command line.

### Illumina RNA-seq of *C. sativa* seeds

Developing seeds of four *C.sativa* accessions were collected from three independent plants (biological triplicate) at ∼15 days post-anthesis and immediately placed in liquid nitrogen. Total RNA was isolated from developing seeds using a PureLink Plant RNA Reagent (Thermo Fisher Scientific, Waltham, MA, USA) and its associated protocol. Extracted RNA was then purified further using an RNeasy RNA clean-up kit (Qiagen, Valencia, CA, USA) and quantified on a Qubit fluorometer (Life Technologies, Carlsbad, CA, USA). Sequencing libraries were prepared with a SENSE mRNA-seq library prep kit and protocol, using up to 1,000 ng total RNA per sample (Lexogen GmbH, Vienna, Austria). Individual transcriptome libraries were quantified using a Qubit fluorometer, and fragment size, distribution, and overall library quality were determined with an Agilent Bioanalyzer (Agilent, Santa Clara, CA, USA) system. Samples were pooled into three final libraries and sequenced by Novogene (Sacramento, CA, USA) on an Illumina HiSeq platform (Illumina, San Diego, CA, USA), producing 150-bp paired-end reads.

### lincRNA identification and basic characterization

The RMTA version 2.6.3 (Peri et al., 2019) pipeline was used to process all available short-read RNA-seq data as of December 2018 within the CyVerse Discovery Environment (Merchant et al., 2016) using the HiSat2 v2.1.0 (Kim et al., 2019) and Stringtie v1.3.4 (Pertea et al., 2016) mapping and assembly options. Assembled transcripts were then processed through the Evolinc version 1.6 (Nelson et al., 2017) pipeline to identify lincRNAs. The following databases and genome versions were used as a reference for the initial RMTA workflow (including mapping, quantification, and transcript assembly): for *A.thaliana*, the TAIR-10 assembly; for *B.rapa*, Ensembl v1.0; for *C.sativa*, Ensembl v2.0 (Plant Release 51); and for *E.salsugineum*, Phytozome v1.0 (Yang et al., 2013). An updated annotation including newly identified lincRNAs for each species can be downloaded from the CyVerse Data Store.

Basecalled Nanopore reads were demultiplexed and processed following (Eccles, 2019). To identify lincRNAs with Evolinc, processed reads were aligned to each species’ genome using Minimap2 version 2.17 with the -ax splice argument (Li, 2018). Mapped reads were assembled into transcripts using Stringtie2 (Kovaka et al., 2019) using the -L parameter. Transcript assemblies were then used as input for Evolinc for lincRNA identification.

The BEDTools suite version 2.26.0 (Quinlan and Hall, 2010), nuc function was used to characterize the GC content and gene lengths of mRNAs and lincRNAs. Exon counts were determined using the R Core Team (2013, version 4.1.0) package GenomicFeatures version 1.44.1 (Lawrence et al., 2013).

### Analysis of DNA methylation patterns and histone modification dynamics

lincRNA and mRNA epigenetic profiles were monitored by reprocessing publicly available whole-genome bisulfite sequencing (WGBS) datasets as well as chromatin immunoprecipitation with sequencing (ChIP-seq) experiments (see Supplemental Data Set S6). WGBS data were processed through using the methylpy analysis pipeline version 1.2.9 (Schultz et al., 2016). Fastq files were first trimmed using Trim Galore version 0.6.6 (Krueger, 2018, https://github.com/FelixKrueger/TrimGalore) with default settings. Trimmed fastq reads were then run through the methylpy single-end pipeline using default settings, except for specifying the remove-clonal option. Methylpy was then used to convert the output of the single-end pipeline to bigWig format in 100 nucleotide bin-sizes. ChIP-seq fastq files were trimmed in the same manner as the WGBS fastq files above. Trimmed fastq files were mapped to the Arabidopsis or Eutrema genome using bowtie2 version 2.3.4.1 (Langmead and Salzberg, 2012) using default settings. SAM files from bowtie2 were converted to sorted BAM files using samtools version 1.7 (Li et al., 2009). bigWig files from WGBS data and sorted bam files from ChIP-seq data were used as input to deepTools version 3.5.1 (Ramírez et al., 2014). Sorted bam files were converted to bigWig using the deepTools bamCoverage function, setting the bin-size to 10 nucleotides, normalization using counts per million, and the ignore duplicates argument. Metaplots and heatmaps were produced using the computeMatrix function within deepTools using the scale-regions option with the computeMatrix function and scale-regions option. The plotHeatmap function was used to generate the plots, with the kmeans option specified when epigenetic clusters are shown.

### Identifying small RNA-associated lincRNAs

Multiple independent approaches were taken to identify small RNA-generating loci, including approaches to identify miRNA precursors and siRNA precursors. The Arabidopsis Araport11 annotation identified more than 35,000 small RNA-producing loci (Cheng et al., 2017). This Araport11 small RNA annotation was compared to the lincRNA annotation generated in this study using bedtools intersect version 2.26.0 (Quinlan and Hall, 2010) with a minimum overlap of one base and no consideration for strand of the features. Only lincRNAs overlapping small RNA features of 21–24 nucleotides were considered to overlap small RNAs.

To identify RdDM-dependent lincRNAs, small RNA-seq (sRNA-seq) data sets from (Zhou et al., 2022, specifically wild-type and pol-iv mutant data, see Supplemental Table 6) were re-processed using the R package DEUS version 1.0 (Jeske et al., 2019). Raw fastq files were trimmed using Trim Galore as described above and then used as input for the DEUS workflow, following the DEUS vignette for sequence counting, differential expression analysis, and running BLAST. Any differentially expressed (pol-iv mutant vs. wild-type) small RNA cluster that was 21–24 bases in length and mapped to a lincRNA sequence was considered to be a putative RdDM-associated lincRNA.

Finally, lincRNAs identified in this study that were physically associated with POL-V through RIP-seq (Böhmdorfer et al., 2016) were identified. Raw RIP-seq reads were mapped to the Arabidopsis TAIR 10 genome using STAR version 2.7.10a (Dobin et al., 2013). A genome index was generated using the TAIR 10 genome fasta file, as well as the lincRNA gff3 file generated in this study with the additional parameters sjdbOverhang set to 49 and genomeSAindexNbases set to 12. For read mapping, the maximum intron length was set to 50,000 and outFilterMismatchNmax was set to 5. Sorted BAM files were quantified with featureCounts version 2.0.3 (Liao et al., 2014). Differentially expressed (POL-V bound) lincRNAs were then identified with the R package DESeq2 version 1.32.0 (Love et al., 2014) using an adjusted *P*-value cutoff of 0.05 and log2 fold change greater than 1 or less than −1.

### Annotation of lincRNAs for TE composition and overlap using Rfam library

To identify lincRNAs that may act as small RNA precursors or are misannotated TEs, two approaches were taken. First, the Extensive de-novo TE Annotator (EDTA) pipeline version 2.0.0 (Ou et al., 2019) was run using default parameters for each focal species. The genome fasta file of each species was used as input for EDTA, as well as a fasta file of annotated mRNA coding sequences. Of the two output files, only structurally intact TEs were retained and used as input for Bedtools to intersect lincRNA and TE coordinates. LincRNAs overlapping at least 50% of these newly annotated and intact TEs were annotated as TE-associated. To identify overlap between lincRNAs and the Rfam library, each of the four focal Brassicaceae genomes was scanned using Infernal version 1.1.2 (Nawrocki and Eddy, 2013) against the Rfam library of noncoding RNAs (current Rfam library as of December 1, 2021; Kalvari et al., 2021) following the Rfam documentation for Genome annotation at https://docs.rfam.org/en/latest/genome-annotation.html. As with the TEs above, Rfam annotated noncoding RNA features needed to overlap at least 50% of the lincRNA feature in order for the lincRNA to be annotated as overlapping an Rfam feature.

### Characterization of lincRNA expression patterns

To characterize expression patterns from ONT-seq data, Minimap2 was used to map ONT-reads to transcriptomes for each of the respective species’ updated gene sets (prior annotated genes + Evolinc lincRNAs) using the following parameters: -a -x map-ont -N 100 -p 0.99. BAM files generated by Minimap2 were used as input for Salmon version 1.5.1 in alignment-based mode, specifying the –noErrorModel option (Patro et al., 2017). TPM values were aggregated from each experiment using the tximport R package version 1.20.0 (Soneson et al., 2015) to obtain gene-level expression estimates.

Specific Illumina short-read data sets from Arabidopsis and Brassica were used to gain additional resolution of tissue-specific expression. For Arabidopsis, the Klepikova et al.’s (2016) (NCBI PRJNA314076) tissue expression atlas was reprocessed and for Brassica, two data sets were combined to create a tissue atlas similar to Arabidopsis (PRJNA253868 and PRJNA185152). To quantify expression from these data sets, decoy aware transcriptome indices were generated for Salmon. Raw RNA-seq reads (fastq) associated with each data set were quantified using Salmon with the validated mappings and gcBias parameters selected for all experiments. Gene level expression values were obtained as above using tximport. To calculate the tissue specificity metric τ (TAU), TPM values were first averaged across replicates. TAU was then calculated as described by Yanai et al. (2005) using quantile-normalized TPM values generated from the preprocess Core R package version 1.54.0 (Bolstad, 2013). To assess the tissue with maximum expression, variance-stabilized transformed expression values generated from DESeq2 were utilized (vst).

All differential expression analyses were performed using the DESeq2 package. Complex design formulae were not used in building DESeq objects (i.e. only a single interaction term was used). For RNA-seq data sets containing multiple experimental variables, pairwise differential expression analyses were performed using the contrast argument in the results function. For time-course studies, only the first and last treatments were examined, treating each of them as separate analyses, unless otherwise noted. Genes were considered to be differentially expressed if they had a log_2_ fold change greater or less than 1 or −1, respectively, as well as an adjusted *P*-value (*q*-value, false discovery rate) of 0.05 or lower.

### Analysis of co-expression modules

Weighted gene co-expression networks were constructed to cluster genes with similar expression patterns and to identify groups of genes related to stress responses using the WGCNA R package (Langfelder and Horvath, 2008). For each data set, genes with a high median absolute deviation (MAD) score (Top 25%) were retained for analysis. Initially, we determined the lowest soft power threshold to flatten the index curve in order to reach a high value to satisfy a scale-free network topology (> 0.8; Zhu et al., 2018). Block-wise modules were then constructed with this lowest soft power along with other parameter settings (maxBlockSize = 10,000, TOMType = unsigned, minModuleSize = 50, reassignThreshold = 0, mergeCutHeight = 0.1, deepSplit = 2). Based on groups of genes merged by the network (known as modules), we calculated the eigengene value per module, representing the first component of gene expression. This value is an indicator used to determine the relatedness of modules to the properties of samples. In particular, modules bearing extreme eigengene values from stress-treated samples were proposed to be potentially involved in stress responses. Finally, genes and lncRNAs with strong connectivity (weight score > 0.1) from each module were filtered. Modules associated with extreme eigengene values of stress-treated samples were identified as key modules for downstream regulatory network analysis.

### Regulatory network analysis of modules

To further investigate molecular interactions among stress response regulators and lncRNAs on top of their co-expression patterns, we incorporated regulatory information, including TF-binding interactions and protein–protein interactions (PPIs), into co-expression networks. We started with functional analysis of co-expression modules by performing GO and KEGG pathway enrichment test using clusterProfiler and biomaRt R package (Durinck et al., 2005; Yu et al., 2012; cutoff: *P* < 0.001, background annotation= athaliana_eg_gene) to test if stress response biological processes or pathways terms were enriched in particular networks. For those networks with stress-related functional terms, we obtained TFs and protein kinases based on information from TAIR and annotated unknown proteins using the iTAK TF and kinase classification tools (Zheng et al., 2016). For these non-TF protein-coding genes and lncRNAs, we performed motif enrichment of their respective 1-kb upstream sequences to retain HC TF–target interactions using AME software (McLeay and Bailey, 2010; parameter: –scoring avg –method fisher –hit-lo-fraction 0.25 –evalue –kmer report-threshold 10.0, cutoff: TP values > 3) using the Arabidopsis DAPseq database as the background library (O'Malley et al., 2016). In addition, PPIs among genes within the same module were explored by matching their protein sequences against the STRING database (Szklarczyk et al., 2017; confidence level: > 0.7). Finally, we merged co-expression, PPI, and TF-binding networks using the Cytoscape union function for further regulatory mechanisms analysis (Shannon et al., 2003).

### Measuring expression correlation of adjacent genes

Arabidopsis and Brassica expression data from the above-described tissue atlases or heat-treatment experiments (Arabidopsis: PRJNA324514, Brassica: PRJNA298459) were initially used to assess neighboring gene expression correlations. Gene expression data sets were first variance stabilized in DESeq2, and genes with low expression variance were then removed as described above. Pearson correlation coefficients of expression were then calculated between all remaining genes post-filtering using the corrr R package version 0.4.3 (Kuhn and Wickham, 2020). Relevant correlations were then filtered for lincRNAs and their nearest upstream and/or downstream mRNA neighbors. lincRNA–mRNA pairs separated by fewer than 100 bp were removed before subsequent analyses. Random gene pairs were generated from all pairwise correlations using the slice_sample function from the dplyr R package version 1.0.7 (Wickham et al., 2015).

To analyze all gene pairs within defined distances, the bedmap function from the BEDOPS suite version 2.4.38 (Neph et al., 2012) was used with the range, echo, and echo-map-id options. This generated all lincRNA–mRNA or mRNA–mRNA pairs within 200, 500, 1,000, 2,000, 5,000, and 10,000 bp of each other. These gene pairs were used to filter for the pairwise correlations generated above. Correlation values at each distance were then analyzed and plotted. To determine whether neighboring correlations were consistent or dynamic between experiments, additional Arabidopsis experiments were incorporated and evaluated (PRJNA324514, PRJNA319318, and PRJNA506408, see Supplemental Data Set S8). These gene expression data sets were processed in the same way as the tissue atlas and heat treatment experiments described above. The ComplexUpset R package version 1.3.3 (Krassowski et al., 2021) was used to visualize and process which lincRNA-adjacent mRNA pairs were shared or distinct between experiments.

### Identifying translated sORFs from Ribo-seq

Translated sORFs within the lincRNAs were identified using our recent Ribo-seq and RNA-seq data from Arabidopsis seedlings (GEO accession no. GSE183264; (Wu and Hsu, 2021). Briefly, BAM files of the Ribo-seq and RNA-seq data and a GTF containing the lincRNAs and Araport11 annotated genes were imported into RiboTaper (Calviello et al., 2016). The Ribo-seq read lengths and offsets for RiboTaper were 24, 25, 26, 27, 28 and 8, 9, 10, 11, 12, respectively, as previously described (Wu and Hsu, 2021). RiboTaper then computed 3-nucleotide periodicity, which corresponds to translating ribosomes moving three nucleotides per codon, in each possible ORF within the transcripts. The sORFs were considered translated if they displayed significant 3-nucleotide periodicity, and the translated ones were extracted from the RiboTaper output ORF_max_filt file.

To identify lincRNAs harboring putative sORFs based on mass spectrometry data, data from proteomic experiments PXD026713 and PXD009714 were retrieved from the PRIDE repository. Raw chromatograms were analyzed using MaxQuant software (Version 1.6.0.16) with Andromeda- an integrated peptide search engine (Cox et al., 2011). The following search settings were applied: a maximum of two missed cleavages was allowed, and the threshold for peptide validation was set to 0.01 using a decoy database. In addition, methionine oxidation and N-terminal acetylation were considered variable modifications, while cysteine carbamidomethylation was a fixed modification. The minimum length of a peptide was set to at least seven amino acids. Moreover, label-free protein quantification was applied. Peptides were identified using the Araport 11 database (The Arabidopsis Information Resource, www.Arabidopsis.org) and a library of all Arabidopsis lincRNA ORFs (positive strand) obtained using Transdecoder. To identify overlap between these sORFs and known protein domains, amino acid sequences were used as queries in BLASTp searches using adjusted parameters (default NCBI parameters) for short peptide sequences.

### Evolutionary analyses

lincRNA sequence homologs were identified using the Evolinc-II module (v2.0, https://github.com/Evolinc/Evolinc-II; e-value of −10), with the following genomes: *Arabidopsis thaliana* (TAIR10, Lamesch et al., 2012), *Arabidopsis lyrata* (Ensembl v1.0, Hu et al., 2011), *Capsella grandiflora* (Phytozome v1.1, Slotte et al., 2013), *Capsella rubella* (Phytozome v1.1, Slotte et al., 2013), *Camelina sativa* (Ensembl v2.0), *Cardamine hirsuta* (v1.0, Gan et al., 2016), *Brassica rapa* (Ensembl v1, Wang et al., 2011; Cheng et al., 2013a), *Schrenkiella parvula* (Phytozome v2.0, Dassanayake et al., 2011; Oh et al., 2014), *Eutrema salsugineum* (Phytozome v1.0, Yang et al., 2013), *Aethionema arabicum* (CoGe vVEGI 2.5. gID 20243; Haudry et al., 2013; Nguyen et al., 2019), *Tarenaya hassleriana* (CoGe v4, gID 20317, Cheng et al., 2013b). For each of the four species, the entire lincRNA list (LC + HC) was included as a query in the analyses. LincRNAs were determined to be restricted to a particular node if no sequence homolog was identified in a more distantly related species. LincRNAs were determined to be conserved as lincRNAs or mRNAs in other species if they overlapped by 50% or more with an annotated gene on the same strand. LincRNAs were determined to overlap with one of the Arabidopsis CNS (Haudry et al., 2013) if they overlapped with 50% or more of the CNS using the Bedtools intersect tool. If not, they were considered to be unannotated. Multiple sequence alignments produced by Evolinc-II (using MAFFT) were imported into Geneious (Genious Prime 2021.1.1, https://www.geneious.com) for downstream structure, sORF, and miRNA motif analysis.

Syntenic but sequence divergent lincRNAs were identified by downloading the DAGChainer output, with genomic coordinates, from pairwise CoGe SynMap (Haug-Baltzell et al., 2017; Nelson et al., 2018) analyses between Arabidopsis and each of the three other species (links to regenerate analyses: Camelina https://genomevolution.org/r/1fjg7, Eutrema https://genomevolution.org/r/1f7si, and Brassica https://genomevolution.org/r/1f79g). LincRNAs that were found within syntenic blocks (10 colinear protein-coding genes), between orthologous genes in either of the pairwise SynMap analyses, and in the same orientation to at least one of the neighboring orthologous genes were considered to be SBSD lincRNA loci. To identify lincRNAs with reduced sequence homology, the 5′ or 3′ 200 nts of each SBSD lincRNA from Camelina, Brassica, or Eutrema, as well as 200 nts upstream of the transcription start site (i.e. promoter region) were used separately as queries in a reciprocal best BLASTn approach (e-value = 1e^−^^10^) against the Arabidopsis genome. Bedtools intersect was used to determine if BLAST hits corresponded to the appropriate syntenic location.

To infer lincRNA gene family contraction or expansion, a rudimentary ancestral state reconstruction was performed. For Arabidopsis, ancestral gene copy number for each Arabidopsis lincRNA was inferred by averaging the number of recovered sequence homologs in (at minimum) *A. lyrata*, *C. rubella*, and *C. grandiflora*. Species-specific lincRNAs were not examined. For Camelina, *C. rubella*, *C. grandiflora*, *A. thaliana*, and *A. lyrata* were used to determine the copy number in the last common ancestor. This value was then multiplied by three (to account for the Camelina-specific whole-genome triplication event). Values above or below this value were considered to be expansions or contractions, respectively. A similar approach was performed for Brassica and Eutrema.

MSAs were manually scanned to infer the depth of conservation of sORFs, putative miRNA binding motifs, and structural/protein-binding elements. On top of lincRNA sequence homology and synteny requirements, for an sORF to be considered conserved, the start and stop sites within the annotated Arabidopsis lincRNA must be positionally conserved (within ± three AA). In addition, the translated amino acid sequence must be 75% identical in pairwise alignments between Arabidopsis and each putative homologous sORF. To identify putative miRNA binding sites, all lincRNAs were scanned for motifs using psRNATarget (Dai et al., 2018) using an expectation score of 2.5 as cutoff. LincRNAs with putative miRNA binding motifs were then compared against the list of lincRNAs that were conserved outside of Arabidopsis. MSAs were then scanned for the presence of miRNA motifs. Motifs with complete coverage and no more than two (pairwise) mismatches in at least one other species were considered for evolutionary comparisons. For conservation of structural/protein-binding motifs, structured regions inferred by PIP-seq (GEO accession numbers GSE58974 and GSE86459; Gosai et al., 2015; Foley et al., 2017) were intersected with lincRNAs using Bedtools intersect (Quinlan and Hall, 2010). Arabidopsis lincRNAs, their sequence homologs (from Evolinc-II) and structured regions were combined into an MSA using MAFFT (Nakamura et al., 2018) for manual inspection. PIP-seq motifs were considered conserved if the entire motif was contained within an alignable region of a sequence homolog from another species. For a motif (sORF, miRNA, or structural) to be considered conserved to a particular node, at least one species that shares that node with Arabidopsis was required to contain those motifs under the parameters described above.

## Accession numbers

Supplemental Data sets can be found at https://de.cyverse.org/data/ds/iplant/home/andrewnelson/Palos_Brassicaceae_lncRNAs_May_2022?type=folder&resourceId=c90e8878-a0b1-11ec-97f4-90e2ba675364.

Mapped read files for all Brassicaceae can be found at https://de.cyverse.org/data/ds/iplant/home/andrewnelson/BAM_files_for_public?type=folder&resourceId=91737c4a-574a-11eb-93a8-90e2ba675364.

A Jupyter notebook binder for visualizing expression data can be found at https://github.com/Evolinc/Brassicaceae_lincRNAs.

## Supplemental data

The following materials are available in the online version of this article.

**Supplemental Figure S1.** Assessing the assembly quality of Arabidopsis lincRNAs.

**Supplemental Figure S2.** Comparing sORF length with lincRNA length.

**Supplemental Figure S3.** Additional basic characterization of Brassicaceae lincRNAs.

**Supplemental Figure S4.** Additional expression characteristics.

**Supplemental Figure S5.** Example screenshot of Clustergrammer Jupyter notebook in which users can examine normalized expression values for mRNAs and lincRNAs across multiple stress and tissue atlases.

**Supplemental Figure S6.** Evolutionary features of Brassicaceae lincRNAs.

**Supplemental Figure S7.** Evolution of functionally characterized lincRNAs.

**Supplemental Figure S8.** Subgenome expression dominance of Brassicaceae lincRNAs.

**Supplemental Figure S9.** Examples of deeply conserved lincRNA motifs.

**Supplemental Figure S10.** Differential expression during stress.

**Supplemental Figure S11.** Gene expression correlation (Pearson) between lincRNA-mRNA and mRNA–mRNA pairs within defined distances in Arabidopsis and Brassica tissue atlases.

**Supplemental Figure S12.** Assessment of parameters used to generate co-expression networks from the Klepikova stress data set.

**Supplemental Figure S13:** Gene network visualization of an ABA-responsive module.

**Supplemental Figure S14.** Gene network visualization of a drought and cold-responsive module.

**Supplemental Data Set S1.** List of SRAs examined for all four species.

**Supplemental Data Set S2.** Functional annotations for each lincRNA from all four species.

**Supplemental Data Set S3.** Araport11 lncRNAs that were removed from analysis.

**Supplemental Data Set S4.** sORF and structural motif conservation and characteristics.

**Supplemental Data Set S5.** Predicted miRNA binding motifs and enhancer overlaps.

**Supplemental Data Set S6.** Overlap of lncRNAs with TEs and rFam RNAs.

**Supplemental Data Set S7.** Evolinc II results for all four species and CNS overlap.

**Supplemental Data Set S8.** SRAs, with associated metadata, used in targeted transcriptomic studies.

**Supplemental Data Set S9.** Differential expression results from all species.

**Supplemental Data Set S10.** Chromatin-bound RNAs in seedlings and under heat stress.

**Supplemental Data Set S11.** LincRNA and adjacent mRNA expression correlation.

**Supplemental Data Set S12.** WGCNA information for Arabidopsis and Brassica.

## Supplementary Material

koac166_Supplementary_Data

## Notes

K.P., E.L., M.A.B., and A.D.L.N. developed the project. K.P., L.Y., L.W., E.S., and A.D.L.N. performed the analyses. A.C.N.D. performed RNA extractions and ONT-sequencing. C.E.R. performed Arabidopsis lincRNA mutant germination assays. J.R.B. performed the Camelina RNA-seq. H.-Y.L.W. and P.Y.H. performed the sORF identification from Ribo-seq data. B.D.G. analyzed the PIP-seq data. E.S. and A.S. examined the MS data. K.P., L.Y., B.D.G., E.L., M.A.B., and A.D.L.N. wrote the article.

The author responsible for distribution of materials integral to the findings presented in this article in accordance with the policy described in the Instructions for Authors (https://academic.oup.com/plcell) is: Andrew D.L. Nelson (an425@cornell.edu).
