# Supplementary material for: Identification and functional annotation of long intergenic non-coding RNAs in Brassicaceae
Source: Plant Cell. 2022 Jun 6;34(9):3233–60. doi: 10.1093/plcell/koac166 (PMC9421480; doi:10.1093/plcell/koac166)
Supplement: koac166_Supplementary_Data [file koac166_supplementary_data.zip › Supplemental_Figures.pdf]

Supplemental Figure 1

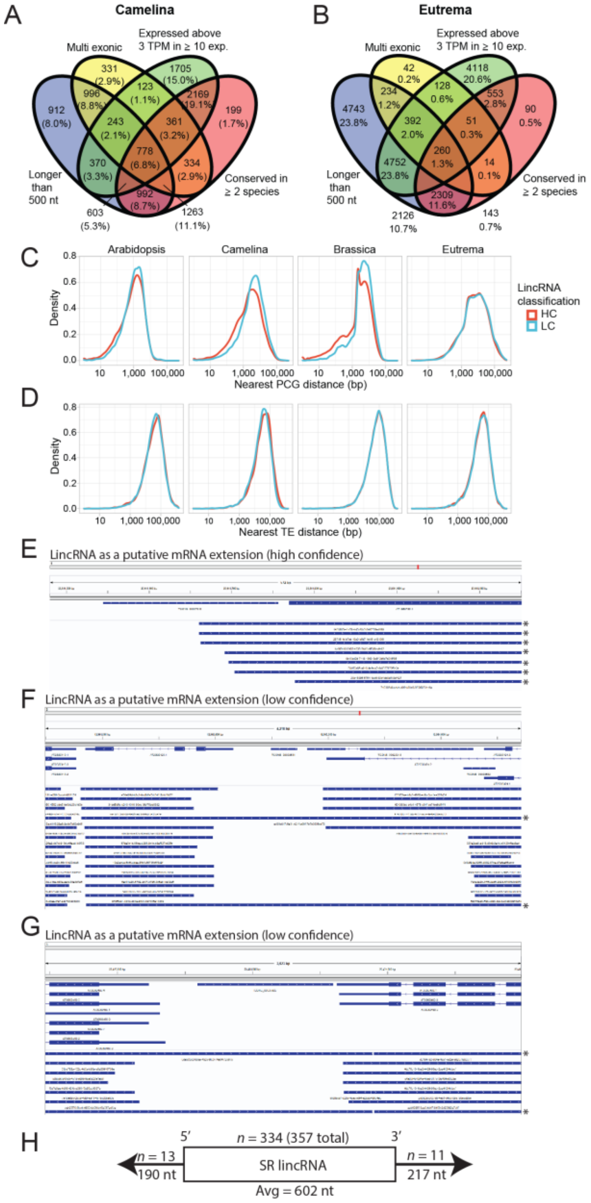

Supplemental Figure 1: Supports Figure 1. Assessing the assembly quality of Arabidopsis lincRNAs. **A-B**) Venn diagram of overlap in the Illumina HC group of Camelina (**A**) and Eutrema (**B**) lincRNAs using different hierarchical filters. **C-D**) Density plot comparing the proximity of HC and LC lincRNAs to the nearest protein-coding genes (PCG; **C**), and transposable elements (TE; **D**). Differences between HC and LC lincRNAs were not significant based on a Student's t-test with multiple testing adjusted using Bonferroni correction. **E**) Illumina short read RNA-seq lincRNA (TCONS_00007019) which was reassessed as an UTR extension of a neighboring mRNA based on Nanopore long read sequencing. **F-G**) Two different lincRNAs initially believed to be mRNA associated, but upon closer inspection were miscalled due to apparent genomic DNA contamination in the ONT-sequencing data (TCONS_00028531 in (**F**) and TCONS_00037603 in (**G**). **H**) Comparing the annotated gene structure of lincRNAs assembled in both long and short sequencing reads (n = 357). 334 of the lincRNAs assembled in both technologies were in complete agreement regarding 5' and 3' positions, as well as exon structure. 13 lincRNAs were annotated as being, on average, 190 nt longer in the 5' directions by ONT-sequencing, whereas 11 were annotated as being 217 nt longer in the 3' direction.

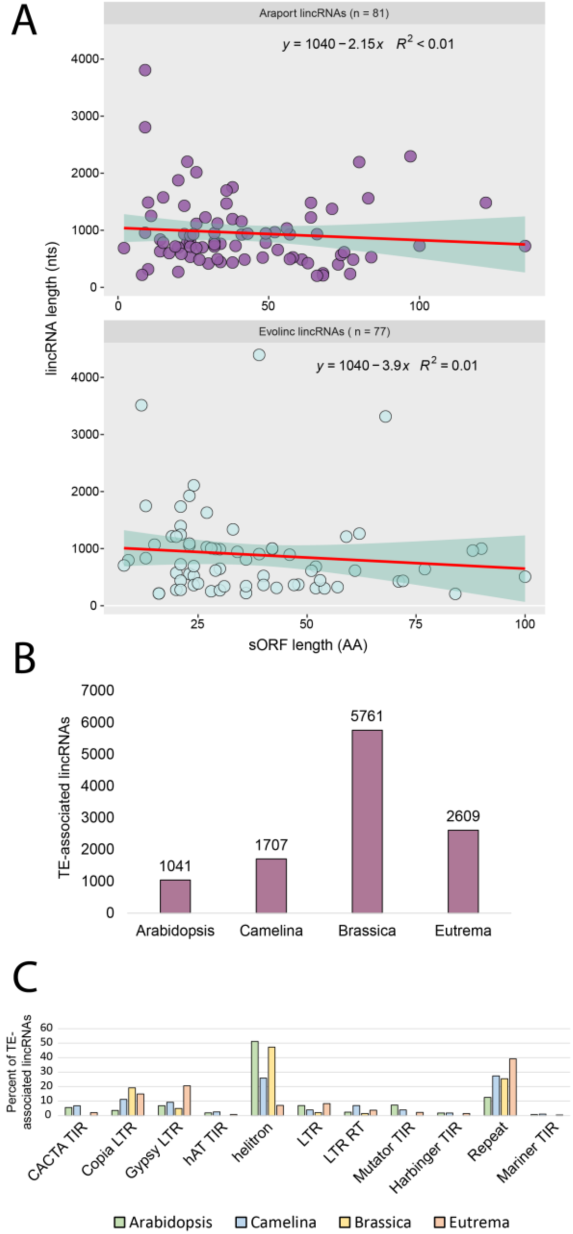

Supplemental Figure 2: Supports Figure 2. Comparing sORF length with lincRNA length. **A)** Scatterplots describing the lack of correlation between sORF length and lincRNA transcript length for Araport (top) lincRNAs and Evolinc (bottom) lincRNAs. **B)** Barplot describing the number of TE associated lincRNAs in each species. Y-axis is the total number of TE-associated lincRNAs. **C)** Barplot describing the percentage of TE-associated lincRNAs that contain each class of TEs in each species.

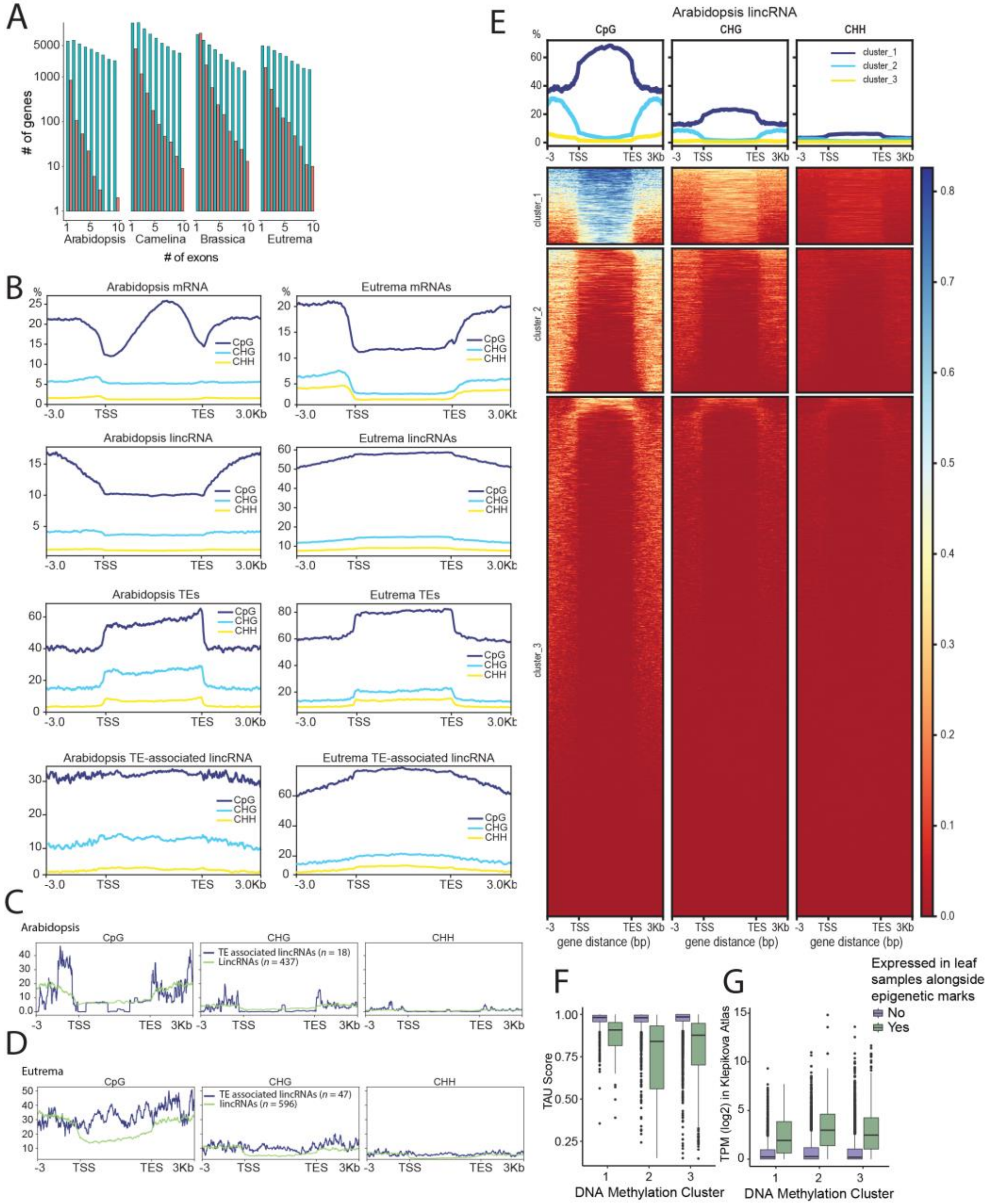

Supplemental Figure 3: Supports Figure 3. Additional basic characterization of Brassicaceae lincRNAs. **A**) Exon per transcript distribution of lincRNAs and mRNAs in each of the 4 focal species (mRNAs in blue and lincRNAs in red.) **B**) Metagene plots of CpG, CHG, and CHH DNA methylation in different gene types of Arabidopsis and Eutrema. The y-axis represents the weighted average DNA methylation levels over 100 bp bins (ie., percent methylation). **C-D**) Metagene plots of CpG, CHG, and CHH DNA methylation for TE-associated versus all lincRNAs expressed in Arabidopsis (**C**) and Eutrema (**D**) leaf tissue . **E**) Hierarchical clustering of CpG, CHG, and CHH methylation patterns in all Arabidopsis lincRNAs regardless of expression. **F**) Box and whiskers plot of TAU scores for lincRNAs that fall into the different epigenetic clusters from (**E**), separated based on whether or not they are expressed in the leaf samples from which the epigenetic data were obtained. **G**) Box and whiskers plot of expression (length-normalized TPM) for lincRNAs that fall into the different epigenetic clusters in (**E)**. Bins are similar as in (**F**).

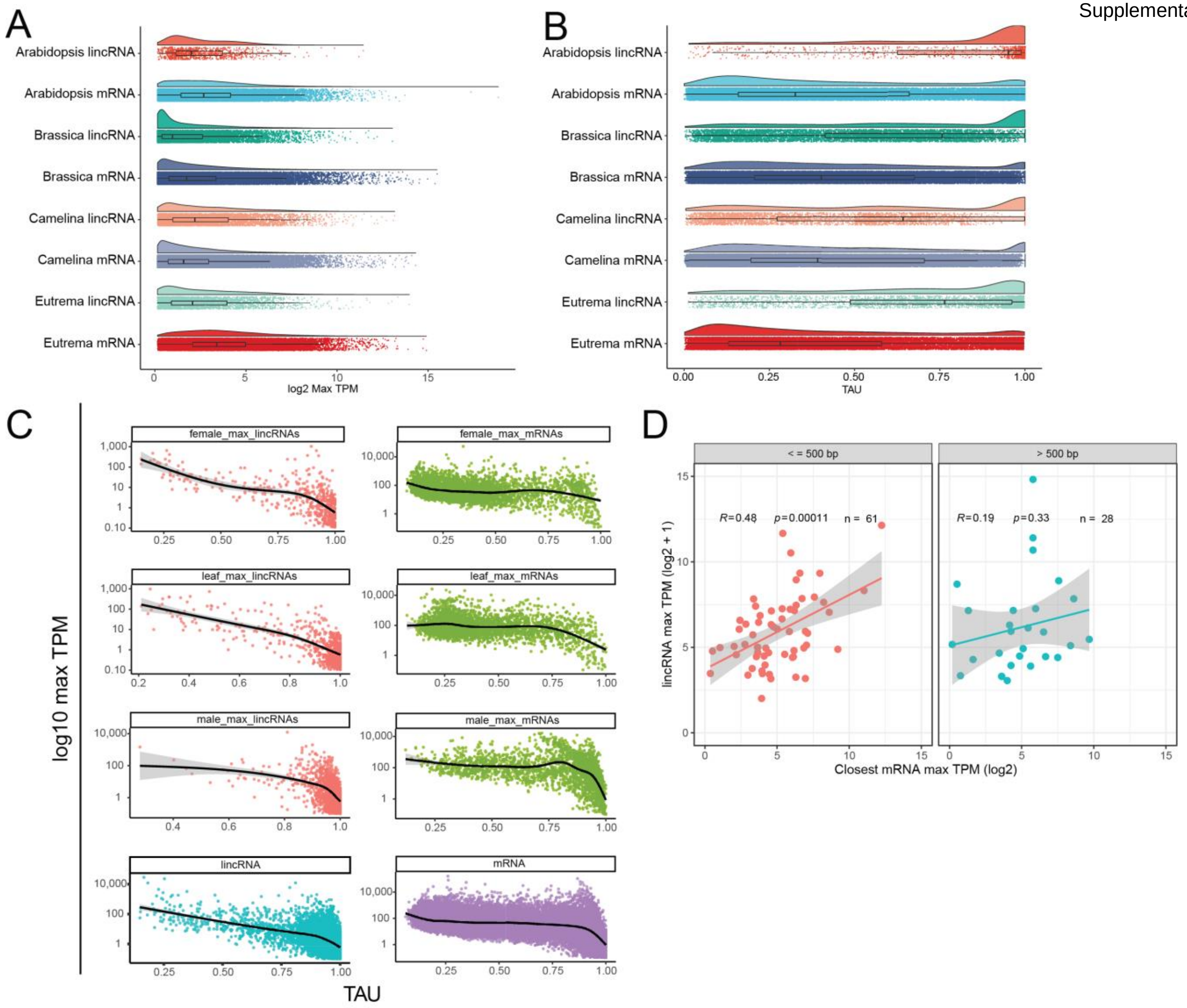

Supplemental Figure 4: Supports Figure 4. Additional expression characteristics. **A**) ONT-sequencing derived maximum TPM values for lincRNAs and mRNAs for each focal species. Each species' mRNA-lincRNA comparison is significantly different at $P < 1.0e\text{-}9$ using a pairwise Wilcoxon rank sum test with Bonferroni multiple testing correction. **B**) Tissue specificity (TAU) comparisons from Nanopore TPM values for mRNAs and lincRNAs from all four species. Each species' mRNA-lincRNA comparison is significantly different at $P < 2e\text{-}16$ using a pairwise Wilcoxon rank sum test with Bonferroni multiple testing correction. **C**) Relationship between maximum expression (TPM) and tissue specificity (TAU) between all expressed Arabidopsis lincRNAs (left) and mRNAs (right) within female reproductive tissues (top) leaf tissue, male reproductive tissue, and all tissues combined (bottom). Black lines represent the best fit line of the data. **D**) Relationship between expression levels of low TAU (broadly expressed) lincRNAs and their neighboring mRNAs divided into two groupings based on distance to closest mRNA.

Visualize the gene (lncRNAs) Expression Data based on Z-score

In [9]:
```
n2 = Network(CGM2)
n2.load_df(df['expression'], meta_col=df['meta_data'], col_cats=col_cats)
n2.set_global_cat_colors(df_colors)
n2.filter_N_top(axis='row', N_top=num_rows, rank_type='var')
n2.normalize(axis='row', norm_type='zscore', z_clip=5)
n2.set_manual_category(col='Custom', preferred_cats=df_colors)
n2.widget(link_net_js=n1)
```

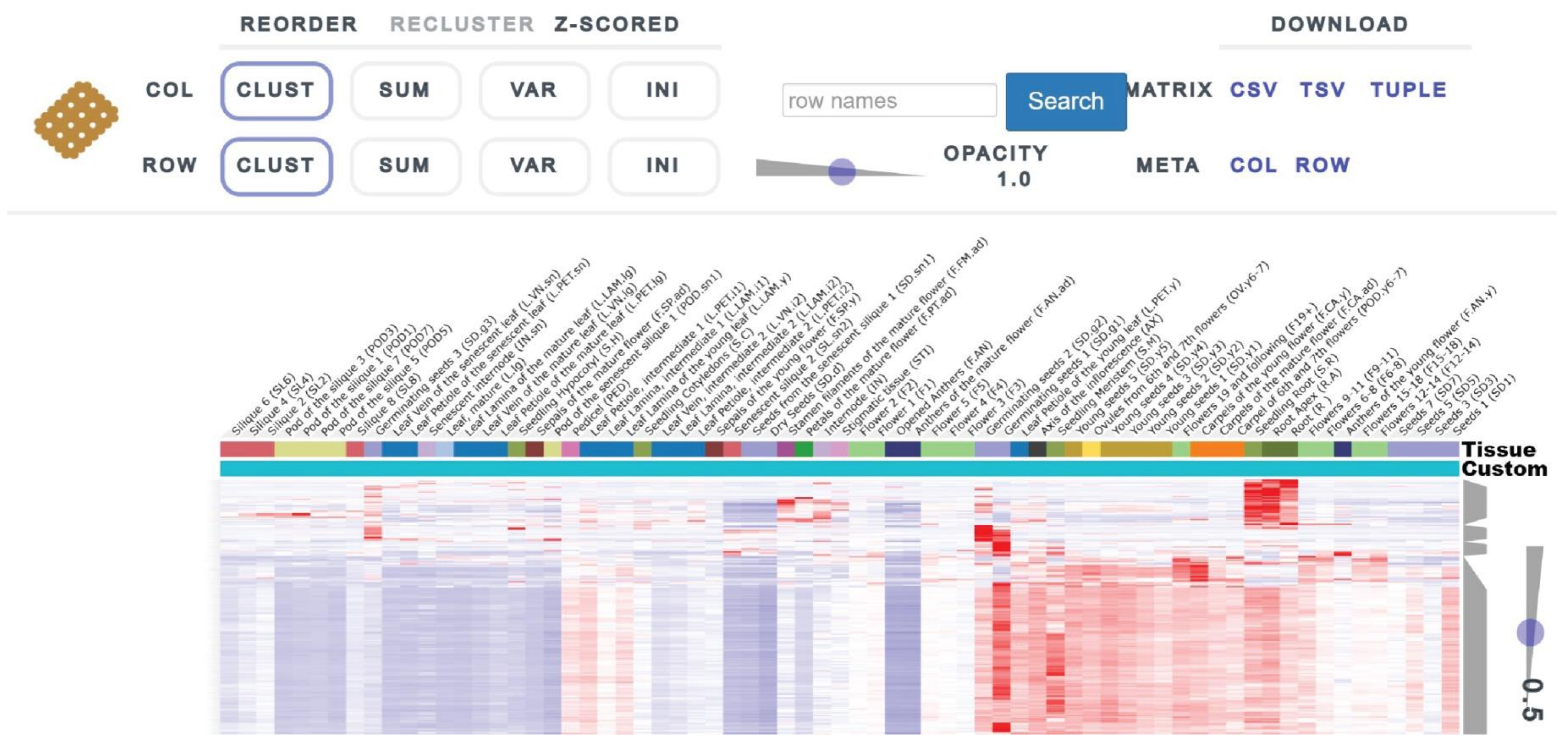

Supplemental Figure 5: Supports Figure 4. Example screenshot of Clustergrammer Jupyter notebook in which users can examine normalized expression values for mRNAs and lincRNAs across multiple stress and tissue atlases. See https://github.com/Evolinc/Brassicaceae_lincRNAs.

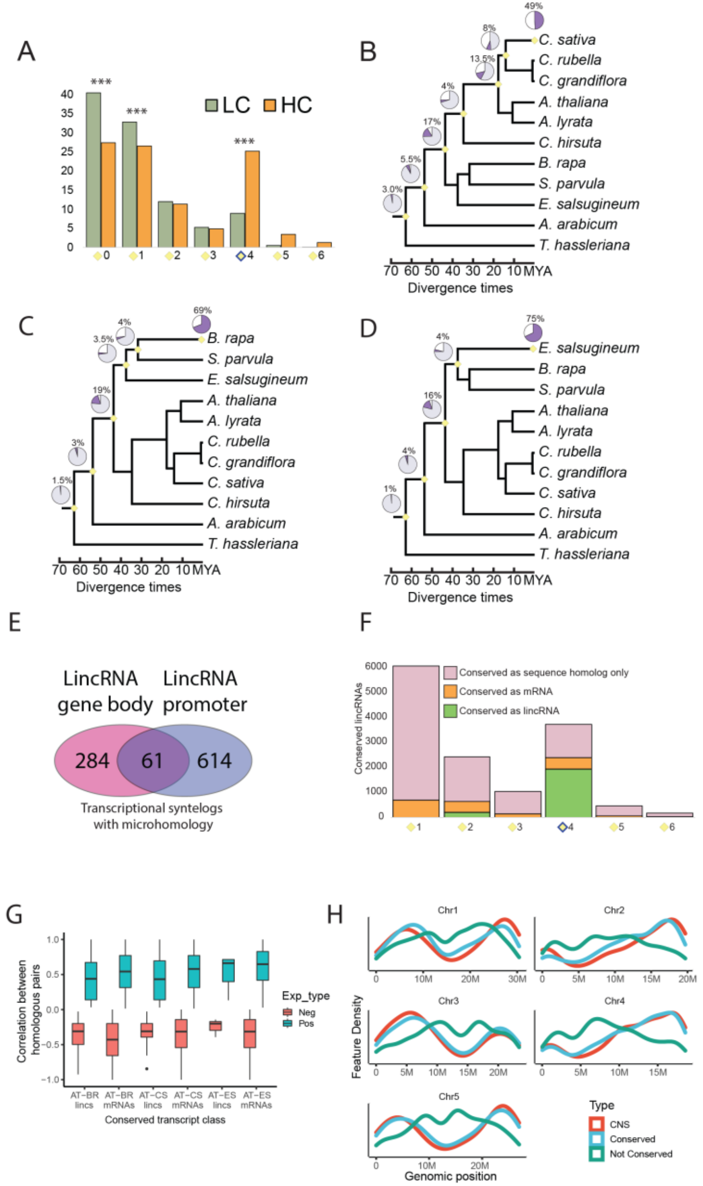

Supplemental Figure 6: Supports Figure 5. Evolutionary features of Brassicaceae lincRNAs. **A**) Percent of low-confidence and high-confidence Arabidopsis lincRNAs that are sequence conserved at each evolutionary node. Asterisks denote significant difference between observed homolog recovery for the two classes of lincRNAs (p-value <<< 0.01; Student's t-test). **B-D**) Conservation of lincRNAs from Camelina (**B**), Brassica (**C**), and Eutrema (**D**) across representative Brassicales. The purple wedge in the pie chart in each panel represents the percent of lincRNAs for which sequence homologs were recovered at each node, thus indicating that each lincRNA was conserved to at least that node. **E**) The number of Arabidopsis putative transcriptional syntelogs for which sequence homologs were identified using either the 5' or 3' 200 nts (gene body) or 200 bps of promoter (promoter) of lincRNAs from either Brassica or Eutrema. **F**) Number of Arabidopsis lincRNAs for which the sequence homolog corresponded to another lincRNA (green bar), mRNA (orange bar) or unannotated sequence (pink bar) at each particular node. **G**) Distribution and overlap between conserved non-coding sequences (CNS) and either conserved lincRNAs (Node 4 or deeper) or non-conserved lincRNAs (Node 0). **H**) Correlation of expression between homologous (lincRNA pairs) or orthologous (mRNA pairs) transcripts within our ONT RNA-seq data. Due to anti-correlation (for both mRNAs and lincRNAs), positive and negative correlations were separated.

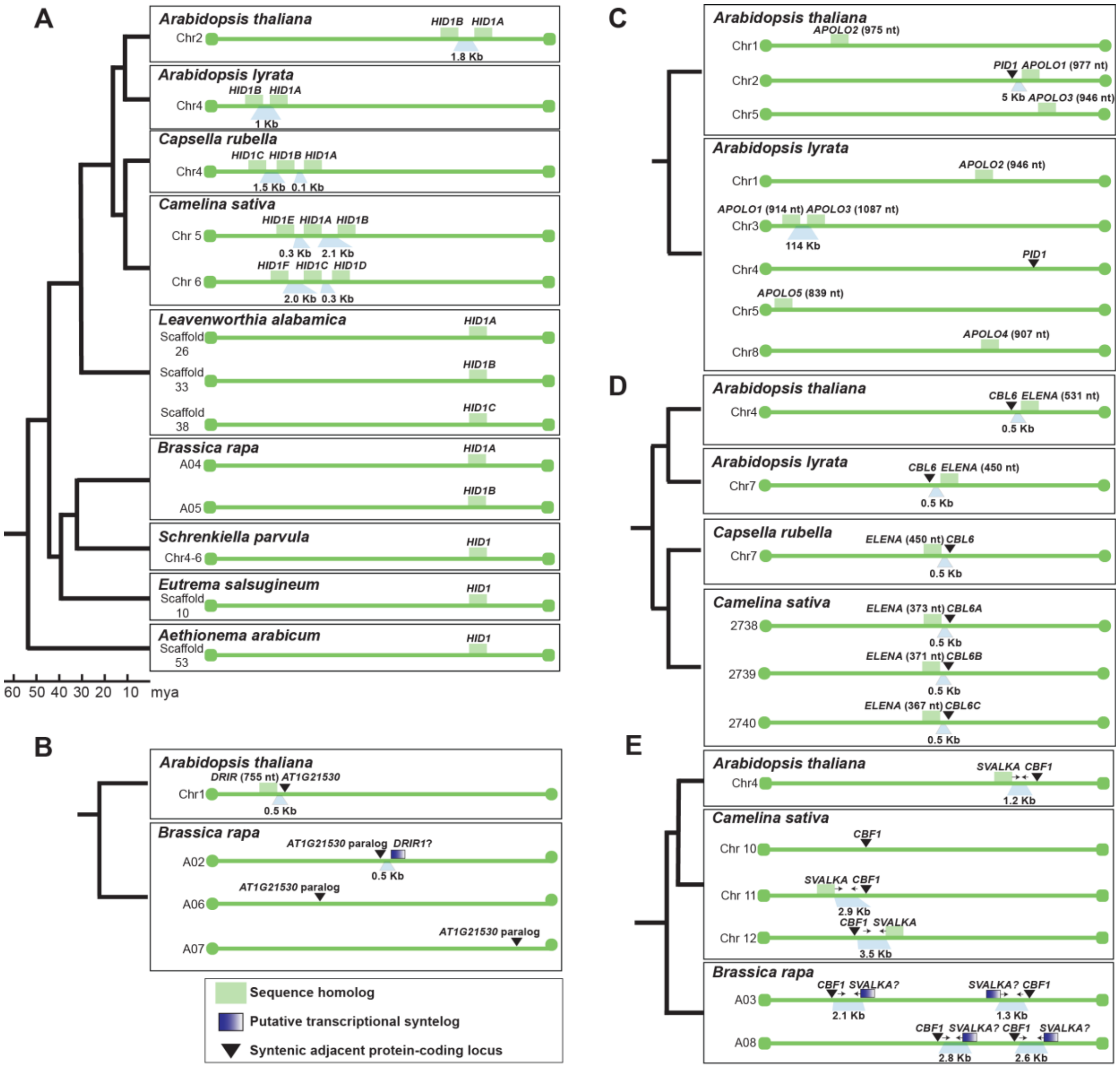

Supplemental Figure 7: Supports Figure 5. Evolution of functionally characterized lincRNAs. **A**) Schematic representing conservation of the HID1 locus across representative Brassicaceae. In Arabidopsis HID1A represents the published HID1 locus. All other green boxes represent loci inferred based on sequence homology and synteny. **B**) Conservation of DRIR1. Although no sequence homologs were identified for DRIR1, a putative transcriptional syntelog (Blue box) was identified in Brassica at a syntenic locus. **C**) Conservation of APOLO. Although APOLO sequence homologs (i.e., paralogs) were identified in Arabidopsis lyrata, none were adjacent to the PID1 ortholog, the protein-coding gene known to be regulated by APOLO in Arabidopsis thaliana. **D**) Conservation of ELENA. ELENA sequence homologs were identified in species as distantly related as Camelina sativa, where they were situated in syntenic positions adjacent to CBL6 orthologs. **E**) Conservation of SVALKA. Sequence homologs of SVALKA were identified in Camelina adjacent to CBF1. In Brassica, no sequence homologs were identified, but several putative transcriptional syntelogs were recovered adjacent to CBF1 orthologs.

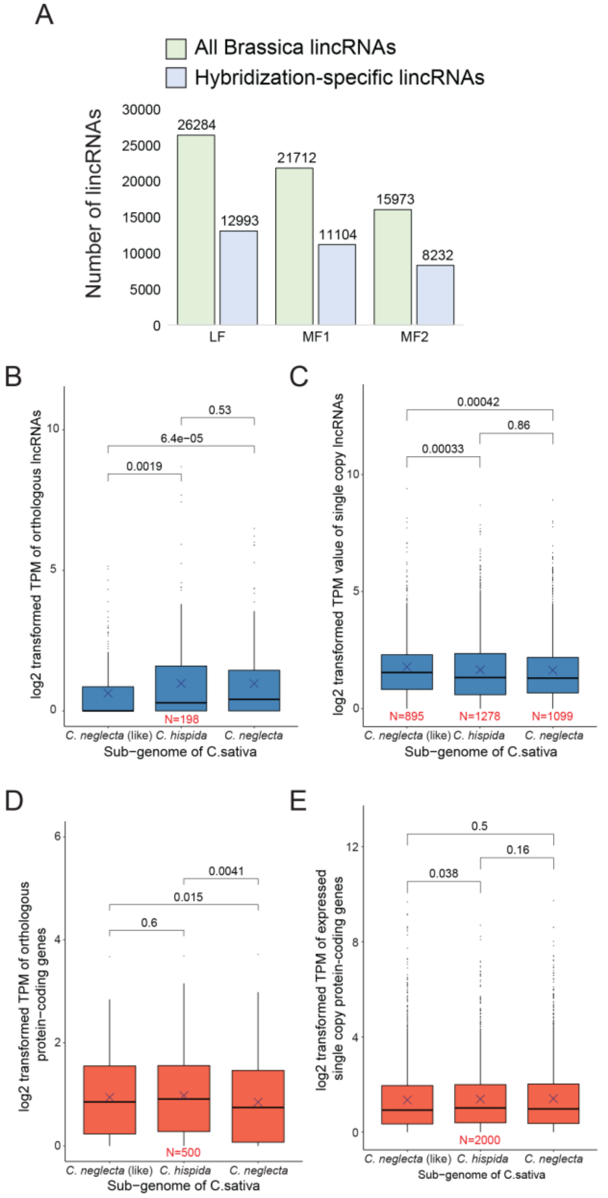

Supplemental Figure 8: Supports Figure 5. Subgenome expression dominance of Brassicaceae lincRNAs. **A**) Number of lincRNAs originating from each of the Brassica rapa subgenomes (LF = least fractionated, MF1 = medium fractionated, and MF2 = most fractionated). Coordinates for determining location of subgenomes within the Brassica genome were obtained from Cheng et al., 2013. **B**) Expression values of lincRNAs found in all three subgenomes of *C. sativa.* **C**) Expression values of lincRNAs specific to one subgenome. **D**) Expression values of protein coding genes found in all three subgenomes of *C. sativa.* **E**) Expression values of protein coding genes specific to one subgenome. Numbers represent Student's t-test *P* values between groups of expression values.

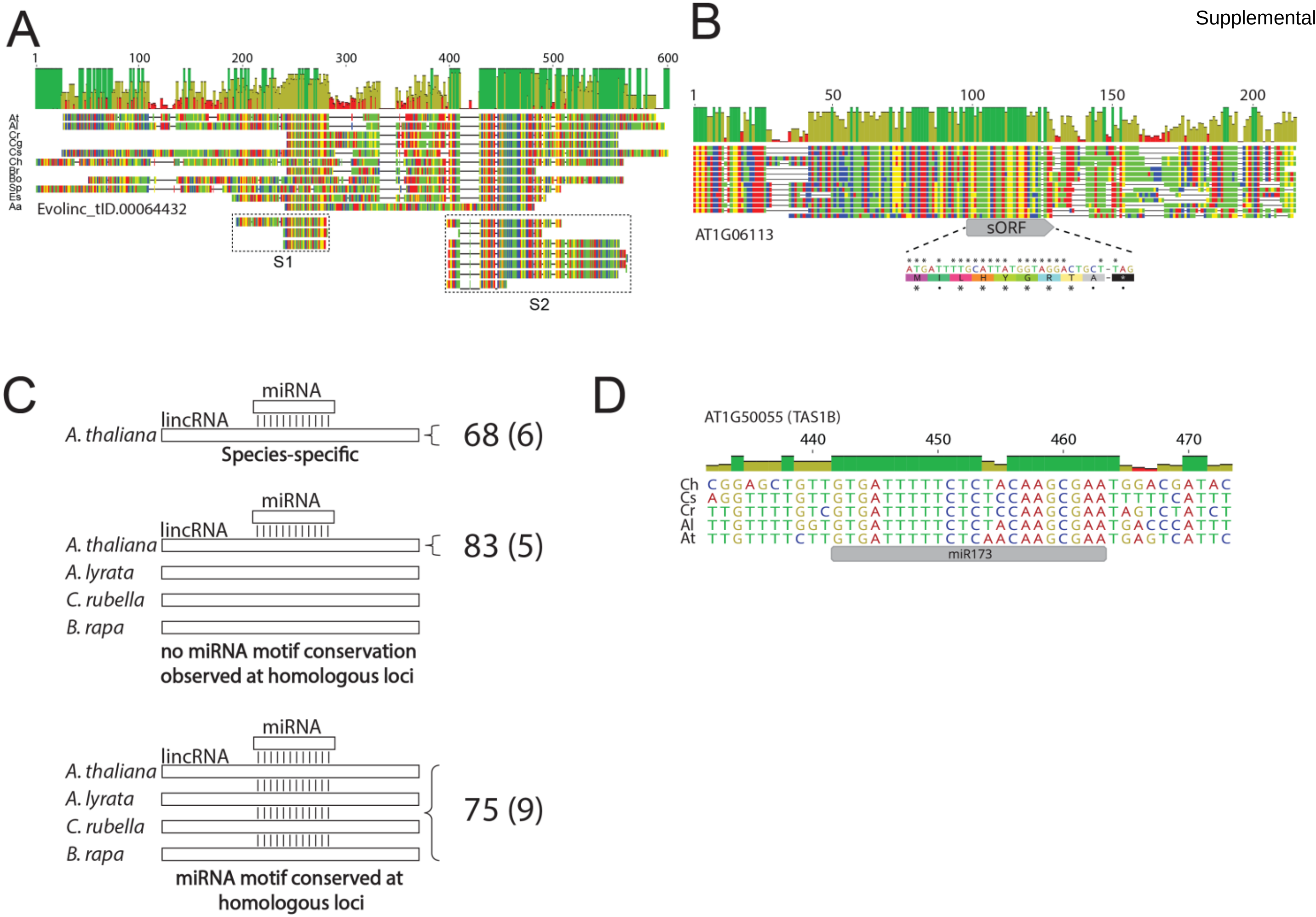

Supplemental Figure 9: Supports Figure 5. Examples of deeply conserved lincRNA motifs. **A**) Multiple sequence alignment (MSA) of a structured and protein-bound Arabidopsis lincRNA (Evolinc_SR_tID_00064432) where the functional motif and lincRNA are conserved to the same node. B) MSA of a sORF containing transcript, *AT1G06113*, where the lincRNA and sORF are conserved to the same node. Asterisks denote complete conservation. Single periods denote similar amino acids (e.g., I -> V) were observed at that site. **C**) Schematic demonstrating the number of putative miRNA binding motifs that were found to be either species-specific (lincRNA and miRNA motif are restricted to Arabidopsis), not conserved (lincRNA is conserved but conservation is not associated with the miRNA motif), or conserved. The number in parenthesis represent the number of lincRNAs with putative miRNA binding motifs that are also stress responsive. **D**) Example MSA of a lincRNA (Arabidopsis *TAS1B*) with a conserved miRNA binding motif in Cardamine, Capsella, and Arabidopsis.

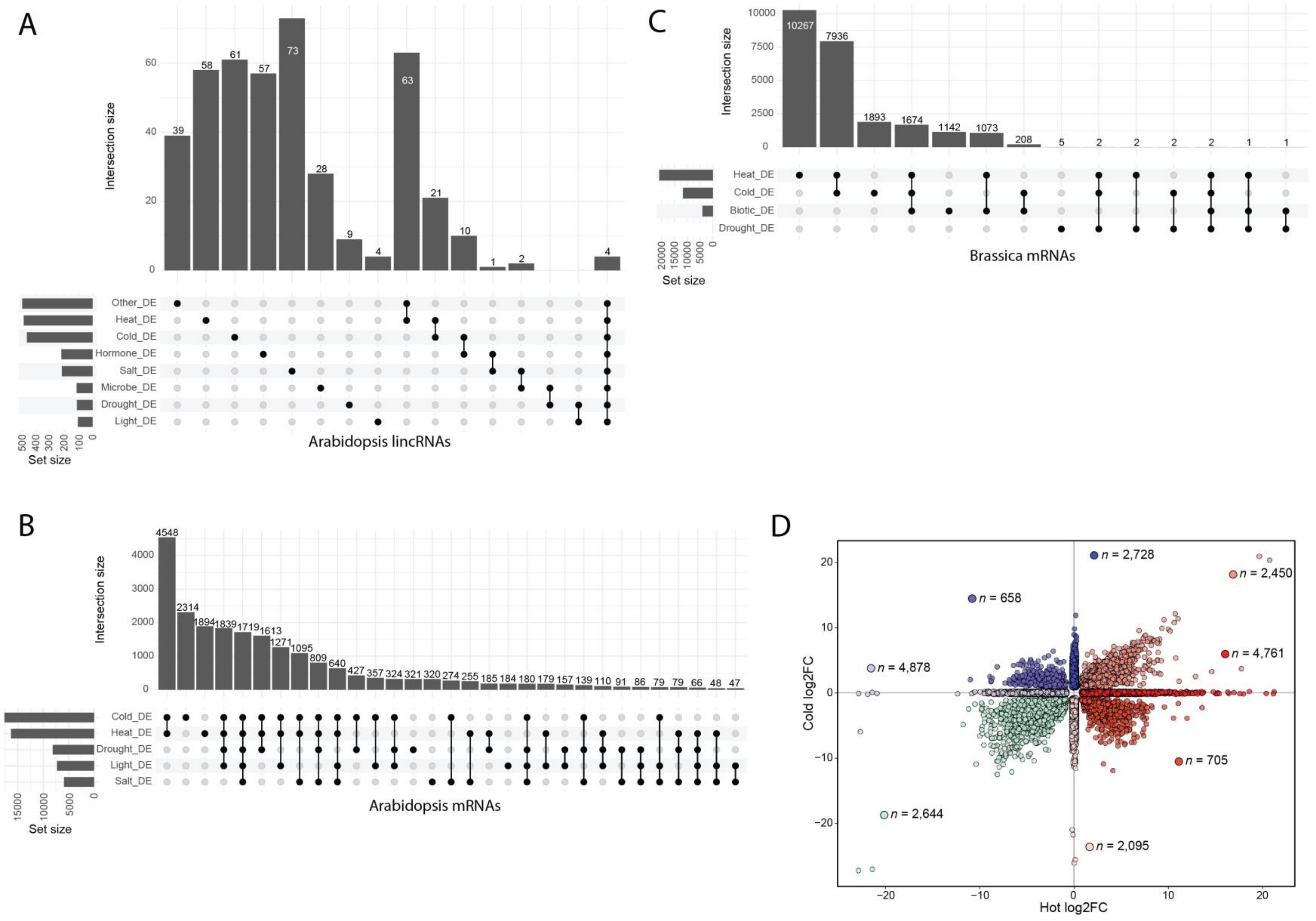

Supplemental Figure 10: Supports Figure 6. Differential expression during stress. **A**) Upset plot of Arabidopsis lincRNAs differentially expressed in a variety of broad stress categories (an expanded set of stresses compared to **Figure 6A**). **B**) Upset plot of Arabidopsis mRNAs found to be differentially expressed in various abiotic stresses. **C**) Upset plot of Brassica mRNAs found to be differentially expressed in various broad stress categories. **D**) Scatterplot comparing log2FC of Arabidopsis mRNAs in cold and heat stress when mRNAs are DE in both, or just a single stress.

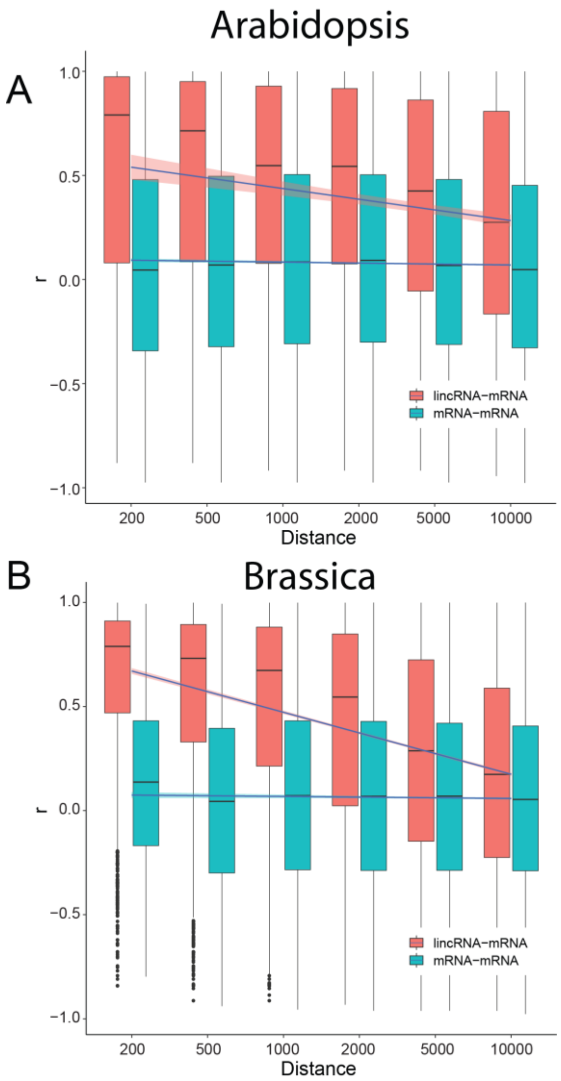

Supplemental Figure 11: Supports Figure 6. Gene expression correlation (Pearson) between lincRNA-mRNA and mRNA-mRNA pairs within defined distances in Arabidopsis and Brassica tissue atlases. **A**) Arabidopsis gene expression correlation of all expressed lincRNA/mRNAs with nearby expressed mRNAs within defined distances (x-axis). **B**) Brassica gene expression correlation of all expressed lincRNA/mRNAs with nearby expressed mRNAs within defined distances (x-axis). Note, all pairs within smaller distances are contained within larger distances.

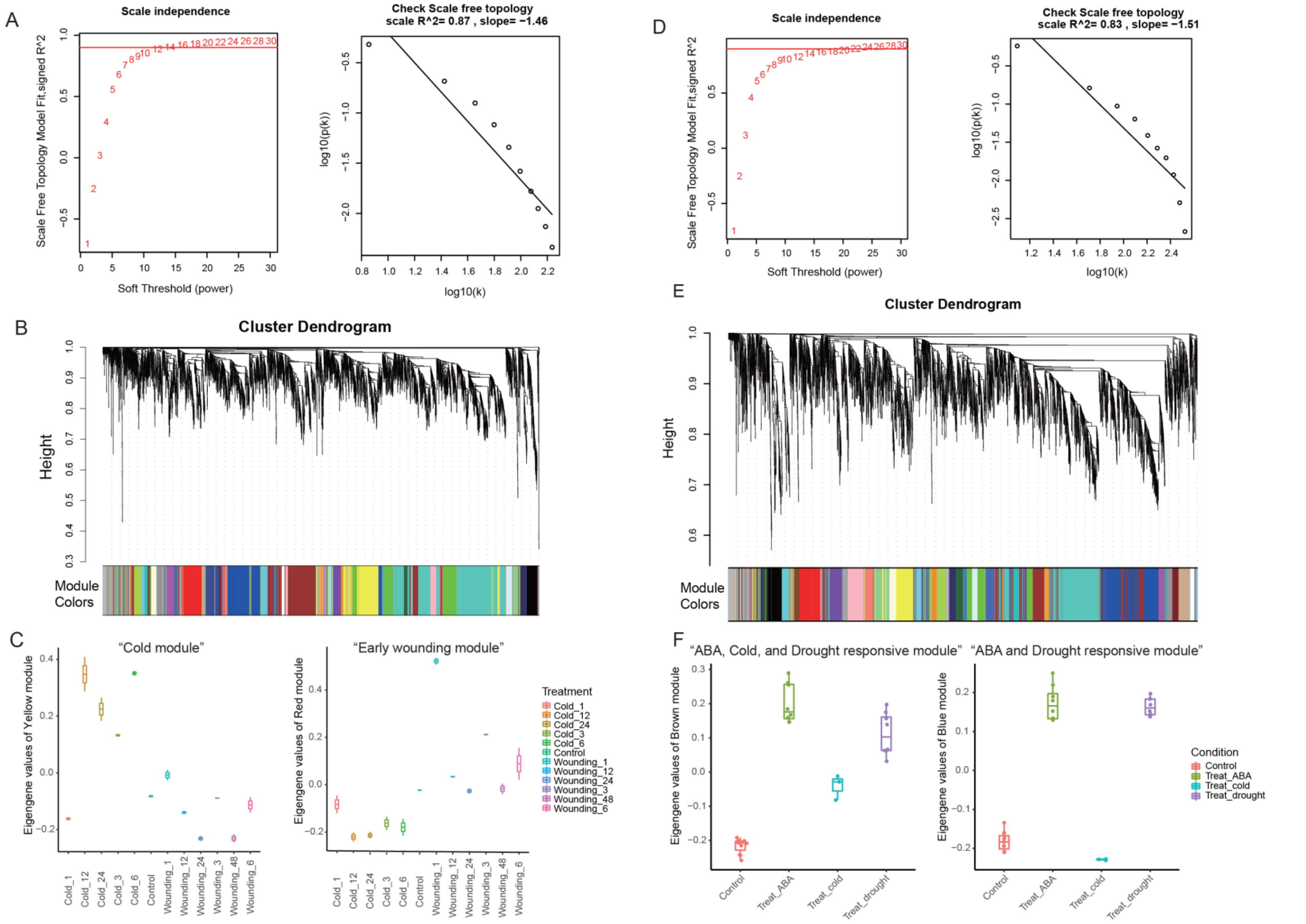

Supplemental Figure 12: Supports Figure 7. Assessment of parameters used to generate co-expression networks from the Klepikova stress dataset (**A-C**) and the combinatorial stress response dataset (**D-F**). **A** and **D**) Left, relationship between the approximation of a scale-free network iterated across different soft thresholds. Right, log-log plot of connectivity where the correlation coefficient represents the scale of free topology. **B** and **E**) Hierarchical clustering dendrogram of modules resulting from the WGCNA. **C** and **F**) Plot of Eigengene variation for genes across the different datasets for genes found in the different modules.

Supplemental Figure 13

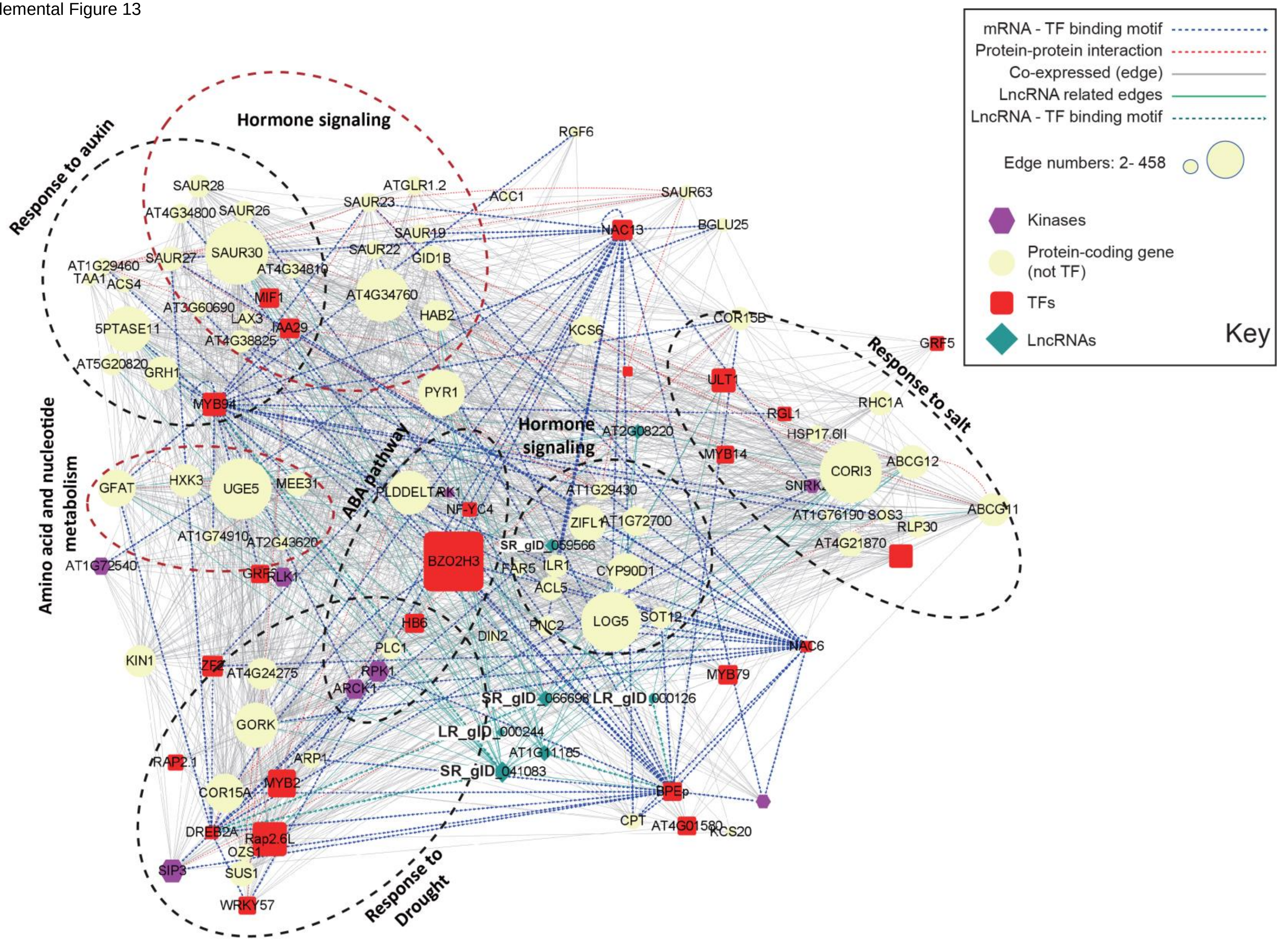

Supplemental Figure 13: Supports Figure 7. Gene network visualization of an ABA-responsive module. See key for more details. Dashed circles represent genes enriched for a particular (listed) KEGG or GO-term.

Supplemental Figure 14

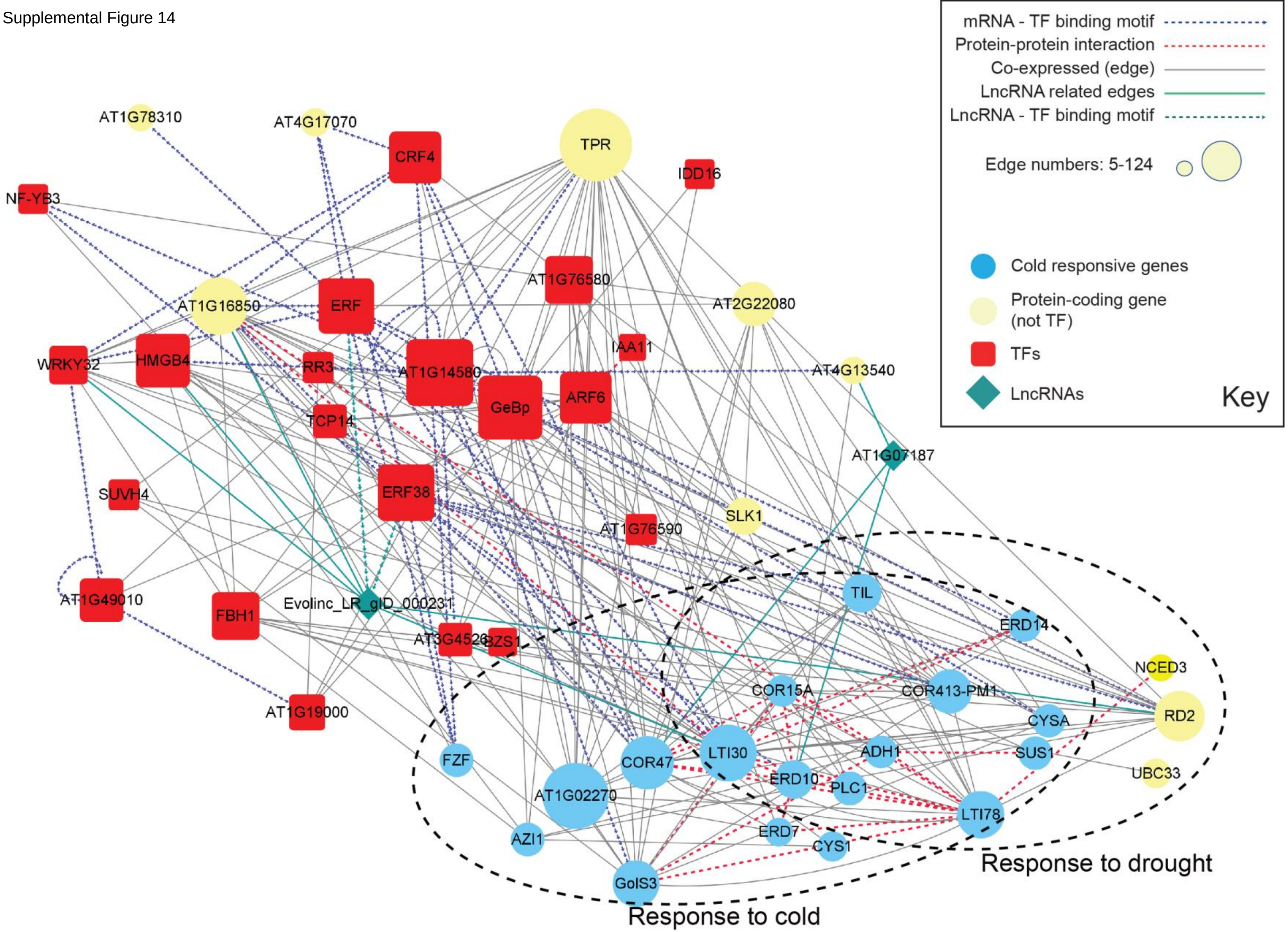

Supplemental Figure 14: Supports Figure 7. Gene network visualization of a drought and cold-responsive module. See key for more details. Dashed circles represent genes enriched for a particular (listed) KEGG or GO-term.
