# Supplementary material for: Identification and functional annotation of long intergenic non-coding RNAs in Brassicaceae
Source: Plant Cell. 2022 Jun 6;34(9):3233–60. doi: 10.1093/plcell/koac166 (PMC9421480; doi:10.1093/plcell/koac166)
Supplement: koac166_Supplementary_Data [file tpc21.00973.PeerReview.pdf › tpc21.00973.PeerReview.pdf]

# Identification and Functional Annotation of Long Intergenic Non-coding RNAs in Brassicaceae

Kyle Palos, Anna C. Nelson Dittrich, Li'ang Yu, Jordan R. Brock[2], Caylyn E. Railey, Hsin-Yen Larry Wu, Ewelina Sokolowska, Aleksandra Skirycz[1], Polly Yingshan Hsu, Brian D Gregory, Eric Lyons, Mark A. Beilstein, and Andrew D. L. Nelson

*Corresponding author: Andrew D. L. Nelson an425@cornell.edu*

## Review timeline:



| | | |
|---|---|---|
| **TPC2021-LSB-00973** | Submission received: | Nov. 10, 2921 |
| | 1st Decision: | Dec. 5, 2021 *revision requested* |
| **TPC2021-LSB-00973R1** | 1st Revision received: | March 14, 2022 |
| | 2nd Decision: | April 1, 2022 *accept with minor revision* |
| **TPC2021-LSB-00973R2** | 2nd Revision received: | May 5, 2022 |
| | 3rd Decision: | May 5, 2022 *acceptance pending, sent to science editor* |
| | Final acceptance: | May 29, 2022 |



**REPORT:** (The report shows the major requests for revision and author responses. Minor comments for revision and miscellaneous correspondence are not included. The original format may not be reflected in this compilation, but the reviewer comments and author responses are not edited, except to correct minor typographical or spelling errors that could be a source of ambiguity.)

**TPC2021-LSB-00973 1st Editorial decision – *revision requested*** **Dec. 5, 2021**

As you will read below, all three reviewers felt that the work is interesting and well done. There was some concern about whether it is overly descriptive; we recognize that you submitted as a "Large-Scale Biology" paper, which is meant to focus more on an examination of rich and deep datasets and may involve less hypothesis testing. However, the reviewers also commented that the paper is missing some examples of key biological insights that are potentially found in your data, but weren't drawn out in a way that would be of interest to the reader. That is, often these types of Large-Scale papers will include some vignettes about the most interesting observations that are made. But, we felt that you seem to stick to an overview of the dataset and don't really delve into specific examples, even though that could help to anchor your results. While we are not expecting you to start from scratch with in planta experiments, there are surely numerous loci that could be characterized as particularly informative examples, and we think that this what the reviewers are asking for. In addition, you will see below a number of specific comments from the reviewers that need to be addressed.

-------------------------------------------------------------------------- Reviewer comments:

Reviewer #1 (Comments for the Author):

This manuscript entitled "Identification and Functional Annotation of Long Intergenic Non-coding RNAs in Brassicaceae" by Palos et al. utilized all the public RNA-seq data from >16,000 experiments in four Brassicaceae species, and identified ~130,000 lincRNAs. They further analyzed these lincRNAs with respect to transcription, epigenetic marks, evolution, and the association with their flanking genes among the four species. The analysis is very comprehensive. I believe the data is useful for the community who are interested in non-coding RNAs. However, I feel the analysis is generally descriptive. I could not see the clear conclusions that the authors want to make. It would be good that the authors start from these lincRNAs, do some deep analysis, and identify some novel discoveries.

I believe annotation of lncRNAs is important, but I really want to see some functional analysis of these lincRNAs and their potential regulation of protein encoding genes. Although the authors did some association analysis of lncRNAs and flanking mRNAs in the section "Expression for a subset of LincRNAs is significantly correlated with adjacent mRNAs". The correlation is very descriptive. And also I can't get the point why the authors compare lncRNAs-mRNA pairs with mRNA-mRNA pairs within the same genomic regions. We know that mRNA-mRNA interaction nearby is not very common, so comparison of lncRNAs-mRNA pairs with mRNA-mRNA pairs may easily give you a significant

P value, which may not have any sense. I am thinking the authors can at least compare different RNA-seq libraries in different lines with and without some certain lncRNAs of the same species to see whether the expression values of their flanking genes are different or not. For example, the expression levels of some genes with lncRNAs nearby in some lines are significantly higher or lower than the expression levels of the same set of genes without lncRNAs nearby in other lines may indicate these lncRNAs have some roles in regulating these genes. Using these polymorphic lncRNAs may provide you some very interesting data. The analysis of the evolutionary part is also very descriptive. I have some specific comments as follows.

1. Results, page 5, Figure 1C. I am curious that there are not any overlaps with respect to the lincRNAs identified in these four different datasets (Previously annotated, Nanopore, Illumina HC, and Illumina LC). There should be some lincRNAs identified by different methods. Did the authors already remove overlaps? Actually it's good to show these in Venn diagrams to show the overlaps and total numbers.

2. Results, page 6, lines 184-185, "the transcript was conserved in sequence and syntenic at least one of 10 Brassicaceae genomes examined". I can't get the point here. If you want to talk about sequence conservation and synteny, I think at least you need two genomes. How can you tell synteny in one genome?

3. Results, page 6, lines 193-209. In this paragraph, the authors tried to compare lincRNAs with mRNAs to tell the false positives of mRNAs being annotated as lincRNAs. How about the mis-annotation of small RNA precursors, such as miRNA genes? Do these lincRNAs contain miRNA genes?

4. Results, page 7, lines 216-217, "Araport11 lncRNAs comprise 2,455 "lnc_RNAs", 286 "ncRNAs", and 726 "novel transcribed regions". What's the difference between "lncRNAs" and "lnc_RNAs"? Does "ncRNAs" indicate "non-coding RNAs"?

5. Results, page 7, lines 228-229, "transcripts to the same set of filters as our lincRNA dataset (ORF < 100 AA, longer than 200 nts, poor coding potential), which resulted in 571 NTRs annotated as lincRNAs and included in". How did the authors determine "poor coding potential"? And what does "NTRs" mean here?

6. Results, page 7, lines 242, "Of the 1,172 lincRNAs expressed > 0.1 TPM in seedling tissue". I have a concern regarding using "TPM" here. Given that lincRNAs differ in length, the authors should also normalize by the lincRNA length because they also compared different lincRNAs, such as in Figures 4 and 6.

7. Results, page 9, Figure 2. The titles of y axis should be added. Figure 2C, what does "unique miRNA motifs" mean?

8. Results, page 11, Figure 3. I have some concerns regarding Figure 3D. Based on some published literature (see below), in Arabidopsis and many other plant species, gene body and promoter methylation are different. Normally promoter methylation suppresses gene expression, but gene body methylation was found to be positively correlated with gene expression values. In contrast, the authors found that the methylation levels of the bodies of highly expressed genes of both lincRNAs and protein-coding loci were lower than those of non-expressed genes. Can the authors explain why they observed such patterns? I am thinking the body regions in Figure 3D include introns which normally have transposon insertions. Is that right?

Shohei Takuno and Brandon S. Gaut. "Body-Methylated Genes in Arabidopsis thaliana Are Functionally Important and Evolve Slowly", 2012. Mol.Biol. Evol.
Shohei Takuno and Brandon S. Gaut. "Gene body methylation is conserved between plant orthologs and is of evolutionary consequence". 2012. PNAS.

9. Results, page 20, lines 476-477 and 482-484. Because the authors did not provide enough introduction, I am unclear which the progenitor of the dominant subgenome in Camelina. From lines 476-477, "In Camelina, C. hispida mRNA paralogs are typically more highly expressed relative to those from the other two subgenomes", it gave me the feeling that C. hispida may be the progenitor of the dominant genome. However, from lines 482-484, "we see a

significant bias against expression of the paralogs from the C. neglecta (like) subgenome", it seems that C. neglecta (like) is the progenitor of the dominant genome in Camelina.

10. Results, page 21, line 512, "sORF within AT1G06113, which encodes for a nine amino acid peptide and lies within a region". Is AT1G06113 a protein encoding gene? Did the authors compare all your sORF with all protein encoding genes to make sure they are not parts of a protein encoding gene?

11. Results, page 22, line 554, "seedlings and roots treated with exogenous ABA (5-100uM) and identified 672 ABA-responsive", where dis the number "672' come from?

12. I am not sure if it is only me or not. The fonts of all the figures are really small. I even can't see clearly.

Reviewer #2 (Comments for the Author):
In this manuscript, Palos et al. investigated the characteristics of long intergenic non-coding RNAs (lincRNAs) in Brassicaceae. Based on publicly available RNA sequencing experiments and newly generated data, including ONT long read sequencing, they de novo predict lincRNA transcripts in four Brassicaceae species: Arabidopsis, Camelia, Brassica and Eutrema. Based on transcript length, number of introns, level of expression and presence in different experiments, they sub classify this new lincRNAs and the one of the current official annotations as high confident or low confident lincRNAs. Using these complete set of lincRNAs they then investigate the presence of translated small ORFs (sORFs), miRNA binding site, and structural motifs. They then studied the transcript characteristics (GC content, length, number of exons, tissue specificity of expression, epigenetic landscape) showing lincRNAs have specific characteristics compared to protein coding genes or transposable elements confirming previous studies. They then investigate the conservation of this lincRNAs among Brassicaceae either through sequence homology or synteny homology, showing as previously reported in other organisms little conservation of lincRNAs compared to protein genes. They further investigate lincRNA conservation by leveraging recent WGD available in Brassicaceae and showed similar behaviour of conservation than protein coding genes. Finally, they attached functional pathway annotations to the lincRNAs through analysing stress RNA-seq data, co-expression clustering with protein coding genes and correlation of expression with neighbouring protein coding genes.

The introduction is suitable and give background on long non-coding RNA and their difficult annotations. The procedure used are appropriate and major observations and claims are supported by the presented experiments. My main concerns in the article are the presentation of the data that need to be polished to be a valuable resource.

Major comments:
1) This article present an enormous analysis of public RNA-seq allowing the prediction of lincRNAs in a quite exhaustive way. This provides a very useful resource for the plant long non-coding RNA community. However, the presentation of the data do not correspond to the aim of the paper of sharing the data with the community:
- There is inconsistency between transcript naming between the different supplemental tables: TCONS/ XLOC (Supplementary table 3,4,5), Evolinc_SR_xxxx (Supplementary table 2). Please homogenize the data in all the article.
- I did not find the complete annotation of the transcripts (with the exon coordinates). Please provide a file in a used bioinformatic format for transcript description, such as GFF/GTF/BED, so it can be easily used in future expression investigations.
- Sup File 3: for the conservation analysis, in case the transcript is classified as "Homolog", no positional information is provided. The information is of importance since future transcript could be detected in uninvestigated condition at these positions. Please provide the full information.
- I did not find the syntenic conservation information: please provide the association between the transcript names in each species for this class of conservation.

2) In the same spirit, please provide a better description of the bioinformatic procedure used in the article:
- Indicate the precise version of the software and R packages used in the article : Hiat2, stringtie, minimpa2, bedtools, ... Some are present, put a lot are missing, notably for software.

- Indicate the parameters used for each software, or provide a repository with the scripts that will be able to reprocess the data. This may allow identifying bias in the processing of the data. The present description is not sufficient. There is a start, but the methods look as a draft (line 848 "Salmon using XXX parameters")
- I was not able to look at the CyVerse Data Store since no link was provided.

3) The authors showed conserved lincRNAs at the level of the sequence and of the synteny. In animal systems, it has been shown that conserved lincRNAs have conserved expression profile.
- What about the conserved lincRNAs identified in this study (sequence conserved and synteny conserved): are they expressed in the same tissues in the different species ?
- Is there any relation with the specificity of expression (TAU) and the conservation ?
- What about global conservation score such as PhastCons score (10.1093/molbev/mst082 and 10.1038/ng.2684)?
- Are the conserved lincRNAs (sequence conserved and syntenic conserved) present in wider conserved regions of the genome like in conserved non-coding sequences (such as described in 10.1038/ng.2684)?

Minor comments:
1) Some updated annotations of transcripts of Arabidopsis, notably using long reads, were recently published (10.1186/s12859-021-04208-2 and 10.1101/2021.09.02.458763). A comparison with your annotation should be provided, at least for the first one that is peer reviewed.
2) The authors clearly showed that CpG and in a less extent H3K27me3 profile are different between Arabidopsis and Brassica for coding genes and lincRNAs. What about transposable elements in Brassica that were only presented for Arabidopsis?
3) Fig 5A: node 0 show a very low percentage of lincRNAs I would have expected to find the 32.9% reported in the text. Could you explain this difference ?
4) Are the stress responsive lincRNAs really in higher number compared to the protein coding genes: is it a global transcriptome boost or is there any bias toward lincRNAs ?

Noticed typos and small suggested rearrangements:
- line 184 : "syntenic at least one of 10 Brassicaceae genomes examined" → "syntenic in at least one of 10 Brassicaceae genomes examined"
- line 594: I did not find the Supplemental Datasets 2. No supplemental Dataset 1 (neither in the text, neither provided)
- line 633: "Arabidopsis HRE2 ChIP-seq data in and" → "Arabidopsis HRE2 ChIP-seq data and"
- line 637-639: "Thus, these seven lincRNAs appear to be specifically regulated by HRE2 in response to hypoxic stress and may act as cis-regulatory elements (annotated in Sup File 2)." I did not find the information in that file.
- Fig S4C remove the trailing zeros in the TPM scale for female and leaf lincRNA: at first look the figure look larger than for the other and is therefore misleading.
- Fig S6: reorder the panels according to appearance in the text
- Sup FIle 5: formatting problem for Arabidopsis tab: missing header, not as complete as other tabs
- Sup File 6: please provide a small description of the datasets used: type of data (RNA-Seq, ChIP-seq, Bisulfite), title/one sentence description of the experiment.
- Cross-check the reference to supplementary material that are not always fitting: for example line 544 should be read Supplemental File 6 (?), line 629 should be read Supplemental Figure 12A-H

Reviewer #3 (Comments for the Author):
In the last decade, non-coding RNAs have emerged as critical elements for gene regulation, genome structure and stability, and many other biological processes. Even when previous works have already predicted and annotated many IncRNAs in different genomes, Palos and collogues present a deeper analysis, annotation and classification of lincRNAs in four Brassicaceae species. Even when I'm not a huge fan of "catalogue type" papers, this study took exciting approaches that are likely to be helpful for the scientific community. The article is well-written, there are some parts a little bit "boring", but this is expected and necessary for these kinds of papers. I don't find significant problems with the experimental designs, but I would like to suggest some aspects to improve/expand.

-There are many references cited in the text and missing in the references list, for example Volders et al., 2013; Zhao et al., 2019; Zhao et al., 2020. Perhaps it was a problem during Parsed Citations extraction.

-L186-187 (and lines 215): I'm wondering whether this criterion excludes, among the 10 experiments threshold, RNA-seq runs made on a single experimental setting/lab. It will be a more robust criterion if the lincRNA expression is detected in 10 completely independent experiments rather than replicas or associated RNAseq data sets from a single source. I'm wondering whether there is a bias here.

-Gagliardi 2019 and 2020 (rev) described an exciting feature of inverted repeated elements (IR) as non-coding regulatory elements. By definition, IR transcripts producing small-RNAs, such as the IRs mentioned above or pri-miRNAs, are lincRNAs and should be included in the intro. I'm wondering how many of the lincRNAs identified are pri-miRNAs, how many are TE-derived elements, and how many may have IR features. Is there a bias toward a specific TE family type?

-Considering the findings described from line 266, where you found miRNA targets in many lincRNA, my previous concern about a potential TE origin of these lincRNA is even more relevant. It was described that the miRNA targeting of TE transcripts triggers the formation of easiRNA (epigenetically active siRNAs) that in turn control TEs expression. Can you check how many of the miRNA targets has a TE origin? It would also be useful to check whether these lincRNAs are prone to produce secondary (phasiRNA/easiRNAs) by mapping small RNA seq data on these loci.

-Figure 3D and E, have you checked CHH methylation? It is likely that CHH methylation is higher in lncRNA than in coding genes, especially for TE-derived lincRNAs. I agree that if TGS occurs in these lincRNAs, as in TEs, you would also see CpG methylation, but CHH is more informative if the silencing is dynamic. Furthermore, I'm concerned that any methylation effect in a subpopulation of lncRNAs will be diluted among so many lincRNAs of different natures. This can be the case with TE-derived lincRNAs such as MITEs. I mean that for coding genes, you expect a more "homogeneous" pattern as the biology behind them is more similar. Contrary, within lincRNA, you include RNA molecules as lncRNAs, pri-miRNAs, TE-derived transcripts, phasiRNA-producing genes (such as TAS genes), all of them with very different regulation and maturation processes. Can you sort them into some subcategories and search for a pattern there?

-I'm wondering whether the "guilty by association" analysis, starting in line 568, differentiate co-expression caused by genome vicinity. Is there a difference in the result if you exclude adjacent genes, as are expected to be co-regulated due to genomic reasons, not necessarily functional reasons?

-I'm not a huge fan of the "Assigning putative function to Brassicaceae lncRNAs" section as it feels unvalidated biologically and somewhat speculative. Still, I can see that it may interest some readers. I think you should test how relievable this approach is to predict functions by scoring which percentage of lncRNAs of known functions are correctly associated with a functional prediction.

-Out of curiosity, I'm wondering whether you plan to work together with Araport to re-annotate non-coding elements and add the newly identified lincRNAs.

**TPC2021-LSB-00973R1 1st Revision received March 14, 2022**

Reviewer comments and **author responses**:

Reviewer #1:

Point 1. Results, page 5, Figure 1C. I am curious that there are not any overlaps with respect to the lincRNAs identified in these four different datasets (Previously annotated, Nanopore, Illumina HC, and Illumina LC). There

should be some lincRNAs identified by different methods. Did the authors already remove overlaps? Actually it's good to show these in Venn diagrams to show the overlaps and total numbers.

**RESPONSE: We apologize for not displaying this in an intuitive manner. We applied our hierarchical filtering method such that anything found by Nanopore was automatically HC, and then the successive filters were applied to Illumina (short read) derived transcripts. We have added a Venn diagram to Figure 1 and to Supplemental Figure 1 that describes the overlap of the different filters on the Illumina transcripts.**

Point 2. Results, page 6, lines 184-185, "the transcript was conserved in sequence and syntenic at least one of 10 Brassicaceae genomes examined". I can't get the point here. If you want to talk about sequence conservation and synteny, I think at least you need two genomes. How can you tell synteny in one genome?

**RESPONSE: We apologize for the confusing way in which we framed this statement. We indeed used two genomes to determine sequence conservation and synteny. In fact, we took all of the initial putative lincRNAs for each species and used them as query in Evolinc-II searches of the ten other species shown in Figure 5. Any mono-exonic lincRNA found in at least one other species aside from the query were added to the high confidence category. We have modified the text on lines 179-180 to more accurately reflect this.**

Point 3. Results, page 6, lines 193-209. In this paragraph, the authors tried to compare lincRNAs with mRNAs to tell the false positives of mRNAs being annotated as lincRNAs. How about the mis-annotation of small RNA precursors, such as miRNA genes? Do these lincRNAs contain miRNA genes?

**RESPONSE: This is an excellent point - we went back and scanned each of the genomes using rFAM to identify unannotated miRNA precursors. We then intersected those data with the lincRNA catalog from each species. For Arabidopsis, 26 lincRNAs overlapped with an rFAM predicted miRNA precursor, 69 for Camelina,124 for Brassica, and 44 for Eutrema. We have added text to lines 203-208 and updated Supplemental Files 2 and 6 to denote which lincRNAs may be miRNA precursors, and the associated miRNA class. We also used EDTA (Ou et al., 2019) to identify previously unannotated transposable elements across the four genomes, and then intersected those data with the lincRNA catalogs. Any lincRNAs that were => 50% TE content were labeled as TE-associated. These data are now reflected in text added at lines 284-294 and in Supplemental Figures 2B and 2C. In addition, we tested to see if any of the Arabidopsis lincRNAs overlapped with POL-IV dependent small RNA (sRNA) loci by reanalyzing sRNA-seq data from Zhou et al., 2022. The authors also performed RIP-sequencing targeting POL-V, which generates RNAs that are targets of sRNAs in the RdDM pathway. We used these data to infer which of the Arabidopsis lincRNAs were either targets of, or participants in, the RdDM pathway. Lastly, we tested whether the 1,041 TE-associated Arabidopsis lincRNAs were targets or participants of RdDM. These results are discussed on lines 295-312 and shown in Figure 2D.**

Point 4. Results, page 7, lines 216-217, "Araport11 IncRNAs comprise 2,455 "lnc_RNAs", 286 "ncRNAs", and 726 "novel transcribed regions". What's the difference between "IncRNAs" and "lnc_RNAs"? Does "ncRNAs" indicate "non-coding RNAs"?

**RESPONSE: We apologize for the confusion here. We used the exact nomenclature listed in the Araport11 annotation without fully describing the abbreviations. Araport11 contains a wide variety of non-coding RNAs without a clear distinction between the classes - in part because they were combining multiple annotation efforts. To harmonize the two annotations, we reassessed all classes of transcripts in the Araport11 annotation that might actually be lncRNAs. lnc_RNA was Araport11's denotation for long non-coding RNA (lncRNA), but did not distinguish whether a lncRNA was intergenic or overlapping another gene (such as an antisense transcript). Araport11's non-coding RNA (ncRNA) category also contained lincRNAs, as did the most enigmatic Araport11 class - the novel transcribed regions (NTRs). Araport11 left the NTRs as a grab bag of transcripts for which they were uncertain how to classify based on the data they had on hand. We reassessed these transcripts as we would any other novel transcript to determine if any of them could be classified as lincRNAs. We have updated the text to better explain what these abbreviations mean in lines 215-218.**

Point 5. Results, page 7, lines 228-229, "transcripts to the same set of filters as our lincRNA dataset (ORF < 100 AA, longer than 200 nts, poor coding potential), which resulted in 571 NTRs annotated as lincRNAs and included in". How did the authors determine "poor coding potential"? And what does "NTRs" mean here?

**RESPONSE: NTR = novel transcribed region. We filtered these transcripts based on length of transcript, length of open reading frame, and coding potential, using the coding potential calculator v2. CPC2 (Kang et al., 2017; NAR) is**

**a community accepted ML algorithm for determining whether a transcript is likely to be coding or not. While CPC2 is not perfect with shorter ORFs (as we see in Figure 2), it largely does a decent job - and using this algorithm provides continuity with other lincRNA identification efforts across eukaryotes. We updated the text to better explain what we were screening these NTRs for in lines 229-232.**

Point 6. Results, page 7, lines 242, "Of the 1,172 lincRNAs expressed > 0.1 TPM in seedling tissue". I have a concern regarding using "TPM" here. Given that lincRNAs differ in length, the authors should also normalize by the lincRNA length because they also compared different lincRNAs, such as in Figures 4 and 6.

**RESPONSE: Thank you for this comment. We used the "tximport" R package to generate normalized expression values. Specifically, we used the "lengthScaledTPM" option for the "countsFromAbundance" argument. The tximport documentation states that lengthScaledTPM is: "scaled using the average transcript length over samples and then the library size." We have updated the methods to reflect that TPM has been normalized for transcript length.**

Point 7. Results, page 11, Figure 3. I have some concerns regarding Figure 3D. Based on some published literature (see below), in Arabidopsis and many other plant species, gene body and promoter methylation are different. Normally promoter methylation suppresses gene expression, but gene body methylation was found to be positively correlated with gene expression values. In contrast, the authors found that the methylation levels of the bodies of highly expressed genes of both lincRNAs and protein-coding loci were lower than those of non-expressed genes. Can the authors explain why they observed such patterns? I am thinking the body regions in Figure 3D include introns which normally have transposon insertions. Is that right?

**RESPONSE: Thank you very much for catching this issue. After discussing the epigenetic aspect of our lincRNA story with colleagues, we realized that we 1) were using an outdated pipeline for processing epigenetic data, and thus plotting it incorrectly, and 2) weren't taking advantage of previously published comparative epigenetic data in Brassicaceae (Bewick et al., 2016). This publication, in part, examined DNA methylation (CpG, CHH, and CHG) as well as histone modifications in leaf tissue from both Arabidopsis and Eutrema, allowing us to make direct comparisons for how lincRNA loci might be regulated in these two species. We reprocessed these data using a similar approach to Bewick et al., and the new metaplots now reflect what we would expect for mRNAs from Arabidopsis and Eutrema. As the reviewer likely knows, Eutrema lacks gene body methylation (GBM), and this is reflected in the new plots in Figure 3D. We still observe distinct CpG patterns relative to mRNAs in Arabidopsis (notably, lack of GBM), and both Arabidopsis and Eutrema lincRNA loci are more heavily CpG methylated than their mRNA counterparts. To address the question of expression, with these revised data we compared genes that are expressed in leaves (Figure 3D) versus all genes (Supplemental Figure 3B). In addition, we also compared transposable elements in the two species, as well as those lincRNAs that contain high percentages of TE content (TE-associated lincRNAs; Supplemental Figure 3B). The epigenetic section of the manuscript was completely rewritten and can be found at lines 330-357.**

Point 8. Results, page 20, lines 476-477 and 482-484. Because the authors did not provide enough introduction, I am unclear which the progenitor of the dominant subgenome in Camelina. From lines 476-477, "In Camelina, *C. hispida* mRNA paralogs are typically more highly expressed relative to those from the other two subgenomes", it gave me the feeling that *C. hispida* may be the progenitor of the dominant genome. However, from lines 482-484, "we see a significant bias against expression of the paralogs from the *C. neglecta* (like) subgenome", it seems that *C. neglecta* (like) is the progenitor of the dominant genome in Camelina.

**RESPONSE: We apologize for the lack of clarity here. Although it isn't quite clear (as C. sativa is a relatively recent polyploid), it is thought that C. hispida is the "dominant" subgenome, whereas C. neglecta (like) is the least dominant. The subgenomes are not nearly as fractionated as those in *Brassica rapa*, therefore dominance is primarily inferred through expression. In this section, we wished to highlight that it appeared that groups of lincRNA paralogs that were retained in all three subgenomes were typically poorly expressed from the C. neglecta (like) subgenome - similar to mRNAs, whereas lincRNAs that were only found on the C. neglecta (like) subgenome (lost from the other two) where more highly expressed than lincRNAs that were only found on one of the other subgenomes. It's admittedly a nuanced point to make, but we felt that it was counterintuitive and thus worth pointing out. We have adjusted the text on lines 522-523 and 534-535 to specifically denote C. hispida and C. neglecta (like) as the dominant and least dominant subgenomes, respectively.**

Point 9. Results, page 21, line 512, "sORF within AT1G06113, which encodes for a nine amino acid peptide and lies within a region". Is AT1G06113 a protein encoding gene? Did the authors compare all your sORF with all protein encoding genes to make sure they are not parts of a protein encoding gene?

**RESPONSE: This is a very good question - None of the loci we determined as harboring sORFs are annotated as protein-coding genes. In part, we were excited about the notion of conserved, small ORFs within this set of lincRNAs - even if it did raise into question whether they were in fact non-coding. Given their conservation, these sORFs are likely performing some biological role that is not being investigated because their encoding transcripts have been overlooked by multiple annotation schemes. We did as the reviewer suggested and scanned each of the sORFs against known protein databases, and determined that only 10/151 showed sequence similarity (AA) to a known protein, whereas 47 displayed sequence similarity to a hypothetical protein - in most cases themselves, indicating that other groups had observed peptides mapping back to these loci but were unsure about their function. The other 94 sORFs, including the 9 AA one, shared no sequence similarity with known proteins or protein domains. To your original question - I think it likely (particularly given the robust nature of the riboseq data) that these transcripts are actually protein-coding genes. Even though this is a manuscript on lincRNAs, we wanted to report on these transcripts because we believe they should not be overlooked. We updated Figure 2A to reflect our BLASTp results, and for those sORFs which matched to known proteins, we have included those data in Supplemental File 4.**

Reviewer #2:

Point 1. This article present an enormous analysis of public RNA-seq allowing the prediction of lincRNAs in a quite exhaustive way. This provides a very useful resource for the plant long non-coding RNA community. However, the presentation of the data do not correspond to the aim of the paper of sharing the data with the community:

- There is inconsistency between transcript naming between the different supplemental tables: TCONS/ XLOC (Supplementary table 3,4,5), Evolinc_SR_xxxx (Supplementary table 2). Please homogenize the data in all the article.

**RESPONSE: Thank you for pointing out these issues. We have gone through the tables and manuscript to ensure consistency in naming.**

Point 2. Sup File 3: for the conservation analysis, in case the transcript is classified as "Homolog", no positional information is provided. The information is of importance since future transcript could be detected in uninvestigated condition at these positions. Please provide the full information.

**RESPONSE: We completely agree and apologize for not noticing that “Homolog” was not very useful without the corresponding genomic coordinates. We have included those coordinates as GFF files in four species-specific folders (Atha_lincRNAs_in_other_species, etc) in the CyVerse Data Store.**

Point 3. I did not find the syntenic conservation information: please provide the association between the transcript names in each species for this class of conservation.

**RESPONSE: We have added the putative transcriptional syntelogs for each species as an additional tab in Supplemental File 3 (“Transcr. Syn.”).**

Point 4. In the same spirit, please provide a better description of the bioinformatic procedure used in the article: Indicate the precise version of the software and R packages used in the article : Hiat2, stringtie, minimpa2, bedtools, ... Some are present, put a lot are missing, notably for software.

**RESPONSE: We apologize for this massive oversight. We have gone back through and tried to provide much more extensive information about the computational tools that were used and their parameters.**

Point 5. The authors showed conserved lincRNAs at the level of the sequence and of the synteny. In animal systems, it has been shown that conserved lincRNAs have conserved expression profile. What about the conserved lincRNAs identified in this study (sequence conserved and synteny conserved): are they expressed in the same tissues in the different species?

**RESPONSE: We addressed this using our ONT RNA-sequencing data and observed that indeed, there was**

**correlation in expression for conserved lincRNA homologs in a similar manner to orthologous mRNAs. These data are found in Supplemental Figure 6F and mentioned on lines 455-456.**

Point 6. Is there any relation with the specificity of expression (TAU) and the conservation?

**RESPONSE: Thank you for bringing this up. As reported in mammalian systems Hezroni et al., 2015, we do indeed see lower tissue specificity (broader expression) corresponding to deeper conservation. We have added these data to Figure 5B and mentioned them 453-454.**

Point 7. What about global conservation score such as PhastCons score (10.1093/molbev/mst082 and 10.1038/ng.2684)?

**RESPONSE: We attempted to utilize PhastCons for looking at lincRNA conservation but had difficulty recovering sufficient multiple sequence alignments that contained lincRNAs in each representative species. When generating multiple sequence alignments in Brassicaceae, intergenic regions were often removed from one or more species because of poor sequence similarity (in the chaining of large contigs step). In light of this, we attempted to address your point here by examining your next comment.**

Point 8. Are the conserved lincRNAs (sequence conserved and syntenic conserved) present in wider conserved regions of the genome like in conserved non-coding sequences (such as described in 10.1038/ng.2684)?

**RESPONSE: This is an excellent point, and indeed we see strong correlation between CNS identified by Haudry et al., 2013 and the conserved Arabidopsis lincRNAs. We present this analysis in Figure 5C and in Supplemental Figure 6G and described these results in lines 461-470.**

Reviewer #3:

Point 1. L186-187 (and lines 215): I'm wondering whether this criterion excludes, among the 10 experiments threshold, RNA-seq runs made on a single experimental setting/lab. It will be a more robust criterion if the lincRNA expression is detected in 10 completely independent experiments rather than replicas or associated RNAseq data sets from a single source. I'm wondering whether there is a bias here.

**RESPONSE: The 10 RNA-seq experiment criteria requires those to be from unique bioprojects. This has been made explicit in line 182-183. The reviewer is absolutely correct that there may be bias associated with large experiments from the same lab - we should point out that some of the experiments in which we observed low confidence (LC) lincRNAs for Arabidopsis and Brassica come precisely from large, single group (or single bioproject) experiments. For instance, many of the root tip/meristem-specific Arabidopsis lincRNAs come from sorted single cells coming from different regions of the root generated from the Benfey lab (Li et al., 2016). Whether these lincRNAs are root specific or reflect technical issues associated with the experiment (protoplasting, for instance) is unclear, as very few labs perform these types of experiments. Thus, we denote that they are low confidence, and context specific, and may or may not be prime targets for functional analysis.**

Point 2. Gagliardi 2019 and 2020 (rev) described an exciting feature of inverted repeated elements (IR) as non-coding regulatory elements. By definition, IR transcripts producing small-RNAs, such as the IRs mentioned above or pri-miRNAs, are lincRNAs and should be included in the intro. I'm wondering how many of the lincRNAs identified are pri-miRNAs, how many are TE-derived elements, and how many may have IR features. Is there a bias toward a specific TE family type?

**RESPONSE: Thank you for pointing out these interesting manuscripts. Because our four focal species do not have consistent TE annotation approaches (or are completely lacking TE annotations), we de-novo predicted TEs in each of our species using EDTA (https://github.com/oushujun/EDTA). EDTA annotates "intact" TEs as well as fragmented TEs (annotated TE classes include: inverted repeats, Gypsy LTR retrotransposon, target site duplications, mutator TIR TEs, CACTA TEs, copia LTRs, and many other broad and sub-classifications.) We then asked how many of these TEs overlapped the lincRNAs in each of the four species. In Arabidopsis, for instance, the most common TE type overlapping lincRNAs was the helitron class (n = 628) (Data shown in Supplemental Figure 2B and 2C, Supplemental File 6, with text updated on lines 285-295). We did observe TIRs within our lincRNAs, such as CACTA/Copia/Gypsy/hAT, although they were less prevalent in all species except for Eutrema. Interestingly, this may reflect how plant lincRNAs have traditionally been annotated - which was to ignore those that were TE-associated (thus avoiding topics such as exaptation). Thus, in Arabidopsis where TEs are heavily annotated, and**

**lincRNAs cannot overlap known annotated genes/TEs, we naturally see a much lower number of TE-associated lincRNAs than we do in the other Brassicaceae where TEs are much more poorly annotated. We initially chose to avoid TE-associated lincRNAs because there is no clear distinction between a TE and a lincRNA in the mammalian field. We arbitrarily chose a => 50% cutoff for a lincRNA to be called TE-associated, but are open to modifying this definition.**

Point 3. Considering the findings described from line 266, where you found miRNA targets in many lincRNA, my previous concern about a potential TE origin of these lincRNA is even more relevant. It was described that the miRNA targeting of TE transcripts triggers the formation of easiRNA (epigenetically active siRNAs) that in turn control TEs expression. Can you check how many of the miRNA targets has a TE origin? It would also be useful to check whether these lincRNAs are prone to produce secondary (phasiRNA/easiRNAs) by mapping small RNA seq data on these loci.

**RESPONSE: In continuation from the above comment, this has now been addressed in Figure 2. Given the miRNA targets we were looking for, very few (5) TE-derived lincRNAs are associated with miRNAs using psRNApredict. We also determined that there is an even smaller number of (Arabidopsis) small RNA associated TE-derived lincRNAs. We anticipate that this number will likely go up in the other species where there is a much higher number of TE-derived lincRNAs.**

Point 4. Figure 3D and E, have you checked CHH methylation? It is likely that CHH methylation is higher in lncRNA than in coding genes, especially for TE-derived lincRNAs. I agree that if TGS occurs in these lincRNAs, as in TEs, you would also see CpG methylation, but CHH is more informative if the silencing is dynamic. Furthermore, I'm concerned that any methylation effect in a subpopulation of lncRNAs will be diluted among so many lincRNAs of different natures. This can be the case with TE-derived lincRNAs such as MITEs. I mean that for coding genes, you expect a more "homogeneous" pattern as the biology behind them is more similar. Contrary, within lincRNA, you include RNA molecules as lncRNAs, pri-miRNAs, TE-derived transcripts, phasiRNA-producing genes (such as TAS genes), all of them with very different regulation and maturation processes. Can you sort them into some subcategories and search for a pattern there?

**RESPONSE: Thank you for your comments and questions. For a multitude of reasons (see responses to Reviewers 1 and 2) we completely revamped our epigenetics section. Using this comparative dataset between Arabidopsis and Eutrema we now have an "Apples to Apples" comparison that fortuitously includes ChG and ChH DNA methylation information. Using your suggestion we were able to pull out the TE-associated lincRNAs and examine their methylation states relative to the other classes of lincRNAs, mRNAs, and TEs. The TE-associated lincRNAs in both Arabidopsis and Eutrema show much higher methylation (in all contexts) relative to other lincRNAs or mRNAs. Despite this elevated methylation that looks somewhat like TEs, we do not see the characteristic "bump" for the TE-associated lincRNAs like we do for TEs. We considered breaking down the metaplots further, but they start to become quite noisy because we are dealing with fewer and fewer loci. These new information have been included in Supplemental Figure 3 and in Figure 3 and on lines 330-357.**

Point 5. I'm wondering whether the "guilty by association" analysis, starting in line 568, differentiate co-expression caused by genome vicinity. Is there a difference in the result if you exclude adjacent genes, as are expected to be co-regulated due to genomic reasons, not necessarily functional reasons?

**RESPONSE: This is an excellent question. We (naively) initially expected to observe a large number of adjacent mRNA - lncRNA pairs present together in our gene networks, thinking that these lncRNAs could be potential cis-regulators. To our surprise, we observed very few lncRNA-mRNA pairs that displayed consistent changes in expression over multiple experiments such that they would statistically cluster together into a WGCNA module. To visualize this a bit, we have provided detailed gene networks for a few of the more interesting modules where we observe lncRNAs with multiple connections and to be direct targets of transcription factors (through public DAP-seq data) known to be associated with the response in question (e.g., wounding in Figure 7).**

Point 6. I'm not a huge fan of the "Assigning putative function to Brassicaceae lncRNAs" section as it feels unvalidated biologically and somewhat speculative. Still, I can see that it may interest some readers. I think you should test how relievable this approach is to predict functions by scoring which percentage of lncRNAs of known functions are correctly associated with a functional prediction.

**RESPONSE: Unfortunately, there are very few functionally validated lincRNAs in Arabidopsis - thus we have an n =**

**1 for functionally tested lincRNAs that fall into an expected module (SVALKA falling into a cold-stress module within the Klepikova stress dataset). To address this we added two additional bits of information. We provided a bit more insight into how our stress-associated lincRNAs that fall into interesting co-expression modules might function through a more detailed network analysis (Figure 7 and Supplemental Figures 12-14). We also took a cohort of conserved Arabidopsis lincRNAs (conserved to at least Brassica, but most to Aethionema, Node 5), that were most highly expressed in germinating seeds or developing embryos and were predicted to be associated with embryogenesis or germination-related GO terms, and for which T-DNA insertions were available from ABRC. We then did a simple germination screen to test the degree to which our predictions were correct (that these lincRNAs would have some role in germination). Out of eight tested lincRNA loci (one for which we had two independent mutant alleles), we saw 10-100 % decreases in germination as compared to WT (Figure 7D/E). We don't make strong conclusions in the manuscript about what these lincRNAs are doing, but we attempt to say that the additional annotations associated with each lincRNA (where available) can be invaluable in developing functional hypotheses - or at least pointing researchers to a particular developmental stage to start looking for phenotypes**

Point 7. Out of curiosity, I'm wondering whether you plan to work together with Araport to re-annotate non-coding elements and add the newly identified lincRNAs.

**RESPONSE: Thank you for bringing up this important point. We have been in contact with Tanya Berardini at TAIR, as well as Nick Provart with the EFP Browser. Tanya is ready and willing to upload our lincRNAs as a track on the Araport genome browser. She has been waiting until we were close to publication so that there would be a paper with which to associate the track. Nick is likewise ready to incorporate the expression data associated for our lincRNAs (for all four species) into the appropriate EFP browser; he has just been waiting for TAIR to assign IDs to these loci.**

| TPC2021-LSB-00973R1 2nd Editorial decision – *accept with minor revision* | April 1, 2022 |
|---|---|

On the basis of the advice received, the board of reviewing editors would like to accept your manuscript for publication in The Plant Cell. This acceptance is contingent on revision based on the comments of our reviewers. In particular, I ask that you address the remaining minor comments and questions of reviewer 1 and 2.

-------------------------------------------------------------------------- Reviewer comments:
Reviewer #1 (Comments for the Author):
I am happy with this revised version. I think the authors addressed most of my questions that I asked in the last version. I have some further questions for this new version.

1. Results, page 5, lines 189-192, "In total, we identified 9,306 Arabidopsis, 58,155 Brassica, 13,163 Camelina, and 20,744 Eutrema high confidence lincRNAs (HC-lincRNAs), while 8,867, 11,977, 7,432, 4,893 lincRNAs were categorized as LC-lincRNAs, respectively." I suggested the authors to compare the locations of these HC-lincRNAs and LC-lincRNAs. For examples, how many HC-lincRNAs and LC-lincRNAs are in gene bodies (introns and exons), 5'UTR, 3'UTR, 2 kb upstream and downstream of genes, intergenic regions, and transposable elements (TEs), etc.

2. Results, page 10, line 345, "lincRNA loci show a consistent decrease in CpG methylation across the gene body, a pattern". Probably it is better not using "gene body", which may confuse people think of protein encoding gene body. I feel "lincRNA body" or "lincRNA gene body" may be clearer. Please also change other places accordingly.

3. Figure 3D and Supplemental Figure 3B, if the lincRNAs in Figure 3D are separated into TE-associated and TE-not-associated, what does the pattern look like? In addition, for H3K27me3 in Arabidopsis in Figure 3E, can the authors explain why Arabidopsis lincRNAs have higher level of H3K27me3?

4. Results, page 13, lines 434-436, "lincRNAs in two different ways: 1) sequence and synteny-based conservation (sequence homologs), and 2) transcriptional and synteny-based conservation in the absence of identifiable sequence similarity (transcriptional syntelogs)." I have some concerns with respect to the transcriptional syntelogs. If you just consider the lincRNAs in the syntenic blocks with high expression values as syntelogs, this gave me the feeling that you may compare apples with oranges. You may not compare the same thing. What I suggest is that you can lower the standard for similarity, but not absent of sequence similarity. LincRNAs may have higher sequence divergence

than protein encoding genes, which actually is interesting to test. I understand that. However, if just based on transcription values, it is hard to say they are syntelogs based on the definition of syntelogs.

5. Results, page 16, lines 526-547. Actually, it is better to compare the expression of the duplicated lincRNAs in addition to single copy lincRNAs, which may give you a much better idea to tell the dominant genome and the recessive genome.

6. Results, page 19, lines 620-621, "In both species, heat/cold responsive lincRNAs were predominantly upregulated by heat and repressed by cold". This is surprising to see. How can this be explained?

Reviewer #2 (Comments for the Author):
The authors fully addressed all my concerns. I find the addition of the functional validation of lincRNA mutants a good addition to the manuscript. I noticed minor points that could be corrected.

- In Supplemental File 4, tab 3 (structural conservation tab), still some XLOC/YLOC escape the consistency renaming (also on CyVerse).
- The Supplemental File 12, tab 2 (Brassica WGCNA Modules) is empty (also on CyVerse).
- The expression level filtering is missing from the legend of Figure 3D & E (> 1TPM) and Supplemental Figure 3B (all level). Without this information, the reader will be surprised by the difference of profile for Eutrema lincRNAs.
- I did not find any comment on the profile of H3K9me2 and H3K27me3 marks (from Figure 3E). They are marked as removed from this manuscript version.
- Review the accordance of the figure in the text and the Figure 5A: line 500 7.6% or 7.8% as in figure 5A? line 505 43% or 41%?

Reviewer #3 (Comments for the Author):
The authors have addressed my concerns, and have significantly improved the manuscript. I would like to thank the authors for the time invested in addressing my concerns and answering my questions.

**TPC2021-LSB-00973R2 2nd Revision received May 5, 2022**

Reviewer comments and **author responses**:

Reviewer #1:

Point 1. Results, page 5, lines 189-192, "In total, we identified 9,306 Arabidopsis, 58,155 Brassica, 13,163 Camelina, and 20,744 Eutrema high confidence lincRNAs (HC-lincRNAs), while 8,867, 11,977, 7,432, 4,893 lincRNAs were categorized as LC-lincRNAs, respectively." I suggested the authors to compare the locations of these HC-lincRNAs and LC-lincRNAs. For examples, how many HC-lincRNAs and LC-lincRNAs are in gene bodies (introns and exons), 5'UTR, 3'UTR, 2 kb upstream and downstream of genes, intergenic regions, and transposable elements (TEs), etc.

> **RESPONSE: We inherently excluded lncRNAs that were antisense or sense overlapping in our analysis and focused primarily on the intergenic class of lncRNAs. We failed to identify any intron-derived lncRNAs - perhaps due to the short intron size in our focal species. As the reviewer points out, there may be biases for proximity of HC or LC-lincRNAs relative to protein-coding genes or TEs. We plotted the distribution of distance from lincRNA to nearest protein-coding gene and observed no significant difference (when effect size was taken into consideration). We did however observe differences between overall lincRNA populations between species, perhaps reflecting aspects of the genome in those species (e.g., genome size, average intergenic space between protein-coding genes, etc.). These data are now included in Supplemental Figure 1D-E and described in lines 190-192.**

Point 2. Results, page 10, line 345, "lincRNA loci show a consistent decrease in CpG methylation across the gene body, a pattern". Probably it is better not using "gene body", which may confuse people think of protein encoding gene body. I feel "lincRNA body" or "lincRNA gene body" may be clearer. Please also change other places accordingly.

> **RESPONSE: To avoid potential confusion we have changed mentions of GBM that refer to lincRNAs as "lincRNA**

**body" methylation. We also make a similar distinction for TEs.**

Point 3. Figure 3D and Supplemental Figure 3B, if the lincRNAs in Figure 3D are separated into TE-associated and TE-not-associated, what does the pattern look like? In addition, for H3K27me3 in Arabidopsis in Figure 3E, can the authors explain why Arabidopsis lincRNAs have higher level of H3K27me3?

**RESPONSE: The lincRNAs in Figure 3D are those that are expressed above 1 TPM in the paired RNA-seq experiment (leaf tissue). As the reviewer suggested, we separated these lincRNAs out, for Arabidopsis and Eutrema, into TE-associated (18 and 47, respectively) and non-TE associated (437 and 596, respectively). The most obvious difference in Arabidopsis is potentially higher methylation levels upstream of the lincRNA body relative to non-TE associated lincRNAs. For Eutrema, there appears to be a distinct lack of the decrease in methylation across the lincRNA body. However, due to the small sample size we don't feel like we can make any strong statements. These data are now displayed in Supplemental Figure 3C-D and described on lines 354-357.**

**For the H3K27me3 marks, which are normally associated with inactive promoters, we feel that the differences observed between mRNAs and lincRNAs may be explained by differences in average expression in this tissue. Although we selected only loci expressed above 1 TPM, the median TPM for Arabidopsis and Eutrema mRNAs was 11.7 and 9.79, respectively, whereas it was 1.8 and 3.0, respectively, for lincRNAs.**

Point 4. Results, page 13, lines 434-436, "lincRNAs in two different ways: 1) sequence and synteny-based conservation (sequence homologs), and 2) transcriptional and synteny-based conservation in the absence of identifiable sequence similarity (transcriptional syntelogs)." I have some concerns with respect to the transcriptional syntelogs. If you just consider the lincRNAs in the syntenic blocks with high expression values as syntelogs, this gave me the feeling that you may compare apples with oranges. You may not compare the same thing. What I suggest is that you can lower the standard for similarity, but not absent of sequence similarity. LincRNAs may have higher sequence divergence than protein encoding genes, which actually is interesting to test. I understand that. However, if just based on transcription values, it is hard to say they are syntelogs based on the definition of syntelogs.

**RESPONSE: We agree with the reviewer that lincRNAs originating from a syntenic region of a genome may not be indicative of conserved function. In our original analysis we inferred lincRNAs in two species to be "transcriptional syntelogs" if they were adjacent to the same, orthologous protein-coding genes and if they were transcribed in the same orientation in both species. As the reviewer points out, this approach is fraught with caveats and we tried to point out these transcripts while not over stating what they might be doing. We assessed the transcriptional syntelogs (TSs) as the reviewer suggested - chopping each Brassica and Eutrema TS into three blocks - the first 5' 200 nts, the 3' 200 nts, and 200 bps of the promoter region (immediately upstream of the 5' block) and using these different blocks as query in a reduced stringency BLASTn search (1 e-10) of the Arabidopsis genome. We then determined whether BLAST returns were associated with the genomic region surrounding the corresponding Arabidopsis TS. Out of the 3,560 TS found between Arabidopsis and lineage II representatives (Eutrema and Brassica), 345 shared some sequence similarity within the lincRNA gene body (5' or 3' blocks), whereas 675 shared sequence similarity within the promoter region (with 61 lincRNAs overlapping between the two categories). We have described this additional analysis in lines 463-466 and have highlighted those lincRNAs in Supplemental File 7.**

**We also realize that the use of the word "syntelog" when referring to these lincRNAs may unnecessarily confuse readers, as it already has a well-established definition. We removed this term and instead refer to these lincRNAs, or lincRNA loci, as "syntenic but sequence divergent" (SBSD lincRNAs).**

Point 5. Results, page 16, lines 526-547. Actually, it is better to compare the expression of the duplicated lincRNAs in addition to single copy lincRNAs, which may give you a much better idea to tell the dominant genome and the recessive genome.

**RESPONSE: Indeed, the reviewer is correct that comparing multicopy lincRNAs (where paralogs have been retained) may provide information as to subgenome dominance. When comparing lincRNA paralogs retained in all three subgenomes we observe that lincRNAs arising from the C. neglecta (like) subgenome are much less expressed relative to paralogs from the other two subgenomes. However, lincRNAs that are retained as single copy loci in the C. neglecta (like) subgenome, but were lost in the other two subgenomes, are on average expressed higher than single copy lincRNAs from the other two subgenomes (lines 536-541). The difference in expression isn't a lot, but it is significant when considering sample size, therefore we reported it. However, we don't have a completely satisfactory explanation. Particularly since the pattern is counter to what has been observed for protein-coding genes in the C. neglecta (like) subgenome.**

Point 6. Results, page 19, lines 620-621, "In both species, heat/cold responsive lincRNAs were predominantly upregulated by heat and repressed by cold". This is surprising to see. How can this be explained?

**RESPONSE: We apologize. For aesthetic reasons (crowding) the initial plots (Figure 6C-D) were examining only those lincRNAs that were impacted by both cold and heat, but not those that were strictly impacted by one or the other. We modified the plot to include all lincRNAs DE by heat, cold, or both, and restricted the comparison to two time points for both figures. This expanded version of the plot reinforces the reviewer's comment - that many more lincRNAs seem to be repressed by cold rather than upregulated, whereas the impact of heat stress leads to more lincRNAs being up rather than downregulated in Arabidopsis (326 vs 138), and a similar number of lincRNAs activated or repressed (777 vs 704) in Brassica. This is in contrast to mRNAs from the same experiments (Supplemental Figure 10D), suggesting this may be a meaningful observation. This pattern is not specific to the original Klepikova tissue atlas (Klepikova et al., 2016), but is also observed in a later, cold-specific dataset (Klepikova et al., 2019), as well as in Brassica (shown in Figure 6C). However, we unfortunately don't have a clear explanation for why cold would specifically repress more lincRNAs than are activated - but perhaps this is a phenomenon worth investigating further in a more detailed mechanistic study.**

**TPC2021-LSB-00973R2 3rd Editorial decision – *acceptance pending*** **May 5, 2022**

We are pleased to inform you that your paper entitled "Identification and Functional Annotation of Long Intergenic Non-coding RNAs in Brassicaceae" has been accepted for publication in The Plant Cell, pending a final minor editorial review by journal staff. At this stage, your manuscript will be evaluated by a Science Editor with respect to its presentation of scientific content, compliance with journal policies, and presentation for a broad readership.

**Final acceptance from Science Editor** **May 29, 2022**
